# An ancient genome duplication event drives the development and evolution of spinnerets in spiders

**DOI:** 10.1126/sciadv.adw2173

**Published:** 2026-01-14

**Authors:** Fengyuan Li, Han Yang, Yiming Zhang, Shuhui Wang, Qi Gu, Meiming Wu, Pengyu Jin, Xin Huang, Yu Zhong, Xianting Huang, Yejie Lin, Xinyue Guo, Yunyun Li, Wei Zhang, Shuqiang Li

**Affiliations:** ^1^ State Key Laboratory of Animal Biodiversity Conservation and Integrated Pest Management, Institute of Zoology, Chinese Academy of Sciences, Beijing 100101, China.; ^2^ State Key Laboratory of Gene Function and Modulation Research, School of Life Sciences, Peking University, Beijing 100871, China.; ^3^ College of Life Sciences, Anhui Normal University, Wuhu, Anhui 241000, China.; ^4^ State Key Laboratory of Membrane Biology and the State Key Laboratory of Stem Cell and Reproductive Biology, Institute of Zoology, Chinese Academy of Sciences, Beijing 100101, China.; ^5^ Peking-Tsinghua Center for Life Sciences, Academy for Advanced Interdisciplinary Studies, Peking University, Beijing 100871, China.; ^6^ Medog Biodiversity Observation and Research Station of Xizang Autonomous Region, Nyingchi 860711, China.

## Abstract

Key appendage innovations have driven the origin and expansion of arthropods, such as spinnerets enabling spiders to occupy three-dimensional space and diversify into more than 53,000 species. Here, we investigate the genetic basis of spinneret emergence in spiders by examining the complex history and functional importance of arachnid genome evolution. Using chromosome-scale genomes from newly sequenced spiders and the whip scorpion, we integrate evidence from macrosynteny and phylogenetic analyses to provide further strong support for a whole-genome duplication (WGD) event that occurred during early Arachnopulmonata evolution. Following this event, the *abdominal-A* gene pair not only exhibits functional divergence but also jointly facilitates the emergence of spinnerets. Furthermore, we integrated single-cell transcriptomic analyses and functional validation to confirm that the *dachshund-1* gene also regulates spinneret development. The network of duplicated gene pairs may form a cornerstone in the origin and evolution of key morphological traits, revealing that the long-term effects of ancient WGDs on innovation and diversification also occurred in arthropods.

## Teaser

A genome duplication event during the Silurian played a crucial role in the evolutionary emergence of spinnerets in spiders.

## INTRODUCTION

Spider silk is one of the strongest natural materials known, with exceptional tensile strength and elasticity (*1*). The ability of spiders to produce a wide variety of silk types for different ecological purposes has made spider silk a subject of considerable interest in materials science and synthetic biology (*2*). Recent advancements have focused on understanding the molecular mechanisms underlying spider silk production, with the ultimate goal of harnessing this knowledge for artificial silk production.

Spiders extrude and manipulate silk through specialized structures called spinnerets, located at the fourth and fifth opisthosomal segments (O4–O5). Previous studies speculated that the evolution of spider spinnerets is related to the duplicated Hox genes along the anterior-posterior axis (*3*–*5*) and limb-patterning genes along the proximal-distal axis (*6*–*8*). Gene duplication events, particularly involving Hox genes, explain major leaps in the evolution and adaptive radiation of species, as seen in genes such as *Hox3* (*9*), *fushi-tarazu* (*10*), and *Shx* genes (*11*). While spiders have two Hox gene clusters, the precise way in which Hox gene pairs contribute to the formation of spinnerets is still debated. Schwager *et al.* (*12*) found evidence for a single whole-genome duplication (WGD) in an ancestor of the arachnopulmonates. Early support for this hypothesis came from the discovery that spiders have two Hox gene clusters, in contrast to the single cluster found in most other arthropods, along with the observation that spider homeobox genes, regulatory genes, and microRNAs are duplicated, and each homeobox cluster is typically distributed on different chromosomes (*13*–*17*). More recently, genome-wide analyses have revealed systemic duplication of gene families and patterns of macrosynteny between the whip scorpion and other arthropods, further supporting the WGD model (*18*). An alternative suggestion is that these patterns may instead result from a series of large segmental duplications (*19*), but this lacks evidence. In addition, the development of the animal body plan is governed by gene regulatory networks (GRNs), which must undergo changes in their architecture during evolution (*20*). However, the evolutionary processes that govern the gene networks controlling spinneret development are also not fully understood. Specifically, there are two competing scenarios—the gill hypothesis and the leg hypothesis—reflecting how Hox genes modify complex gene networks to transform one tissue into another. The gill hypothesis, proposed by Damen *et al.* (*5*), suggests that spinnerets are derived from ancestral arthropod gills based on the expression of gill-associated genes *nubbin* and *apterous* (*ap*) in the spinnerets of mid-late spider embryos. In contrast, the leg hypothesis posits that the spinnerets evolved from the ancestral legs because the leg patterning genes *extradenticle* (*exd*), *homothorax* (*hth*), *dachshund* (*dac*), and *Distal-less* (*Dll*) and their paralogs are expressed in the spinnerets (*3*, *21*). However, hypotheses currently rest on gene expression data alone. None of the studies to date have used the RNA interference (RNAi) or CRISPR-Cas9 system to test the two competing hypotheses or to investigate the functions of duplicated *abd-A*. Thus, the proposed roles of these duplicated Hox and limb-patterning genes remain speculative. Advancing our understanding of these processes is hindered by limited knowledge of the genomic and cellular mechanisms involved in these unique appendages. One hurdle has been the challenge of tracing the genomic changes underlying the expression patterns and spinneret-specific cell types. Second, limited genetic tools constrain the exploration of the genotype-to-phenotype relationship.

**Fig. 1.**
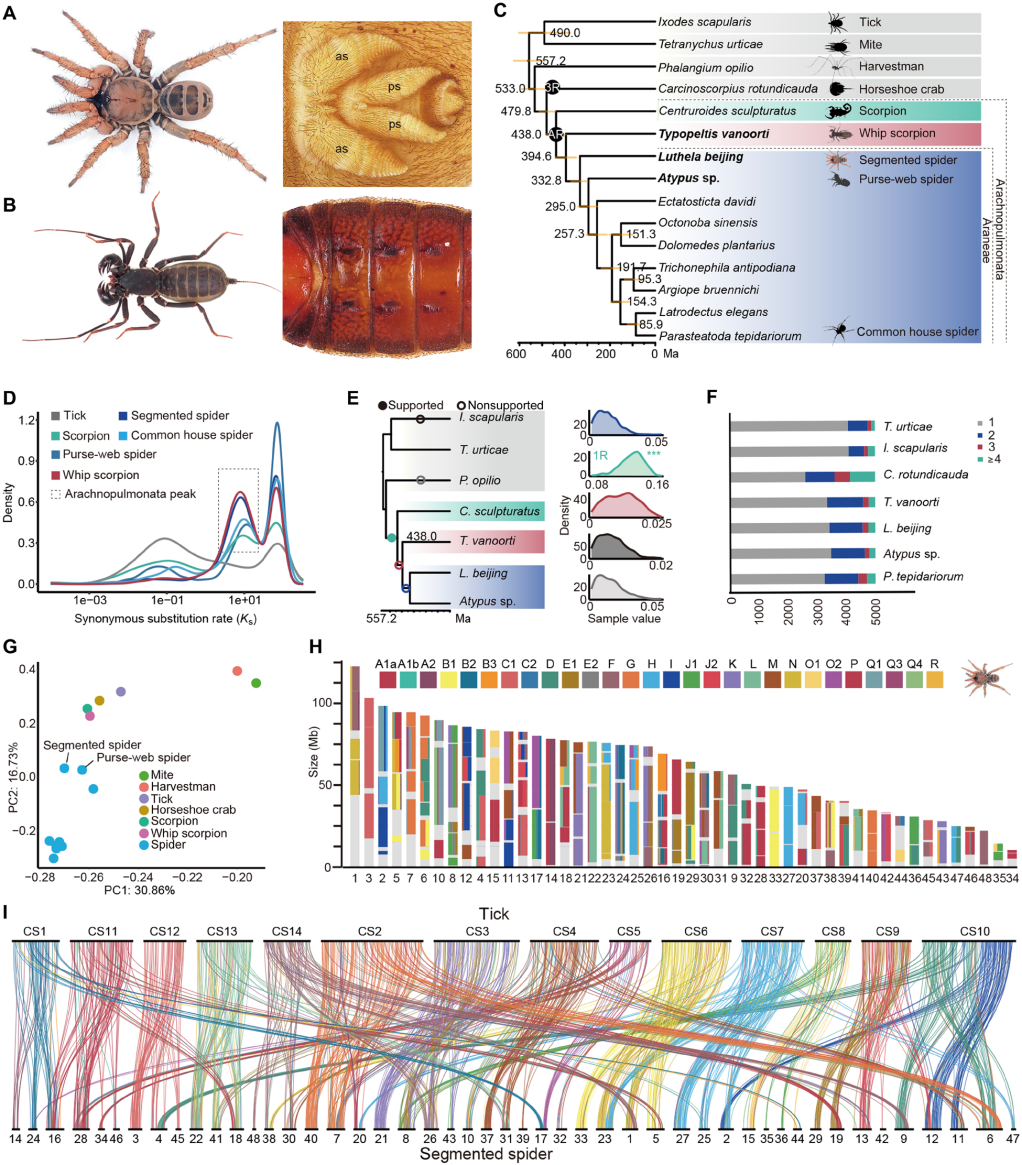
Genome duplication and conserved synteny in the segmented spider, the purse-web spider, and whip scorpion. (**A**) Adult female segmented spider (*L. beijing*) habitus and spinnerets; as, anterior spinneret; ps, posterior spinneret. (**B**) Adult female whip scorpion (*T. vanoorti*) habitus and opisthosomal somites 1 to 6. (**C**) Interrelationships and divergence times of selected arachnid species. WGD events, including AR of Arachnopulmonata and 3R of the horseshoe crab, are indicated with black circles. (**D**) Synonymous substitution rate (*K*_s_) distributions of paralogous genes in arachnid species are represented with colored lines as indicated. (**E**) Species phylogeny and AR hypothesis tested with WHALE using 4166 gene families. The hypothesis is indicated by circles on the corresponding branches, with supported WGD indicated with a solid circle. Kernel density estimates for the marginal posterior distributions for the retention rates are shown. Asterisks indicate distributions significantly different from 0 (Bayes factors BF_Null_vs_WGD_ < 10^−3^), which correspond to the supported WGD event. (**F**) Respective gene family composition of orthogroups in selected arachnid species indicating differential genome-wide duplication events. (**G**) Principal components (PC) analysis of arachnid gene complements demonstrates that *L. beijing* and *Atypus* sp. belong to early branching lineages of spiders and retain more ancestral arachnid orthogroups than true spiders. (**H**) Fraction of genes derived from each bilaterian-cnidarian-sponge Linkage Group in the segmented spider chromosomes (*22*). (**I**) Karyotypic correspondence between *L. beijing* and *I. scapularis* shows that rearrangements of the segmented spider chromosomes result from fission. Lines connect orthologous genes and are colored according to the ancestral linkage groups.

In this study, we integrated genomic, single-cell transcriptomic, and large-scale functional approaches in the spider lineage to systematically investigate the origin of spinnerets and notably enhance our understanding of the evolutionary novelties that emerged following an ancient WGD (*22*). We first generated chromosome-scale assemblies of the segmented spider *Luthela beijing* (Fig. 1A), the purse-web spider *Atypus* sp., and the whip scorpion *Typopeltis vanoorti* (Fig. 1B) to establish a comprehensive picture of arachnid genome evolution by means of macrosynteny analysis. We found that these newly sequenced species share slow-evolving genomes and described remnants of a Silurian duplication event in Arachnopulmonata. We then explored the impact of this WGD event on the evolution of spider spinnerets. Functional genomic analyses revealed both *abd-A-1* and *abd-A-2* linked to the origin of the spinneret, suggesting that these genes have evolved a previously unknown but overlapping function. In addition, single-cell differential expression analysis allowed us to pinpoint key candidate genes likely involved in spinneret determination, supporting the leg hypothesis regarding the evolutionary origins of the spider spinneret. Consistent with this, the analysis of *dac-1* expression profiles and the phenotypes of CRISPR-Cas9–induced mutants further confirmed the redeployment of a leg-patterning gene to novel regions of the opisthosoma. Overall, our study provides a comprehensive understanding of the genomic and cellular mechanisms of spinnerets and proposes a model where ancient WGDs accelerated functional innovations in spider evolution. By gaining a complete understanding of the spinning organs, we unlock their potential for advancements in both medical and industrial fields.

## RESULTS

### The evolution of spider genomes

To trace the evolutionary history of spider genomes and investigate its implication for key innovations, we generated chromosome-scale genome sequences of an ancient sister group of spiders, *T. vanoorti*, and two early branching lineages of spiders, *L. beijing* and *Atypus* sp. Our *T. vanoorti* assembly spans 3.50 Gb and includes 26 large chromosomal scaffolds; our *L. beijing* assembly includes 48 chromosome-scale scaffolds, representing a 3.46-Gb assembly, and our *Atypus* sp. assembly spans 875.51 Mb and includes 21 large chromosomal scaffolds (figs. S1 to S3 and tables S1 to S3). The relatively large number of chromosomes in the early branching lineages of spiders and whip scorpion reflects the ancestral conditions among spiders. We annotated 26,689 genes for the segmented spider, 19,161 genes for the purse-web spider, and 22,184 genes for the whip scorpion. Analysis using Benchmarking Universal Single-Copy Orthologs (BUSCO) showed a high degree of completeness (97.3, 94.7, and and 96.5%; arachnida_odb10) (table S4). These new genome assemblies provide a valuable resource for downstream comparative analyses.

We first reconstructed the chelicerate evolutionary histories using an alignment of 1628 orthologs from 15 sequenced species (table S5)—including the segmented spider, the purse-web spider, and the whip scorpion—to infer the trajectory of spider genome evolution and genomic features (Fig. 1C and table S6). We confirmed that scorpions are the sister group of spiders plus whip scorpions and dated the origin of the monophyletic Arachnopulmonata to the Silurian [~438 million years ago (Ma)] (Fig. 1C), close to previous estimates (*23*). The spinnerets arose ~385 to 380 Ma (*24*) after the divergence of spiders and whip scorpions (~394 Ma) (Fig. 1C). Based on this framework, we used both a chromosome-scale synteny method and a model-based method for WGD inference using gene trees to comprehensively resolve the early arachnid polyploidization events.

In the chromosome-scale synteny approach, we selected ticks as an outgroup because most of its chromosomes are descendants of a single metazoan ancestral linkage group (ALG) (*25*, *26*), and the segmented spider, the purse-web spider, and whip scorpion were selected as ingroups because their chromosome structures are closer to the ancestral state than to true spiders (Araneomorphae) (fig. S4). True spiders’ genomes display a large amount of lineage-specific fusion and fission events, making it difficult to trace the evolutionary history of their genomes (figs. S4 to S8). Using reciprocal best-hit (RBH) analysis with ALGs, we inferred the gene content of 28 ancestral chromosomes (ACs) of chelicerates using tick genes as proxies. A schematic summary of this approach is provided in fig. S9. We first fragmented and reorganized the chromosomes of the three newly sequenced species using a unidirectional BLAST best-hit search, guided by the karyotype of ACs, to identify homologous relationships at the chromosomal level (figs. S10 to S13). We secondly calculated synonymous substitution rates (*K_s_*) between paralogous genes in each genome (Materials and Methods and Fig. 1D) to test the Arachnopulmonata (AR) hypothesis. The results showed that most gene duplicates share a WGD peak of ~10 as anchors (Fig. 1D), suggesting that most of the paralogous genes across the entire genome were created by a single event. Third, based on an almost one-to-one chromosome homology between these species, we selected 457 homologous genes (Materials and Methods and table S7) to construct 19 chromosome-level phylogenies corresponding to the ACs and 14 for the tick scaffolds. The highly supported and well-defined topologies provide further evidence for the presence of the AR event in tetrapulmonates (figs. S14 and S15). In the model-based approach, we examined various scenarios for the sequence of whole-genome duplications by conducting a probabilistic reconciliation of 4166 individual gene trees with species trees using the Bayesian gene tree reconciliation and whole-genome duplication inference by amalgamated likelihood estimation (WHALE) method (Materials and Methods and table S8) (*27*). This analysis provided strong evidence supporting a single-genome duplication event in the Arachnopulmonata stem lineage, with the Bayes factor (BF_Null_vs_WGD_) being less than 10^−3^ (Fig. 1E). Together, our comprehensive analysis suggests that these duplicates arise from an ancient WGD rather than smaller-scale duplication of the Hox clusters (Fig. 1, D to F) and reveals that the early branching lineages of spiders have slow-evolving genomes (Fig. 1, G to I), which offers a window into the early evolution of spider genome features and adaptive traits.

### *Abd-A* gene pair as a driver of spinneret formation

**Fig. 2.**
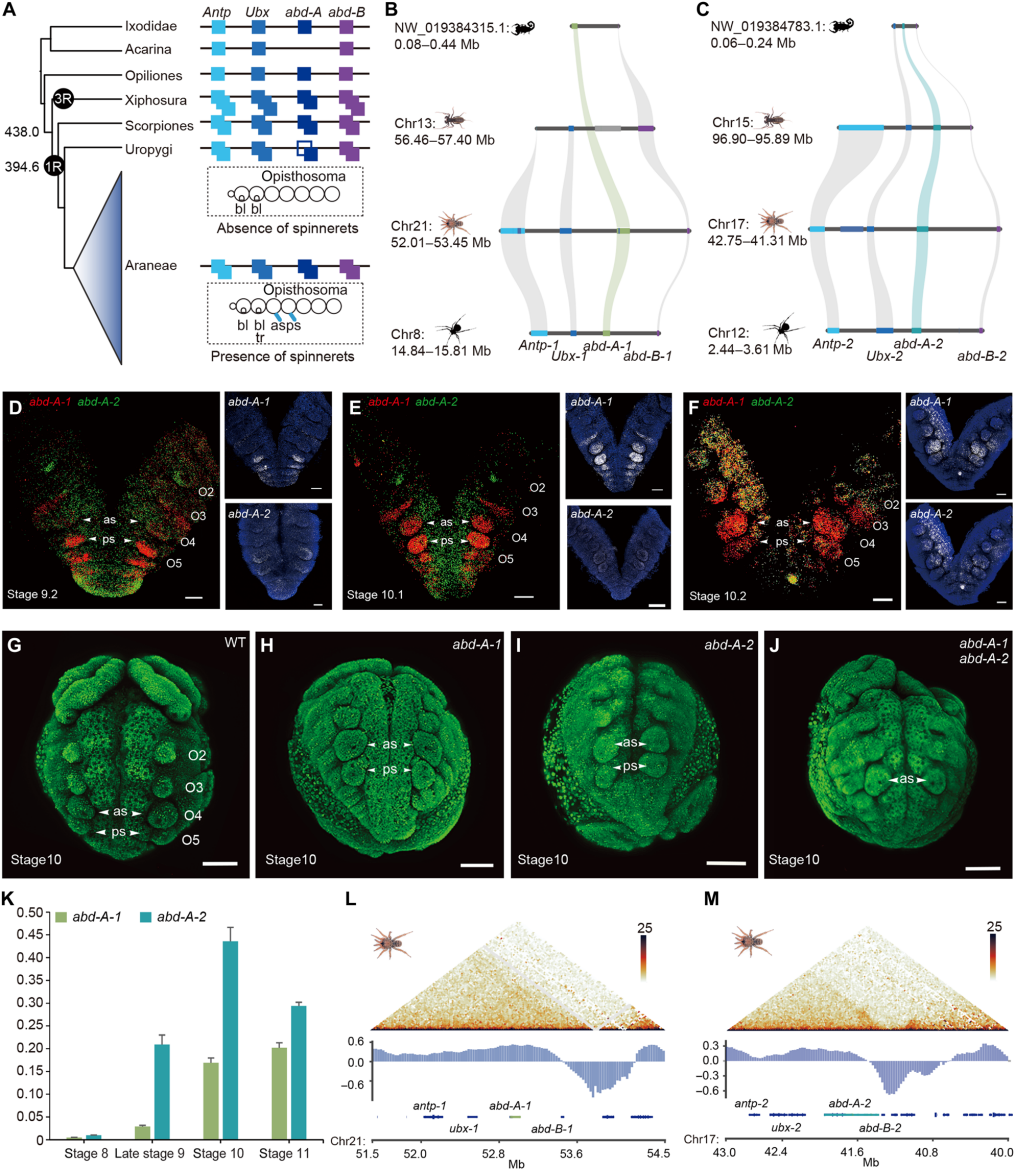
Genome structure-function relationship. (**A**) Gains and losses of opisthosomal Hox gene paralogs in selected arachnid species, including spiders and whip scorpions. (**B** and **C**) Pseudogenization of *abd-A-1* is observed in the whip scorpion genome. (**D** to **F**) HCRs showing that *abd-A-1* (red) and *abd-A-2* (green) are expressed at embryonic stages 9.2 to 10.2 in the developing spinnerets, imaged from the ventral view. Specimens are counterstained with 4′,6-diamidino-2-phenylindole (blue). (**G**) Opisthosoma of wild-type (WT) embryos. (**H**) CRISPR mutagenesis of *abd-A-1* showed no phenotype changes of spinnerets. (**I**) No phenotype changes of spinnerets in *abd-A-2* knockout. (**J**) Double knockout of *abd-A-1* and *abd-A-2* generated an O5 spinneret-loss phenotype. (**K**) Expression of *abd-A-1* and *abd-A-2* at different stages by quantitative reverse transcription PCR. Error bars represent the SDs from eight independent measurements. (**L** and **M**) Hi-C maps from the spider *abd-A* locus at 25-kb resolution, denoting *abd-A-1* and *abd-A-2*, are organized into topologically associating domains (TADs). Scale bars, 100 μm.

To test a previous hypothesis about Hox genes involved in spinneret emergence, we surveyed the Hox genes of seven representative species from our phylogenetic tree. We found that spiders and scorpions retained two *abd-A* after duplication, but *abd-A* of the whip scorpion reverted to a singleton state (Fig. 2, A to C). This contrasts with earlier findings showing that many spider species, as well as whip spiders and a different whip scorpion species, maintain two *abd-A* copies (*13*, *18*, *28*). We noted that *abd-A-1* was the only opisthosomal-specific Hox gene pseudogenized in the whip scorpion *T. vanoorti* (fig. S16). In addition, previous studies associated *abd-A* with morphological diversification of the opisthosoma in spiders (*4*). We examined their expression patterns using in situ hybridization chain reactions (HCRs) in spiders (fig. S17A and table S9). We focused on embryonic stages 9.2 to 10.2 as a readout of the early establishment of the spinnerets. The *abd-A-1* expression starts throughout the limb buds of O5 to the posterior segment addition zone (SAZ) during stage 9.2 (Fig. 2D). The anterior expression border subsequently shifts anteriorly into the posterior part of O3 at stage 10.1 (Fig. 2E). From stage 9.2 to 10.1, *abd-A-2* is expressed throughout the O4 segment and in all posterior segments, with particularly strong expression observed in the limb buds of O4–O5 (Fig. 2, D and E). At stage 10.2, both *abd-A-1* and *abd-A-2* are expressed strongly in the developing limb buds of O4–O5, structures that later develop into the spinnerets (Fig. 2F), consistent with previous studies on the temporal and spatial expression dynamics (*7*, *12*). These findings suggest that *abd-A* copies may contribute to the emergence of the spinnerets.

We next established the embryonic CRISPR-Cas9 gene editing system in spiders (fig. S17B and table S10) (Materials and Methods) to improve our understanding of the functional dynamics of duplicated gene pairs. We first selected a positive control gene, *Ptep-Antp-1*, to test the utility of CRISPR-Cas9 in the spiders (fig. S18 and table S11). Consistent with the previous RNAi phenotype (*7*), mutant embryos exhibited the mosaic phenotype of extra legs in O1 (fig. S18), demonstrating the accuracy and efficiency of gene editing in the common house spider *Parasteatoda tepidariorum*. We subsequently performed single mutagenesis of *Ptep-abd-A-1* and *Ptep-abd-A-2* (tables S10 and 11). We did not observe phenotypic changes of the spinnerets in the mutant embryos (Fig. 2, H and I; and figs. S19 and S20). Mutants of *Ptep-abd-A-1* and *Ptep-abd-A-2* single knockout showed abnormal development of lateral tissue (Fig. 2H and fig. S19) and twisting of the opisthosomal body axis (Fig. 2I and fig. S20) in the SAZ separately. We reasoned that the *abd-A* gene copies evolved functional divergence after duplication and thus retained their ancestral functions together.

Double knockouts of *abd-A* copies in *P. tepidariorum* embryos obtained heterozygous mutants disrupting the formation of spinneret (Fig. 2J and fig. S21). The heterozygous specimens validated by Sanger sequencing exhibited a tracheal defect in the O3 segment and reduced anterior spinnerets at the O4 segment and loss of posterior spinnerets at the O5 segment (Fig. 2J, fig. S21, and table S12). We also obtained a severely mosaic mutant, with O4 and O5 spinneret loss on one side. In addition to the canonical spinneret phenotype, the lateral tissue and body axis of the severely mosaic individual also showed abnormal development (fig. S21). Unfortunately, many embryos die during the early stages of development, and we cannot observe the phenotypes in hatchlings or near-hatchlings. Thus, this double knockout experiment revealed the redundant function of two paralogs of *abd-A* in the identity of both the anterior and posterior spinnerets. To further confirm the role of *abd-A-1* and *abd-A-2* on spinneret formation, we also conducted parental RNAi (pRNAi) in *P. tepidariorum*. Our results of double RNAi with *Ptep-abd-A-1* and *Ptep-abd-A-2* showed spinneret loss at the O5 segment (fig. S22) similar to the CRISPR-Cas9 result. Most embryos from cocoons made after the first one died before stage 9 (fig. S23 and table S13), indicating that pRNAi of *Ptep-abd-A* duplicates disrupt early embryonic development. The phenotypes with high lethality led by RNAi-mediated knockdown of *Ptep-abd-A* duplicates help explain the lack of severely mosaic individuals in the double knockout experiment. Overall, these findings suggest that the gene pair underwent subfunctionalization, with each retaining distinct aspects of the original role. In addition to this division of function, the neofunctionalization of the ancient *abd-A* pair has played a key role in the evolutionary development and diversification of spider spinnerets (Fig. 2J and figs. S21 and S22).

To explain the underlying reason for the evolutionary fates of two gene copies, we explored the expression of *abd-A-1* and *abd-A-2* on spider embryos via quantitative reverse transcription polymerase chain reaction (qRT-PCR). Our results showed a different temporal expression pattern in the two paralogs, indicating their functional differentiation (Fig. 2K). Because evolutionary theory predicts that sequence changes of duplicate genes cause them to evolve sub- or neofunctionalization, we reconstructed a phylogenetic tree of aligned *abd-A* sequences to detect selected signals shared only among spiders using the branch site–likelihood ratio test and site-likelihood ratio test (*29*). The relaxed constraint was observed in both spider *abd-A-1* and spider *abd-A-2* lineages (χ^2^ test, *P* = 0.0200 and *P* = 0.0009, respectively; table S14 and fig. S24A). Moreover, we used DIVERGE v3.0 (*30*) to estimate whether amino acid substitutions between the two *abd-A* paralogs of spiders differed in evolutionary rates (type-I) or amino acid properties (type-II) (fig. S24B). Two type-I functional divergence sites (LOC107450666 P81A and P242T) were identified, which likely indicates the regions of the *abd-A* gene copies of spiders constrained by selection pressure are different. Because the division of function might be due to changes in the regulatory regions of the *abd-A* duplicates, we investigated three-dimensional (3D) chromatin organization in spiders and the whip scorpion using chromatin conformation capture (Hi-C) data. At a scale of tens to hundreds of kilobases, chromosomes are folded into topologically associating domains (TADs) with a median size of 500 kb (fig. S25). TAD features appear to be strongly conserved in spiders and the whip scorpion. For example, the opisthosomal Hox clusters A and B are present within single TADs (Fig. 2, L and M; and fig. S25). These duplicated genes are constrained in the conserved domains, which may limit their divergence, leading to the maintenance of some functional overlap. Thus, together with the functional validation, our results indicate that *abd-A-1* and *abd-A-2* become multifunctional after duplication.

### Elaboration of spider spinnerets from leg homologs

**Fig. 3.**
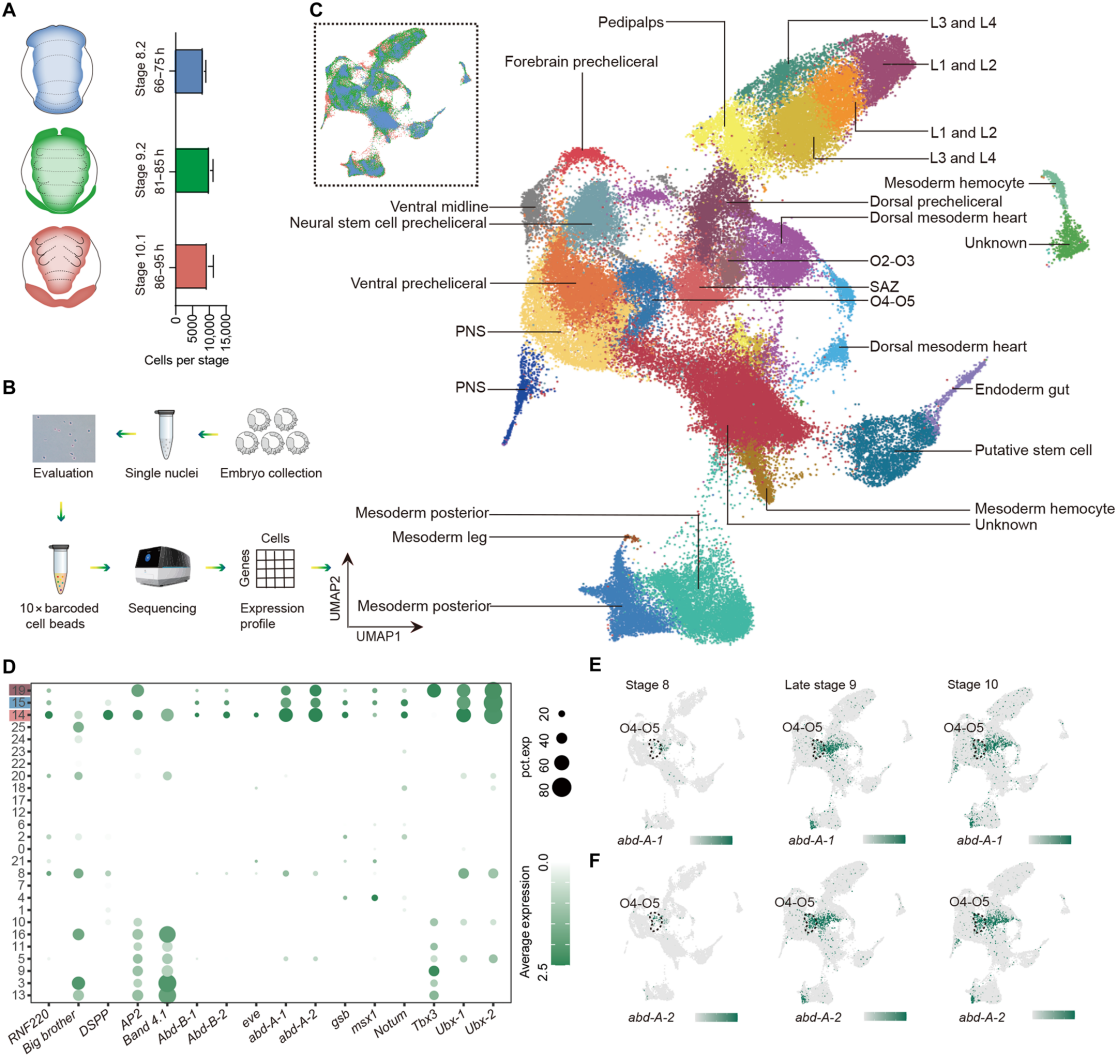
Redundant function of *abd-A* paralogs in spinneret formation. (**A**) Schematic representation of different developmental stages of spider embryos and the number of cells per developmental timepoint. (**B**) Schematic diagram of the single-cell study. (**C**) Uniform manifold approximation and projection (UMAP) of the 27 cell clusters generated by grouping the 76,168 cells obtained from the embryos of eight biological replicates, colored by tissue annotation. Inset colored by developmental time, matching colors in (A). (**D**) Dotplot of markers of SAZ (cluster 14) and primordial tissue of spinnerets (cluster 15) and O2–O3 (cluster 19) in single-cell data of late stage 9. The *y* axis indicates different cell clusters, and they are colored with the representative embryonic zones. (**E** and **F**) UMAP expression plots for *abd-A-1* and *abd-A-2* specific to the opisthosomal segments. SAZ, segment addition zone; PNS, peripheral nervous system.

We further performed single-cell sequencing of the common house spider embryos from stage 8 to stage 10 (Fig. 3, A and B) (*12*, *31*) to explore the gene-regulatory landscape across distinct cell types. After filtering and dimensionality reduction, we obtained 76,168 cells. We used a resolution of 1 and classified cells into 27 major tissues during embryonic development (Fig. 3C and table S15). Cell types were determined using marker genes identified in previous studies and enrichment analyses against the fruit fly single-cell atlas (*32*, *33*). Specifically, we selected five marker genes for the O4–O5 (*abd-A-1*, *abd-A-2*, *notum*, *gsb*, and *msx1*) and three marker genes for O2–O3 (*Tbx3*, *Ubx-1*, and *Ubx-2*) (Fig. 3D) to define the opisthosomal cell clusters. In addition, an increase in the expression level of *abd-A* in the O4–O5 segment from stage 8 to stage 10 confirmed cell-type annotation accuracy (Fig. 3, E and F).

**Fig. 4.**
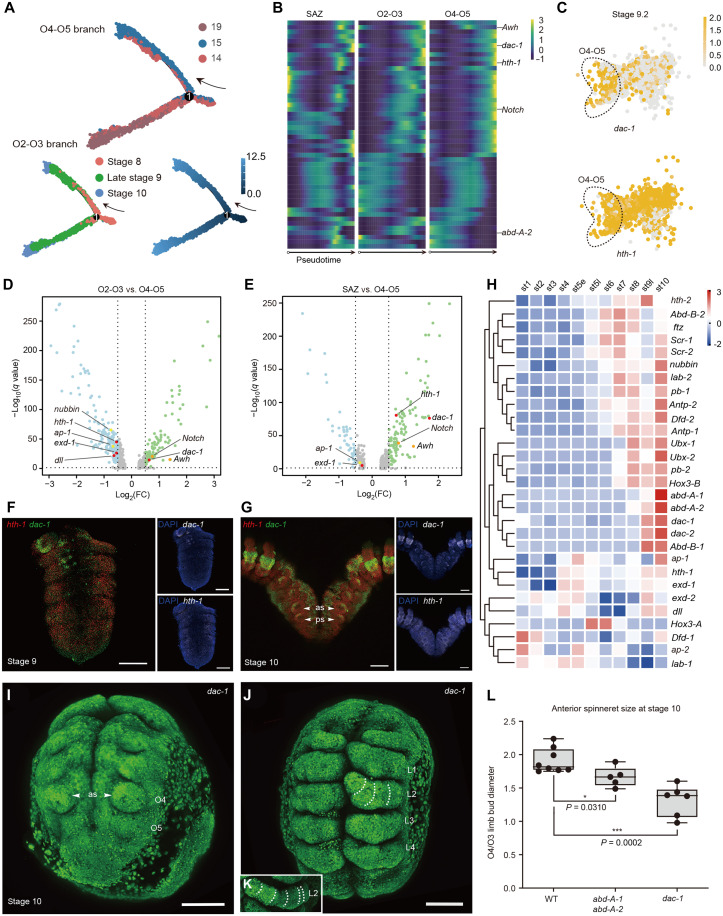
Differently expressed genes in spinneret formation and the function of the leg patterning gene *dac-1*. (**A**) Pseudotime trajectory of opisthosomal cells. Each dot indicates a single cell, color-coded by the cluster as in Fig. 3C. The numbers in black circles indicate branch sites. The black arrows indicate the start of the trajectory. (**B**) Pseudotime heat maps of the top 50 most variable genes enriched in the O4–O5 trajectory branch. Candidate genes listed on the *y* axis play roles in spinneret development. (**C**) Expression pattern of genes involved in leg development (*dac-1* and *hth-1*) in the spider opisthosomal segments. (**D**) Volcano plot of differently expressed genes between O4–O5 and O2–O3 segments at late stage 9. (**E**) Volcano plot of differently expressed genes between O4–O5 cells and SAZ cells at late stage 9. (**F** to **G**) HCRs of embryos at stages 9 to 10 imaged from the ventral side. The leg patterning gene *dac-1* (green) primarily expressed in the developing limb buds of the O2–O5 segment. The leg patterning gene *hth-1* (red) expressed uniformly throughout the opisthosoma, except in the posterior region of the SAZ. Specimens are counterstained with DAPI (blue). (**H**) Heatmap of expression pattern of Hox genes, leg patterning genes, and gill-expressed genes in the embryos at different developmental stages. (**I**) CRISPR mutagenesis of *dac-1* generates a spinneret-loss phenotype. (**J**) Prosoma of a *dac-1*–mutated individual showed the deletion of the medial part of the pedipalps and walking legs (dashed lines). (**K**) Leg 2 of wild-type embryos. (**L**) Comparison of O4/O3 ratio in wild embryos, double knockout of *abd-A-1*/*abd-A-2*, and single knockout of *dac-1* individuals (*P* < 0.05, Student’s *t* test). Error bars represent the SDs from three independent measurements. L, walking leg; O, opisthosomal segment. Scale bars, 100 μm. DAPI, 4′,6-diamidino-2-phenylindole.

To identify genes recruited in the spinneret elaboration process, we first constructed the pseudotemporal trajectory of spider embryo development using opisthosomal single cells spanning stage 8 to stage 10 (Fig. 4A). We found that the initial branch (the posterior growth zone) is composed mostly of cells from stage 8 (Fig. 4A). Stage 9 cells occupy the bifurcation point where the fate of the cells is determined and gradually bifurcated into one of the two branches toward the O2–O3 and O4–O5 fates (Fig. 4A). Cells from stage 10 are located at the tips of specified branches of the pseudotime (Fig. 4A). Among the top 50 differential expression genes across the O4–O5 developmental trajectory, five putative lineage-determining genes (*dac-1*, *hth-1*, *abd-A-2*, *Awh*, and *Notch*) distinguished spinnerets (O4–O5 appendages) from the book lungs (O2–O3 appendages) (Fig. 4B). In addition, the leg patterning gene *dac-1* was significantly up-regulated in the cells of O4–O5, while the *hth-1* was only up-regulated in the expression comparison of O4–O5 and SAZ (Fig. 4, C to E). However, gill-expressed genes did not show the signature of expression differences in the comparisons (Fig. 4, D and E), showing that the development pattern of spinnerets is more comparable to that of the ancestral legs rather than the ancestral gills. We then performed in situ hybridization to confirm our inference (Fig. 4, F and G; and fig. S26). In situ hybridization using a *dac-1* probe revealed the most prominent expression of *dac-1* in the medial part of the walking legs, which aligns with previous studies (*34*, *35*). Our results also show that a clear *dac-1* signal is in the proximal territory of spinneret limb buds, and *dac-1* is expressed later than *abd-A* genes in spinneret morphogenesis (Fig. 4, F and G; and fig. S26). In contrast, *hth-1* is expressed uniformly throughout the opisthosoma, with the exception of the posterior part of the SAZ (Fig. 4, F and G; and fig. S26), a pattern congruent with previous reported spiders (*36*).

We also examined the gene expression profile of spinneret formation during different developmental stages. Combined with the previous dataset of different embryonic transcriptomes from stage 1 to stage 8 (*14*), we sequenced the embryonic RNA sequencing (RNA-seq) data of stage 9.2 and stage 10 in *P. tepidariorum*. After transcriptome quantification and expression clustering, we found that the leg patterning genes *Ptep-hth-1*, *Ptep-dac-1*, and *Ptep-dac-2* showed similar expression patterns to *Ptep-abd-A* duplicates (Fig. 4H and fig. S27). Our results suggest that leg patterning genes may be essential for spinneret formation.

To validate the effects of tool kit genes on spinneret formation, we conducted the mutagenesis of the *hth-1* and *dac-1* genes. *Ptep-dac-1* mutants and *Ptep-dac-1* RNAi embryos generated a spinneret-loss phenotype at the O5 segment, a comparable scale of reduced anterior spinnerets at the O4 segment, and defective limb buds at the O2–O3 (Fig. 4I and figs. S28 and S29). *Ptep-dac-1* mutagenesis and *Ptep-dac-1* RNAi embryos also lacked the medial part of the pedipalps and walking legs (dashed lines) (Fig. 4, J and K; and figs. S28 and S29), where *Ptep-dac-1* is strongly expressed (*7*). These results indicated that *dac-1* promotes the development of spinneret primordia. Because of the high lethality of RNAi embryos with strong phenotypic defects (figs. S29 and S30 and tables S10 and S16), we could not collect sufficient individuals at the stage of postembryo. We found that spinnerets at O4 and O5 still existed while the buds of the book lungs and tracheae at O2 and O3 were enlarged (fig. S31) in the *Ptep-hth-1* mutants (*31*). Limited leg-related genes occur at the intersection of the temporal and spatial difference of spinneret formation (tables S17 to S19), leaving an open question regarding the leg-origin hypothesis (fig. S32).

### The gene network underlying spinneret development

**Fig. 5.**
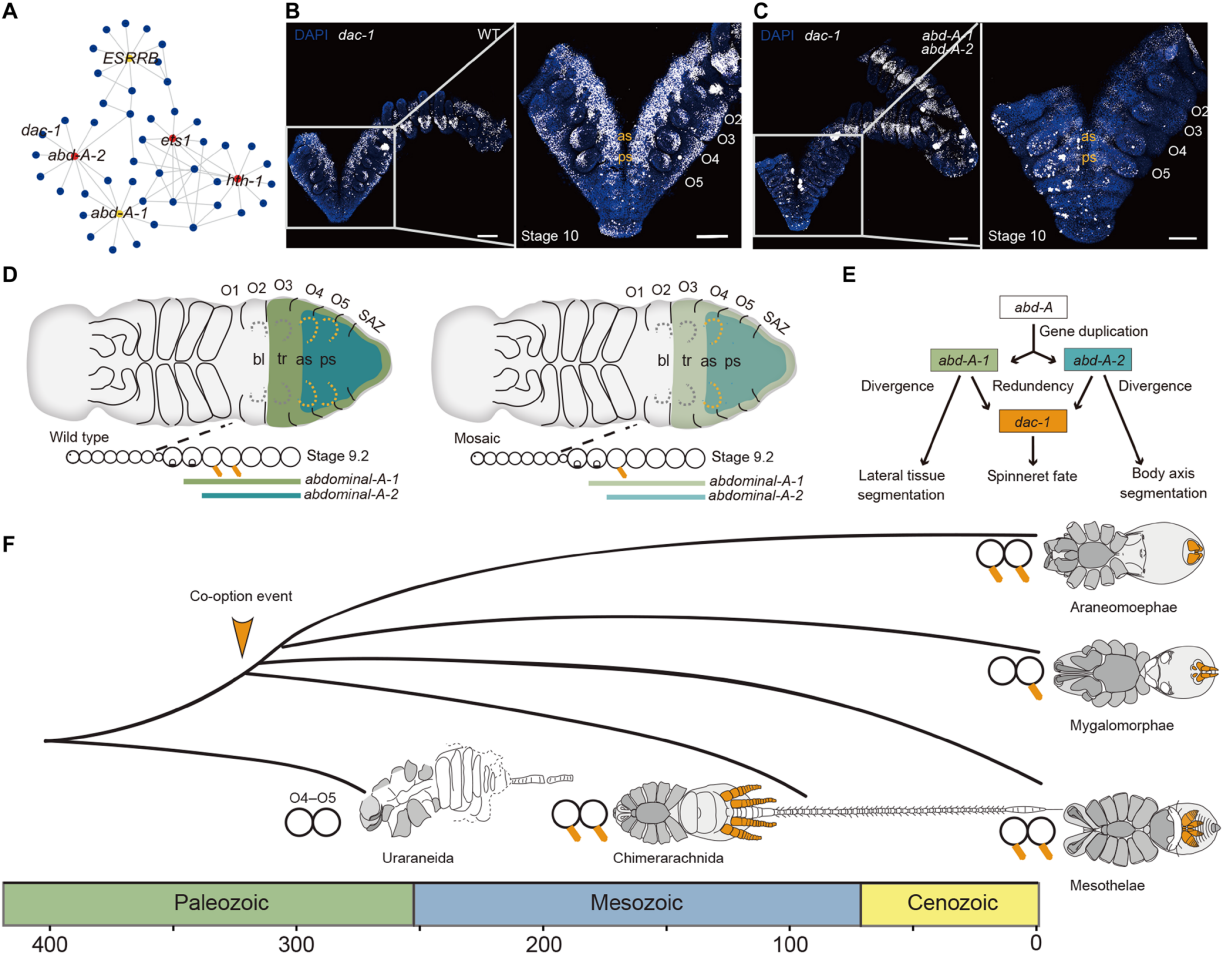
Genetic mechanism of spinneret formation and the evolutionary history of spinnerets. (**A**) Transcription networks of the top 5% hub TFs regulating spinnerets. Target genes for each TF are listed in table S20. (**B**) HCRs showing wild-type expression of *dac-1* (white) at embryonic stage 10.1 in the developing limb buds from O2 to O5. (**C**) Weak phenocopies from *abd-A-1* and *abd-A-2* RNAi showing diminished *dac-1* (white) expression at embryonic stage 10.1 in the developing limb buds from O2 to O5. Specimens are counterstained with DAPI (blue). Scale bars, 100 μm. (**D**) Expression and regulation of Hox genes and related genes in *P. tepidariorum* spinneret formation. (**E**) Schematic of the inferred *abd-A* gene pair and *dac-1* interactions, which indicate the new and redundant function of *abd-A* gene copies in the formation of spinnerets after an ancient WGD. The leg-patterning gene *dac-1* is co-opted into the *abd-A* gene network. (**F**) Evolutionary history of spiders and relatives and spinneret phenotypes (orange).

To recontextualize single-cell data in a system-level framework, we formed coexpression networks to identify spinneret-associated gene modules based on the single-cell weighted gene correlation network analysis (scWGCNA) approach (*37*). In particular, our scWGCNA analysis of opisthosomal cell clusters (*38*) identified coexpression module 21 as being notably associated with spinneret development (Fig. 5A and fig. S33). Among 190 gene members in module 21, *abd-A-1*, *abd-A-2*, and *dac-1* are known to be involved in spinneret formation. We also constructed regulation networks for the transcription factors (TFs) of module 21 using the GENIE3 package in R (*39*) and plotted the top 10 target genes of TFs with Cytoscape (Fig. 5A) (*40*). Target genes for each TF are listed in table S20.

We also assayed the expression patterns of *dac-1* in the *abd-A* RNAi embryos. RNAi-mediated knockdown of the spider *abd-A* ortholog and paralog resulted in a diminution of *dac-1* expression in the O4–O5 spinneret primordia (Fig. 5, B and C; and fig. S34), whereas the expression patterns of *dac-1* in the legs of *abd-A* RNAi embryos were comparable to wild-type individuals. Our findings demonstrate that leg patterning genes, such as *dac-1*, were recruited into the *abd-A-1* and *abd-A-2* regulatory network to guide spinneret elaboration in the initial stage of development (Fig. 5E). Given that both duplicated Hox gene pairs and leg patterning genes contributed to spinneret development, our findings support the idea that regulatory genes and networks can evolve modularly and independently. This flexibility likely facilitates the emergence and diversification of complex traits.

## DISCUSSION

This work provides a detailed understanding of the spinneret’s biology by combining multiomic and functional approaches. We found that both copies of *abd-A* contribute to the emergence of spinnerets on the O4–O5 segments, which have filled knowledge gaps regarding the early evolution of arthropod-specific traits. Unlike the *abd-A* gene copies in spiders, which have retained their ancestral function while also evolving novel and overlapping roles in spinneret identity, Hox-duplicated gene pairs in other species—such as flies (*9*, *10*), butterflies, and moths (*11*)—have lost their homeotic function and acquired new functions during development. In addition, we explored the evolution of spinnerets from ancestral limbs at single-cell resolution and proposed that the recruitment of the leg patterning gene *dac-1* by the *abd-A* gene pair plays a key role in the transformation of leg serial homologs into spinnerets. These findings suggest that long-term natural selection may drive the rewiring of duplicated gene networks, resulting in the evolution of novel functions and increased biological complexity.

WGD represents a rare yet notable event in the evolutionary history of animals. These events are believed crucial in driving evolutionary novelties and adaptive radiation, such as the vertebrate and gnathostome novelties produced by 1R and 2R events (*41*, *42*). Nonetheless, direct genomic evidence linking WGD to the evolution and diversification of animals, particularly arthropods, remains limited (*22*). The argument about an Arachnopulmonata-specific WGD is largely based on fast-evolving spiders’ genomes (*12*, *19*). To address this, we sequenced chromosome-scale genomes from three karyotypically conserved species, allowing us to reconstruct the evolutionary history of the arachnid genome through macrosynteny analysis. Our findings further support the AR scenario of an ancient WGD event, followed by substantial reductions in duplicated redundancy and chromosome number. While WGD is typically regarded as rare in animals with chromosomal sex determination due to its disruptive effects (*43*), our study provides empirical evidence linking an ancient WGD event to the emergence of evolutionary novelties (fig. S35). This challenges us to reconsider the frequency and significance of ancient WGD events in the animal kingdom.

At a macroevolutionary scale, we proposed that a co-option event associated with spinnerets might have occurred before the divergence of the Araneae from its extinct sister lineage Chimerarachnida (*44*), which is essential for adapting to a terrestrial lifestyle. Spiders from the Mesothelae, Mygalomorphae, and Araneomorphae evolved diverse multisegmented spinneret types (*45*) via adaptive diversification into a variety of ecological niches (Fig. 5F). Studies in mygalomorph spiders have shown that the limb buds of the posterior spinnerets express both *abd-A-1* and *abd-A-2*. In contrast, the anterior spinnerets, which lack *abd-A-2* expression, regress, and are absent in later developmental stages (*4*). In addition, the limb buds of the rudimentary anterior spinnerets do not express *dac-1* (*3*). These expression patterns suggest that the development of functional spinnerets relies on the coordinated expression of both *abd-A* gene pair and *dac-1*. Our study, along with observed differences in posterior Hox genes and *dac-1* expression between Mygalomorphae and Araneomorphae, supports the notion that both *abd-A* gene pair and *dac-1* play critical roles in spinneret formation. In addition to duplicated Hox genes and limb-patterning genes, our single-cell transcriptomic data uncover previously underappreciated insights into spinneret development. Notably, we found that *Notch* and *Awh* are consistently and highly expressed in spinneret limb buds (Fig. 4, B, D, and E). When considered alongside prior studies (*46*), this expression pattern suggests that *Notch* may function downstream of *dac-1* to regulate spinneret segmentation and growth. Here, we show that the spinneret likely originated through the cooption of parts of an ancestral appendage GRN to novel locations on the abdomen. From this perspective, our future efforts will be directed toward understanding the downstream molecular regulators of *dac-1* and the species-specific GRNs in different spinneret types.

Beyond their evolutionary significance, spinnerets have become a central focus in synthetic biology due to their role in spider silk production, which has immense biotechnological potential. Spider silk’s remarkable mechanical properties—strength, elasticity, and lightness—make it ideal for diverse applications, from biodegradable medical sutures to advanced materials for aerospace and construction. However, large-scale production and the precise in vitro reconstruction of silk remain challenging due to the intricate and highly specialized process by which spiders synthesize, assemble, and extrude silk fibers (*47*). Understanding the genetic basis of spinneret provides critical insights for advancing synthetic spider silk (*48*). Our study offers comprehensive support for this goal by identifying genes involved in the development and evolution of spinnerets, such as *abd-A-1* and *abd-A-2*, which contribute to the emergence of novel appendages, and *dac-1*, which aids in the elaboration of spider spinnerets from leg homologs. Techniques such as gene editing can fine-tune these genes to potentially improve large-scale spider silk production. Moreover, understanding the evolutionary strategies of spinneret diversification will help guide the design of synthetic spider silk for various applications using technologies such as biomimetic design, microfluidics, and bioprinting. These innovations hold great promise for overcoming the limitations of traditional materials and offer a model for developing high-performance materials for fields such as medicine, textiles, and defense.

## MATERIALS AND METHODS

### Genome assembly and annotation for *L. beijing, T. vanoorti*, and *Atypus* sp.

To obtain genome assembly at the chromosome level of *L. beijing* and *Atypus* sp., we used Hi-C–based proximity-guided assembly (*49*) to optimize the published genome of this species (*50*). Samples of *L. beijing* were collected from Purple Bamboo Park, Haidian district, Beijing, China. Hi-C libraries were prepared following a published protocol with minor modifications (*51*). Samples of the purse-web spider (*Atypus* sp.) were collected from the bamboo forest Tongji, Chengdu, Sichuan, China. For cross-linking, samples were fixed with 1% formaldehyde. The cross-linked DNA was digested with Mbo I restriction endonuclease and marked with biotin-14-deoxycytidine triphosphate (dCTP) to remove nonligated DNA fragments. The ligated DNA was extracted with the QIAamp DNA Mini Kit (QIAGEN). The purified DNA was then sheared to ~350–base pair (bp) fragments and followed by a standard Illumina library preparation protocol (*52*). Hi-C sequencing was conducted on the Illumina HiSeq platform with paired-end (PE) 150 bp. We then filtered the raw reads to remove low-quality reads and adapters, yielding 204.89 Gb of clean data for *L. beijing* and 135.89 Gb of clean data for *A*. sp., separately. Hi-C library sequencing data were mapped to the scaffold level genome of *L. beijing* using Juicer v1.6.2 (*53**–**55*).

Samples of *T. vanoorti* were collected from Jianfengling National Forest Park, Hainan, China. Double-stranded DNA template capped by hairpin loops at both ends (SMRTbell) libraries of *T. vanoorti* were constructed according to the standard protocol of PacBio using 15 kb of preparation solution (PacBio, CA, USA). The high-fidelity (HiFi) libraries were sequenced on three SMRT cells on the PacBio Sequel II system in circular consensus sequencing mode at Novogene Technology Co. and generated 49.75 Gb of HiFi data total. PacBio reads were first assembled using two de novo assemblers: hifiasm v0.19.7 (*56*) and wtdbg2 v2.5 (*57*). Two versions of contigs were polished with Racon v1.4.17 (https://github.com/isovic/racon) and NextPolish v1.4.0 (*58*). Contig level genome completeness assessment was performed using BUSCO v5.2.2 (*59*), and the best assembly was selected for downstream analysis. For Hi-C data acquisition, we used the same method as mentioned above and obtained a 263.95 Gb of library. The Hi-C library was mapped to the contig results of a de novo assembly using the above method, and we obtained a genome assembly at the chromosome level.

The RepeatModeler v2.0.2 (*60*) and RepeatMasker v4.1.2-p1 (*61*) pipelines were used to annotate repetitive sequences in the genome. All annotation strategies are based on transcriptome data and were carried out through the TransDecoder pipelines (v5.5.0; https://github.com/TransDecoder).

### Ethics statement

Sample collection was carried out in compliance with the approved guidelines of the Good Experimental Practices adopted by the Institute of Zoology, Chinese Academy of Sciences (CAS). All experimental procedures and sample collections were performed under the supervision of the Committee for Animal Experiments of the Institute of Zoology, CAS.

### Orthologous gene identification and phylogenetic analysis

We used OrthoFinder v2.5.4 (*62*) to analyze the annotation information of species in this study (table S5). In the pipeline, Mafft v7.453 (*63*) was used to perform multiple sequence alignment, blastp v2.12.0+ (*64*) was used to perform sequence searches, and the phylogenetic tree was constructed using FastTree v2.1.11 (*65*). To obtain the divergence times at each node, mcmctree in the Phylogenetic Analysis by Maximum Likelihood (PAML v4.9j) toolkit was used to estimate the divergence time of the phylogenetic tree from the OrthoFinder pipeline results. The calibration points are from fossil specimens (*66*, *67*) and TimeTree (www.timetree.org/). Subsequently, we used Computational Analysis of Family Evolution (CAFE v4.2) (*68*) under the default parameters to analyze the gene family expansions and contractions.

### Tests of WGD hypotheses on the chelicerate phylogeny

We analyzed the gene set of the Arachnopulmonata species, including ticks, which have not been associated with WGD (*69*). When calculating the synonymous substitution rate (Ks) of genes, to remove interference as much as possible, paralogous gene pairs belonging to the same chromosome are excluded, and only *K_s_* between paralogous homologs on different chromosomes are calculated. Comparison of *K_s_* between bidirectional best-hit (RBH) gene pairs (*e*-value threshold of 1 × 10^−3^) within each Arachnopulmonata species revealed two prominent peaks in the *K_s_* distribution. In contrast, ticks exhibited a single peak (Fig. 1D), indicating a duplication event may occur in Arachnopulmonata species.

Using RBH analysis with ALGs, we reconstructed the gene content of 28 ACs using *Ixodes scapularis* (tick) genes as proxies. Homology between Arachnopulmonata species and the inferred ACs was established indirectly based on their shared gene relationships with the tick genome. Because the ACs did not undergo genome duplication, we used genes on the ACs as probes to perform a unidirectional best Basic Local Alignment Search Tool-Protein (BLASTP) search in each Arachnopulmonata species (*T. vanoorti*, *L. beijing*, and *Atypus* sp.). This approach calibrated the ACs by identifying corresponding genes in each Arachnopulmonata species. Based on these results, for each Arachnopulmonata species, if several genes corresponded to the same ACs in succession, we isolated the chromosome segments containing them and grouped all segments corresponding to the same ACs into a cluster. We then randomly connected fragments from each cluster to form reorganized chromosomes and validated our clustering results with ACs as a reference, visualizing the chromosome reconstruction as a scatter plot (figs. S6 to S9) (*26*). For each cluster, we iterated through every chromosome segment and searched for RBH genes corresponding to the ACs of that cluster. This process identified the homologs of ancestral genes across different segments, ensuring that homologs from distinct segments were considered, thereby avoiding interference from small-scale duplications within segments. Ancestral genes with homologs on exactly two segments were directly selected, along with their homologs, for further analysis. If an ancestral gene had homologs on more than two segments, then the homologs from the two longest segments were chosen. This process was conducted separately for different Arachnopulmonata species, ultimately identifying 457 ancestral genes, each with two homologs per Arachnopulmonata species. With homologous gene sets, we used Mafft v7.453 (*63*) and FASconCAT-G v1.05.1 (*70*) to perform multiple sequence alignment. We used ModelFinder to find the best-fitting evolutionary model. Phylogenetic trees were generated using IQ-TREE2 (*71*) for multiple sequence alignments containing at least 5000 sites. Seventeen of the 19 homologous gene sets strongly supported a genome duplication event. Our chromosome level phylogeny supports the hypothesis that the whip scorpion, segmented spider, and purse-web spider share a common genome duplication event.

We used OrthoFinder v2.5.4 (*62*) to build orthologous gene families on a reduced chelicerate species tree (table S5). Mafft v7.453 (*63*) was used to perform multiple sequence alignment, blastp v2.12.0+ (*64*) was used to perform sequence searches, and the phylogenetic tree was constructed using FastTree v2.1.11 (*65*). We summarized clade conditional distribution (CCD) from bootstrapped trees using the ALEobserve tool from the ALE software. We used WHALE software v2.1.036 (*27*) to test the AR hypothesis, an Arachnopulmonata-specific duplication, on the dated species trees and CCD data. A variable rate DLWGD WHALE model was implemented for the inference of the significance and corresponding retention rates of the assumed WGD events under Bayesian inference.

### Hi-C analysis

The visualization of normalized Hi-C matrices and other values described below—such as insulation scores, TAD boundaries, aggregate TAD, Pearson’s correlation matrices, and eigenvectors—were calculated and visualized using FAN-C 0.9.26-beta (*72*). Using FAN-C, we systematically identify differences at all scales of the chromatin organization hierarchy between spiders and the whip scorpion.

### Quantitative RT-PCR

According to the previous in situ hybridization results of *abd-A-1*, *abd-A-2, dac-1*, and *hth-1* (*12*), we collected the spider embryos of four stages, stage 8, stage 9.2, stage 10, and stage11 to cover the process of spinneret formation. Total RNA was extracted and diluted to 200 ng/μl. The genomic DNA was removed, and the cDNA was then synthesized using the HiScript III 1st Strand cDNA Synthesis Kit (Vazyme). The qRT-PCR was performed by TB Green Premix Ex Taq II (Takara), and the reactions were run on LightCycler 96 System (Roche). The primers were synthesized in Tsingke Biotechnology Company, and the primer sequence can be found in table S11.

### Selection pressure and functional divergence analysis

Sequences of *abd-A* were identified in genomes of *Limulus polyphemus*, *Centruroides sculpturatus*, *P. tepidariorum*, *Stegodyphus dumicola*, *Strigamia maritima*, *Bombyx mori*, *Parhyale hawaiensis* using blastp (*e*-value threshold of 1× 10^−20^). The alignment details are provided in the Supplementary Materials. To infer the selection pressure among the *abd-A* sequences, we used codeml in the PAML package to run three models: the branch-site selection model (model A), the branch-site neutral model (model A1), and a nearly neutral sites model (M1a) (*29*). Foregrounds were labeled as haploid ancestor *abd-A*, polyploid ancestor *abd-A-1*, polyploid ancestor *abd-A-2*, spider *abd-A-1*, spider *abd-A-2*, respectively. In the null model A1, foreground sites are neutral or under purifying selection, while model A allows sites to be under positive selection. Likelihood ratio tests (LRTs) between model A and its null model A1 were performed to assign significance of positive selection, while LRTs between model A1 and M1a were used to detect the signature of relaxation of constraint on selected foregrounds. *P* values of LRTs were calculated using a chi-square distribution with 1 degree of freedom.

Two types of functional divergence between *abd-A-1* and *abd-A-2* genes were calculated by DIVERGE3.0. If divergence coefficients (θI and θII) are significantly greater than 0, then it implies that the selection constraints (Type-I) or the physicochemical properties (Type-II) of the particular amino acid residues were changed significantly between the two paralogs of *abd-A* genes. We deployed 1000 bootstraps to obtain the posterior probability (*Q_k_*) at each site. The higher the *Q_k_* value is, the higher the probability of functional divergence of type I or type II is. In this study, the cutoff value of *Q_k_* is set to 0.8.

### Pseudogenization

Using all protein sequences of the common house spider, segmented spider, and whip scorpion as references, we predicted pseudogene sequences and counted the number of disabling mutations (premature stop or frameshift mutations) in the whip scorpion (*T. vanoorti*) genome through the PseudogenePipeline calling tblastn with default parameters (*73*).

### Animal culture and genome editing in spiders

Our *P. tepidariorum* were collected in Beijing and were maintained at 24° to 26°C with light/darkness cycles of 16/8 hours and ~70% relative humidity. The animals were kept separate in plastic containers (5.5 cm by 5.5 cm by 4.5 cm). Spiders were fed with *Drosophila melanogaster* flies. After mating, several cocoons were produced by the females at regular intervals. Each cocoon contains about 150 eggs. Females bearing a cocoon with eggs were paralyzed with CO_2_ and revived by blowing fresh air after removing the cocoon from the female with forceps. The cocoon was opened with scissors, and eggs were gently collected with small brushes. *P. tepidariorum* spiders have soft eggs with low internal pressure, so long and narrow glass needles are suitable for injection. Glass capillaries (Sutter BF100-50-10) were pulled using a P-97 Flaming/Brown type micropipette puller to make injection needles. The end of each injection needle was broken off with dissecting scissors just before use.

We designed single-guide RNA (sgRNA) using the program sgRNAcas9 (*74*) against the *P. tepidariorum* genome to avoid potential off-target effect. sgRNA target sites were selected from sequences corresponding to GN19NGG on the sense or antisense strand of the DNA. The CRISPR forward primer contains the T7 polymerase binding site, target site GN19, and a region complementary to the CRISPR reverse primer, and the sgRNA was synthesized by PCR-based strategies. After in vitro transcription and purification of sgRNA, the efficiency of the sgRNA-Cas9 mixture was verified via an in vitro cleavage assay, following the previous protocol for CRISPR-Cas9 genome editing (*75*). In the *P. tepidariorum* eggs, cellularization does not occur until there are ~16 nuclei (around 9 hours) (*31*). Therefore, we collected the two to eight nuclei eggs (around 3 to 7 hours) after oviposition. The eggs were aligned on the plate sieve in a petri dish and covered with halocarbon oil 700 (Sigma-Aldrich). We used a mixture containing sgRNA (single-guide RNA) and Cas9 protein (500 to 1000 ng/μl) to inject eggs. After injection, the petri dish was placed at 24° to 26°C with ~70% relative humidity. The stained embryos were photographed under a stereomicroscope (Nikon SMZ18) and with light sheet microscopy (Zeiss LightSheet Z.1).

### Genotyping

To validate that genome editing is occurring as expected at the appropriate locus, we extracted genomic DNA from injected embryos. Primers for genotyping were designed outside the sgRNA target sites to cover all possible mutant sequences. Genotyping primers can be found in table S11. We amplified the sequence of target regions using polymerase chain reaction (PCR) and carried out bidirectional Sanger sequencing of PCR amplicons to identify mutants. PCR amplicons were then cloned and sequenced using a pEASY-T1 Cloning Vector kit (TransGen Biotech). Cloning primers are listed in table S11.

### RNA interference

Parental RNAi followed previous protocols for double-stranded RNA (dsRNA) injection in virgin females of *P. tepidariorum* (*8*). *Ptep-abdA-1* and *Ptep-abdA-2* dsRNA were produced separately and then combined. Each female was injected four times every second day using 4 μg of each gene. Virgin females were injected with dsRNA of a 1240-bp cloned fragment for *Ptep-abdA-1* and a 1115-bp cloned fragment for *Ptep-abdA-2* (fig. S22). For *Ptep-dac-1*, RNAi was performed with 20 μg of dsRNA of a 944-bp fragment (fig. S29) delivered four times over 8 days to 12 virgin females, with 6 surviving to lay a second cocoon. Control animals were injected with dsGFP and kept under the same condition as dsRNA-injected animals. All females were mated to untreated males and were fed approximately every other day after the last injection. Cocoons were collected and analyzed from the first clutch, approximately one per week. The phenotypes of offspring for all cocoons were recorded after hatching. Hatchlings were scored in three classes: (i) Wild-type, (ii) RNAi phenotype, and (iii) dead. The validation of the knockdown was assessed using in situ hybridization.

### Processing of samples for scRNA-seq

We selected three embryonic stages for single-cell sequencing, including stage 8, stage 9.2, and stage 10, based on the previously described staging (*31*). Embryos were sent to Beijing Capital Bio Technology Co. Ltd. for dissociation and library preparation. Single-cell RNA-seq (scRNA-seq) libraries were generated using the Single Cell 3’ Library and Gel Bead Kit V3.1, and sequencing was performed on an Illumina NovaSeq 6000 platform, achieving a minimum depth of 100,000 reads per cell with a 150-bp paired-end reading strategy.

Sequencing data were generated using the 10x Genomics Chromium platform. After quality control, reads were aligned to the *P. tepidariorum* reference genome (*76*) using STAR v2.6.1a with default parameters. Gene expression matrices were produced for each sample using the Cell Ranger pipeline (v4.0.0). We retained cells expressing between 300 and 9000 genes and with mitochondrial gene expression below 5%. Eight samples representing the three developmental stages were then integrated into a single dataset using the FindIntegrationAnchors and IntegrateData functions. The combined dataset was scaled and subjected to dimensionality reduction using principal components (PC) analysis with the first 50 components. The first 40 PCs were used to construct a shared nearest neighbor network, and clusters were identified using the Louvain algorithm that was implemented in the Seurat FindClusters function. Last, the resulting clusters were visualized using the uniform manifold approximation and projection (UMAP) method by the RunUMAP function (parameters reduction = “pca,” dims = 1:40).

### Bioinformatic processing of raw sequencing data and independent cell-type clustering analysis

To determine an appropriate resolution for clustering the 76,168 cells, we tested a range of resolution values (0.5 to 2.0, in increments of 0.5) and manually evaluated the resulting cluster structures. We selected the clustering resolutions that most effectively distinguished differentiating germ layers, tissues, and organs into discrete clusters. Key cell types were identified on the basis of established marker genes previously reported by Leite *et al.* (*33*), with established marker genes as listed in table S15. We further defined the O2–O3, O4–O5 (spinneret), and SAZ cells from opisthosomal segments using both published marker genes and posterior Hox gene expression patterns and compared the expression difference of these three cell types using Seurat v4 (*77*). This led us to finally choose the version generated by a resolution of 1 for all stages. Monocle3 (v.1.3.1) was used to predict single-cell developmental trajectories and display single-cell gene expression kinetics through the opisthosomal clusters, including O2–O3, O4–O5, and SAZ. We used the following workflow and parameters: estimate_size_factors(), detect_genes(min_expr = 0.1), preprocess_cds(num_dim = 50), align_cds(residual_model_formula_str = “~log10(nUMI)”), reduce_dimension(preprocess_method = “Aligned”), cluster_cells(), fit_models(model_formula_str = “~splines::ns(pseudotime,df = 3)”), and lastly model_predictions(). We performed scWGCNA by starting from the opisthosomal clusters following the proposed workflow using the default parameters. The regulatory network was generated using GENIE3 (*39*) by identifying the regulators of the genes of the network in single cells. We then used cytoHubba to score hub objects (*78*). The top 10 target genes of hub genes in the regulatory network were visualized using Cytoscape v.3.7.2. (*40*).

### RNA-seq and expression analysis of embryos

To explore the expression profile of developmental embryos, especially the spinneret identity at stage 9.2, we sequenced the RNA-seq data of stage 9.2 and stage 10 and combined them with the previous dataset of different embryonic transcriptomes from stage 1 to stage 8 (*14*). After transcriptome quantification, we generated the final gene expression profile across developmental stages. To analyze temporal expression patterns, we applied the Mfuzz package (*79*), which performs soft clustering based on normalized read counts. This approach allowed us to group genes with similar expression dynamics throughout development. Our analysis focused on clusters containing genes whose expression peaked at stages 9.2 to 10, corresponding to key transitions in embryogenesis. Genes with more than 25% missing expression values were excluded. For the remaining genes, missing values were replaced by the average expression value of each gene. Default parameters were used in filtering and standardization. The expression data of Hox genes, leg patterning genes, and gill-expressed genes were subsequently visualized as a heatmap in the R package ClusterGVis.

## Supplementary Materials

### The PDF file includes:

Supplementary Text Figs. S1 to S35 Legends for tables S1 to S20 References
