# Supplementary material for: An ancient genome duplication event drives the development and evolution of spinnerets in spiders
Source: Sci Adv. 2026 Jan 14;12(3):eadw2173. doi: 10.1126/sciadv.adw2173 (PMC12802834; doi:10.1126/sciadv.adw2173)
Supplement: Supplementary file 1 — Supplementary Text Figs. S1 to S35 Legends for tables S1 to S20 References [file sciadv.adw2173_sm.pdf]

Science Advances
AAAS

Supplementary Materials for

An ancient genome duplication event drives the development and evolution of spinnerets in spiders

Fengyuan Li *et al.*

Corresponding author: Wei Zhang, weizhangvv@pku.edu.cn; Shuqiang Li, lisq@ioz.ac.cn

Sci. Adv. **12**, eadw2173 (2026)
DOI: 10.1126/sciadv.adw2173

The PDF file includes:

Supplementary Text
Figs. S1 to S35
Legends for tables S1 to S20
References

Other Supplementary Material for this manuscript includes the following:

Tables S1 to S20

Supplementary Notes

Selection Pressure and Functional Divergence Analysis

We used stringent alignment procedures to ensure accuracy. Eleven sequences were aligned with PRANK v150803 (*80*) using default parameters, and columns that were not conserved across Arthropoda were removed manually. The residues with low scores (≤ 0.93) were masked using Guidance v2.02 (*81*). Columns with more than 10% gaps or below the similarity threshold of 0.001 were trimmed by trimAL v1.4.1 (*82*). A maximum-likelihood phylogenetic tree was reconstructed using RAxML v8.2.9 (*83*), with a GTRGAMMA model and 500 rapid bootstrap iterations.

Fluorescent *in Situ* Hybridization

After fixation, the devitellinized embryos were stored at -20°C until further use. Custom software (*84*) was employed to design HCR probe sequences, ensuring minimal risk of off-target binding. Detailed information on probe design and sequences can be found in the Supplementary Table 9 online. Fluorescent in situ hybridization followed a modified version of the Molecular Instruments (Los Angeles, CA, USA) HCR v.3 protocol (*85*). Day 1: Devitellinized embryos were gradually rehydrated in PBST, post-fixed in 4% paraformaldehyde for 20 minutes, and rinsed with PBST. They were then treated with a detergent solution at room temperature for 30 minutes to permeabilize the cell membrane. Each sample was then prehybridized by adding 100 µL of hybridization buffer and incubating at 37 °C for 30 minutes in a PCR tube. Hybridization was performed by replacing the buffer with 0.4 pmol of the probe set, followed by overnight incubation at 37 °C. All Day 1 procedures were conducted on a shaker set to 25 rpm. Day 2: Excess probes were sequentially rinsed in probe wash buffer to which 5× saline sodium citrate buffer with Tween (SSCT) had been added to final concentrations (vol/vol) of 0% (5 times), 50%, and then 100% for 30 min each at 37 °C. Samples were rinsed before preamplification with 5× SSCT for 10 min and incubated in amplification buffer for 30 min at room temperature. After removing the preamplification buffer, 100 µL of hairpin solution, prepared according to the manufacturer's instructions and snap-cooled, was added to each sample and incubated in a dark chamber at room temperature overnight. Day 3: Excess hairpin solution was removed through three rinses with 5× SSCT for 30 min, followed by a final rinse for 10 min. Finally, imaging was performed using a Leica Stellaris 5 confocal microscope, and images were processed using Leica Application Suite X 4.5.0.25531. Yolk particles were manually removed using fine forceps during re-fixation in 4% paraformaldehyde at the start of the HCR protocol, and again at the end, just before the glycerol step, while embryos were in 5× SSCT.

Embryo Fixation and Staining after Genome Editing

Currently, injected spider embryos must be reared in oil, and there has been no successful rearing experience after removing them from the oil, as indicated in recent studies (*86*). Spider embryo fixation and staining followed published procedures (*87*). Excess halocarbon oil was removed from the surface of the embryos after stage 9. The embryos were dechorionated in a glass-made dish, and were swirled for two min in 5.6 % sodium hypochlorite using a pipette tip. After being washed three times with water, the embryos were transferred to a 15 mL centrifuge tube with fixative consisting of 200 µL of formaldehyde and 10 mL of heptane. The duration of fixation was around one hour, and then the embryos were washed several times in 100% ethyl alcohol. We removed the vitelline membranes of the embryos using sharp forceps (Dumont 5) and brushes under a dissection microscope. The embryos were washed several times in TBST (1× TBS and 0.1% Tween-20), incubated for 30 min in 1 µg/mL Sytox Green (Invitrogen), and then

washed in TBST again. The respiratory organs and spinnerets differ significantly, not only in their morphological size but also in the location of their openings at embryonic stage 10. Therefore, observing these phenotypes at the appropriate embryonic stage is sufficient. We will continue to optimize the system in the future and persist with this experiment.

Figs. S1 to S35

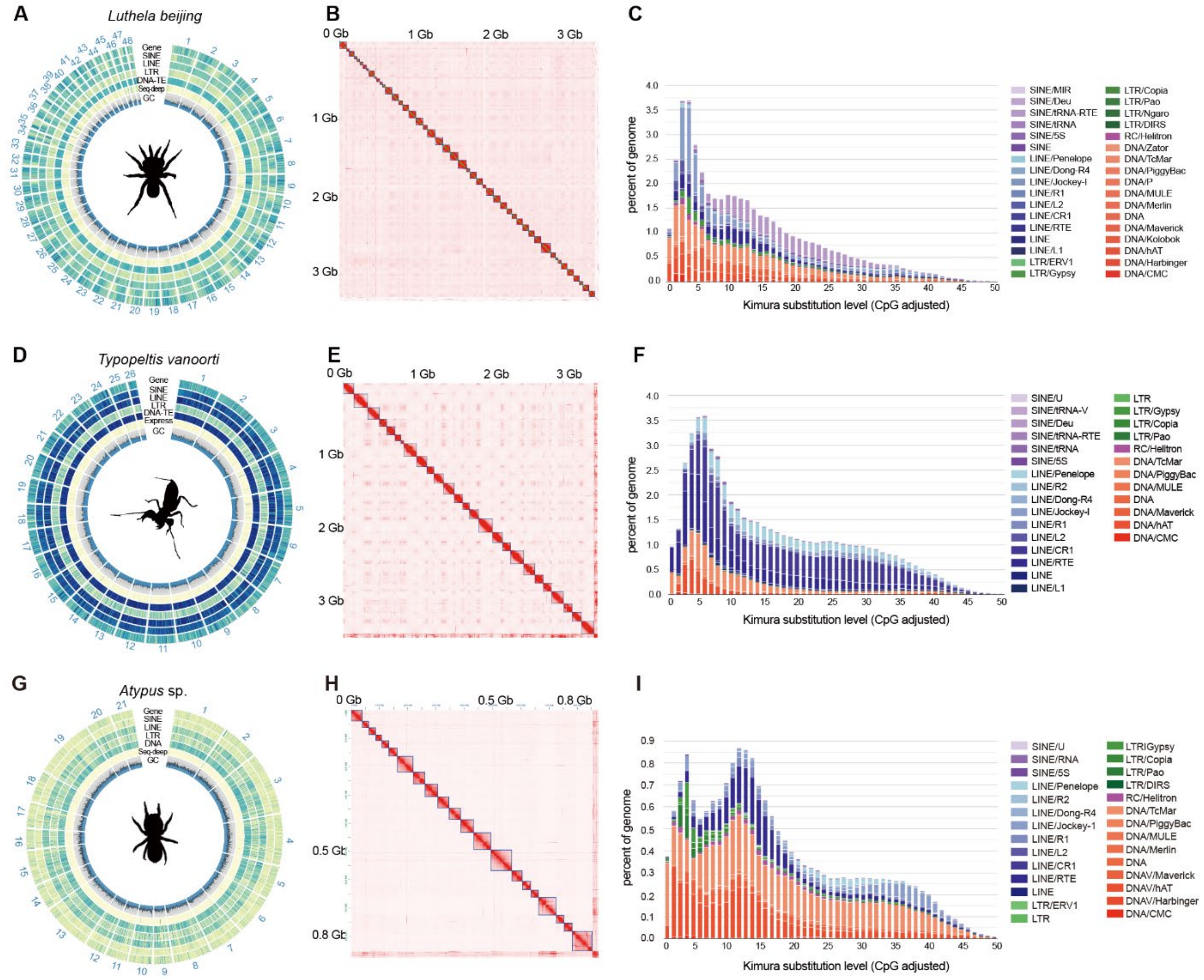

Fig. S1. Structure and evolution of the segmented spider, purse-web spider, and whip scorpion genomes. (**A**) Circos showing the genomic features of the segmented spider *Luthela beijing*. (**B**) *L. beijing* genome contig contact matrix using Hi-C data sequenced from somatic tissue at 1 Mb resolution. (**C**) The average genome sizes, TE sizes, and contents of different TE types of the spider. (**D**) Circos showing the genomic features of the whip scorpion *Typopeltis vanoorti*. (**E**) *T. vanoorti* genome contig contact matrix using Hi-C data sequenced from somatic tissue at 1 Mb resolution. (**F**) The average genome sizes, TE sizes, and contents of different TE types of the whip scorpion. (**G**) Circos showing the genomic features of the purse-web spider *Atypus* sp. (**H**) *A.* sp. genome contig contact matrix using Hi-C data sequenced from somatic tissue at 1 Mb resolution. (**I**) The average genome sizes, TE sizes, and contents of different TE types of the spider.

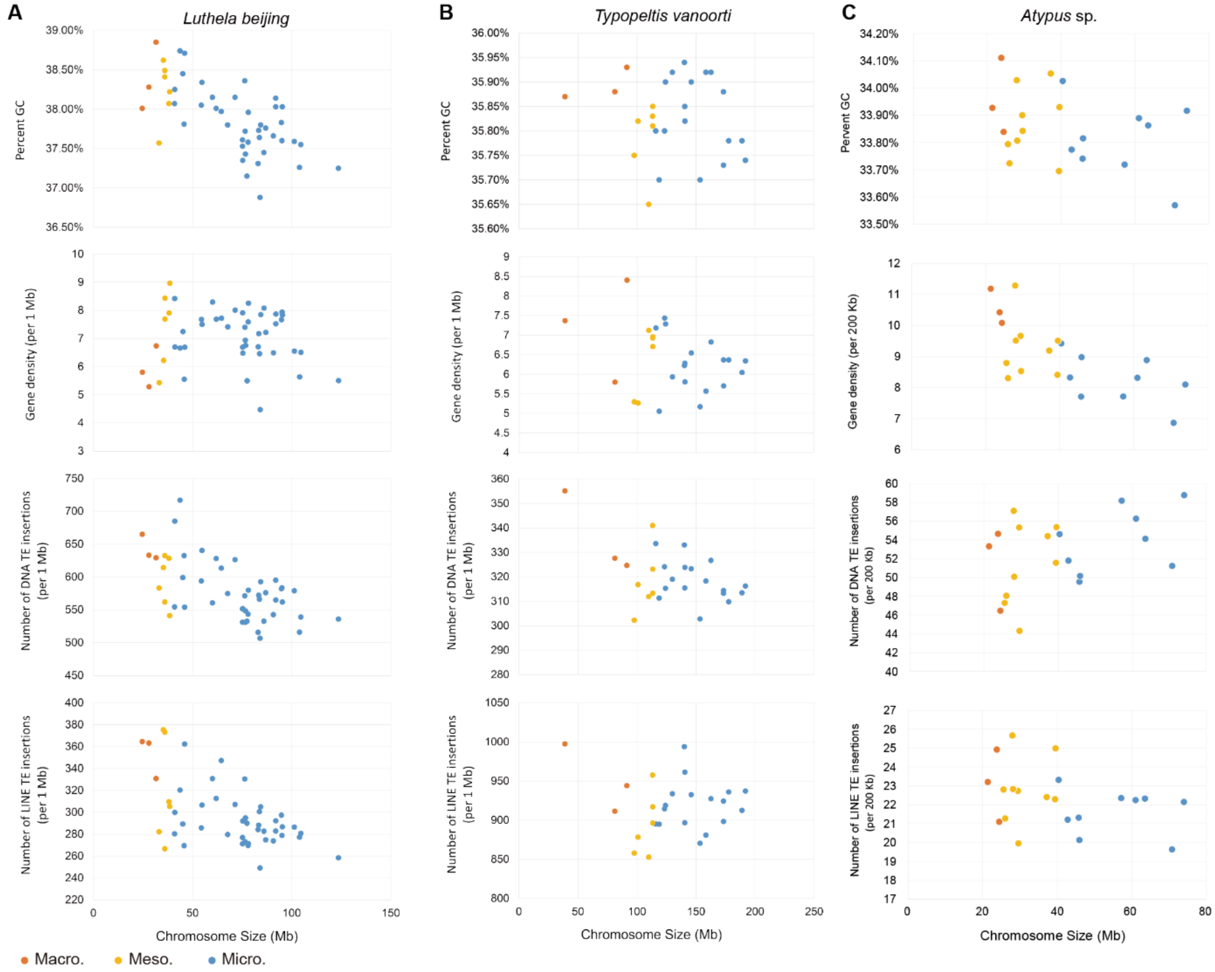

Fig. S2. Characteristics of chromosomes from the segmented spider, purse-web spider, and whip scorpion. (A) Characteristics and classification of the *L. beijing* chromosomes according to their size (x-axis) and GC%, gene density, number of DNA insertions, and number of LINE insertions per 1 Mb window. (**B**) Characteristics and classification of the *T. vanoorti* chromosomes according to their size (x-axis) and GC%, gene density, number of DNA insertions, and number of LINE insertions per 1 Mb window. (**C**) Characteristics and classification of the *Atypus* sp. chromosomes according to their size (x-axis) and GC%, gene density, number of DNA insertions, and number of LINE insertions per 200 kb window. Macro., macrochromosome; meso., mesochromosome; micro., microchromosome.

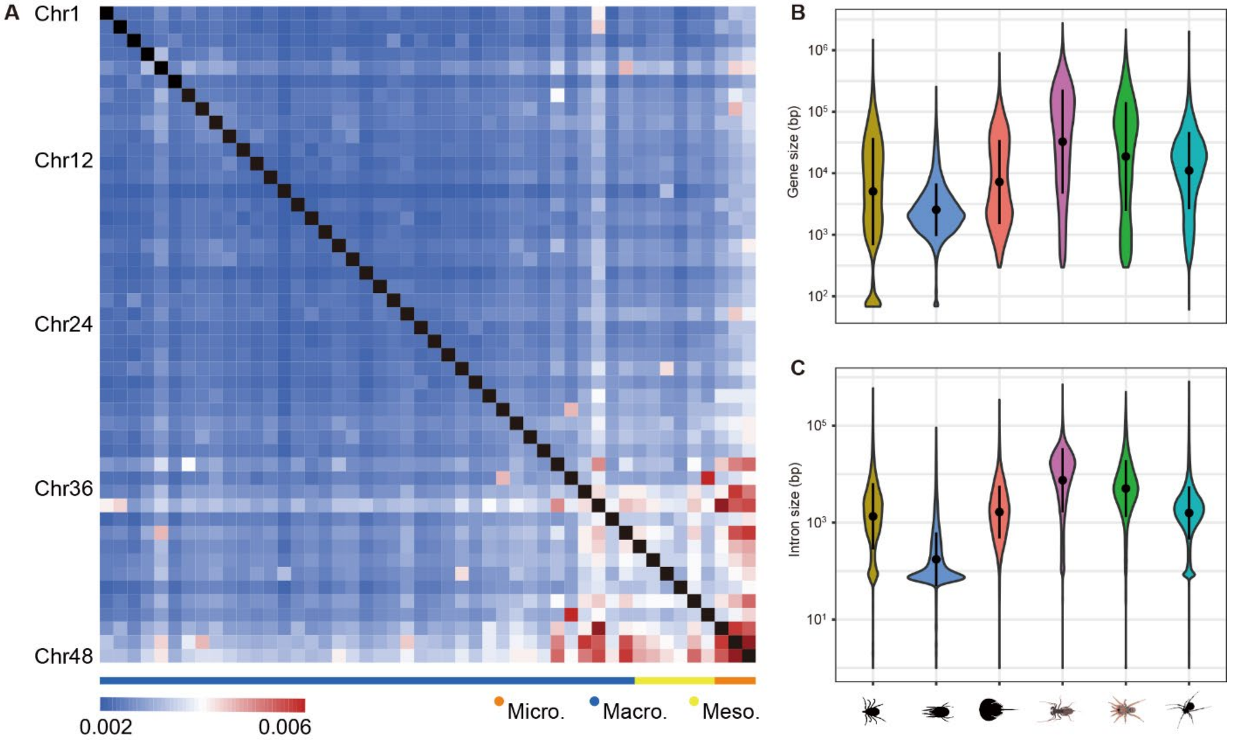

Fig. S3. Genome features of the segmented spider. (**A**) Pairwise Hi-C contact density between 48 segmented spider chromosomes showing increased interchromosomal interaction between the smallest chromosomes (microchromosomes). The color scale shows log-transformed observed/expected interchromosomal Hi-C contacts. Macro., macrochromosome; meso., mesochromosome; micro., microchromosome. (**B**)–(**C**) Distribution of gene and intron size in selected species: tick (*Ixodes scapularis*), mite (*Tetranychus urticae*), horseshoe crab (*Carcinoscorpius rotundicauda*), whip scorpion (*T. vanoorti*), segmented spider (*L. beijing*), and common house spider (*Parasteatoda tepidariorum*).

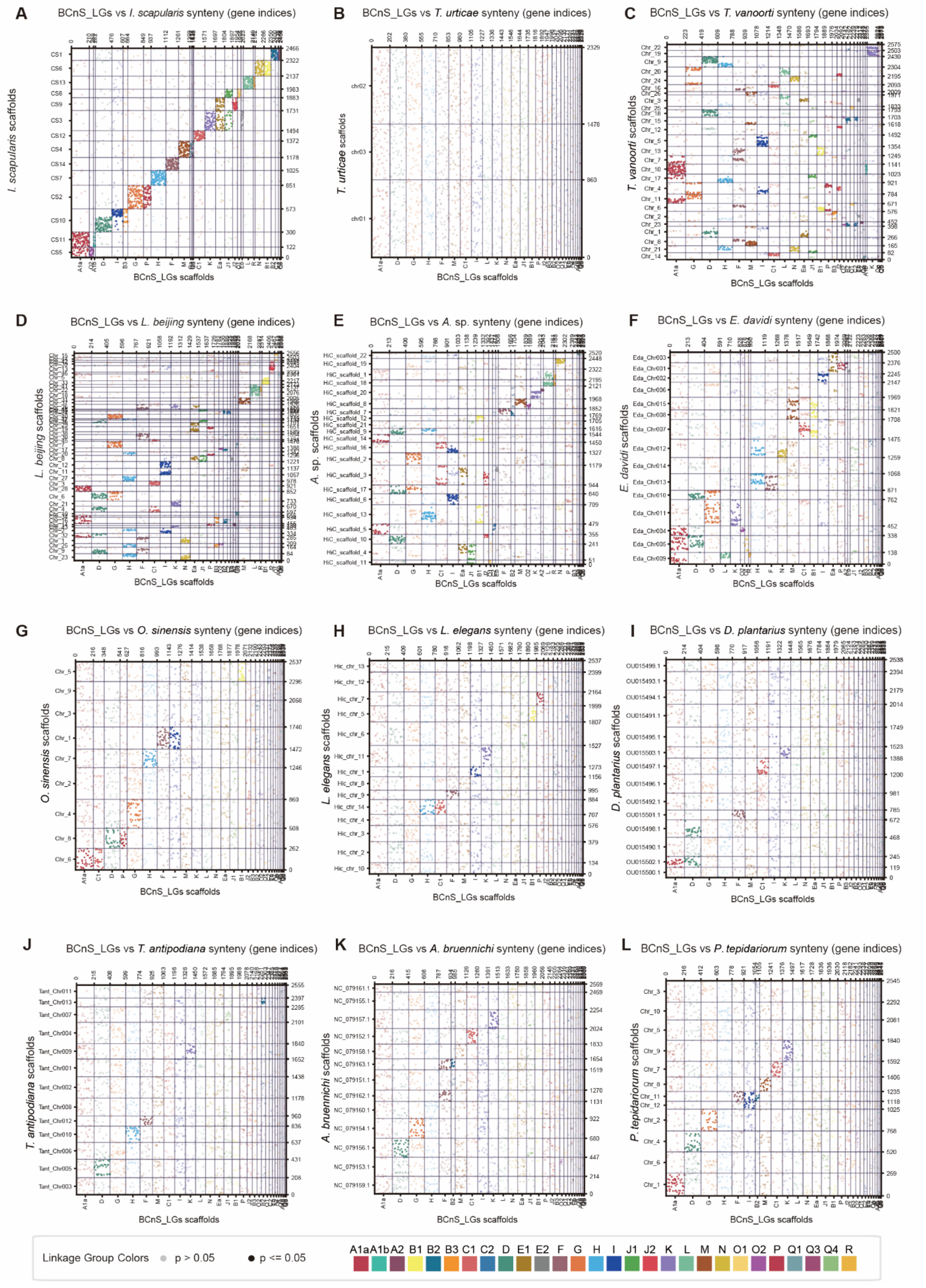

A
BCnS_LGs vs I. scapularis synteny (gene indices)
I. scapularis scaffolds
BCnS_LGs scaffolds
B
BCnS_LGs vs T. urticae synteny (gene indices)
T. urticae scaffolds
BCnS_LGs scaffolds
C
BCnS_LGs vs T. vanoorti synteny (gene indices)
T. vanoorti scaffolds
BCnS_LGs scaffolds
D
BCnS_LGs vs L. beijing synteny (gene indices)
L. beijing scaffolds
BCnS_LGs scaffolds
E
BCnS_LGs vs A. sp. synteny (gene indices)
A. sp. scaffolds
BCnS_LGs scaffolds
F
BCnS_LGs vs E. davidi synteny (gene indices)
E. davidi scaffolds
BCnS_LGs scaffolds
G
BCnS_LGs vs O. sinensis synteny (gene indices)
O. sinensis scaffolds
BCnS_LGs scaffolds
H
BCnS_LGs vs L. elegans synteny (gene indices)
L. elegans scaffolds
BCnS_LGs scaffolds
I
BCnS_LGs vs D. plantarius synteny (gene indices)
D. plantarius scaffolds
BCnS_LGs scaffolds
J
BCnS_LGs vs T. antipodiana synteny (gene indices)
T. antipodiana scaffolds
BCnS_LGs scaffolds
K
BCnS_LGs vs A. bruennichi synteny (gene indices)
A. bruennichi scaffolds
BCnS_LGs scaffolds
L
BCnS_LGs vs P. tepidariorum synteny (gene indices)
P. tepidariorum scaffolds
BCnS_LGs scaffolds
Linkage Group Colors
p > 0.05
p <= 0.05
A1a A1b A2 B1 B2 B3 C1 C2 D E1 E2 F G H I J1 J2 K L M N O1 O2 P Q1 Q3 Q4 R

Fig. S4. Oxford dotplots representing orthologous genes shared on the previously reported ancestral linkage groups (ALGs). Chromosome numbering corresponds to the homologous chelicerate linkage groups, which have lineage-specific fusion and fission in individual lineages. *I. scapularis*, with its chromosomes represented, is the most ancestral state. Each dot in the plot represents an ortholog, specifically a reciprocal best diamond blastp match between two species. The color of the dots represents different ancestral linkage groups. Dots that are a solid color are in cells with an FET p-value less than or equal to 0.05. Dots that are translucent are in cells with an FET p-value greater than 0.05. (**A**) The macrosynteny plot between *I. scapularis* and ALGs**.** (**B**) The macrosynteny plot between *T. urticae* and ALGs**.** (**C**) The macrosynteny plot between *T. vanoorti* and ALGs**.** (**D**) The macrosynteny plot between *L. beijing* and ALGs**.** (**E**) The macrosynteny plot between *A.* sp. and ALGs**.** (**F**) The macrosynteny plot between *Ectatosticta davidi* and ALGs**.** (**G**) The macrosynteny plot between *Octonoba sinensis* and ALGs**.** (**H**) The macrosynteny plot between *Latrodectus elegans* and ALGs**.** (**I**) The macrosynteny plot between *Dolomedes plantarius* and ALGs**.** (**J**) The macrosynteny plot between *Trichonephila antipodiana* and ALGs**.** (**K**) The macrosynteny plot between *Argiope* sp. and ALGs**.** (**L**) The macrosynteny plot between *P. tepidariorum* and ALGs**.**

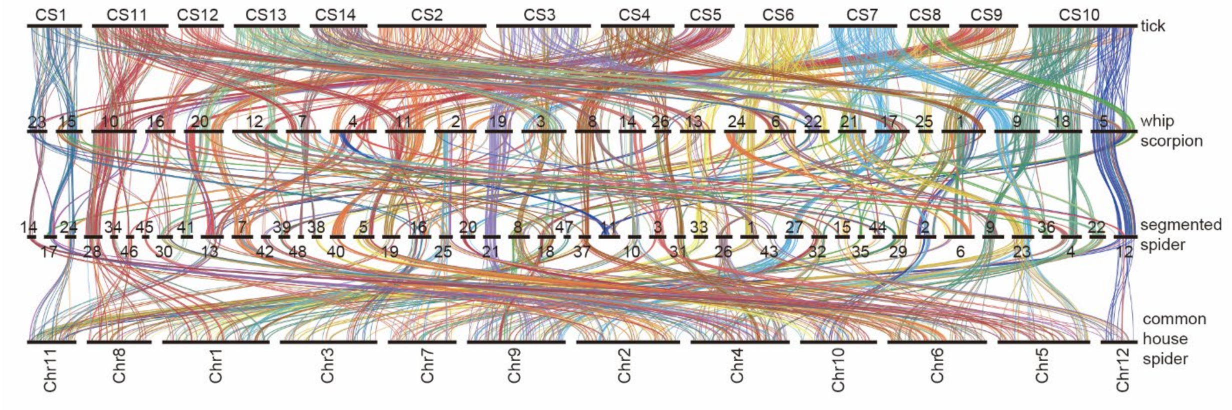

Fig. S5. Karyotypic relationships and chromosomal architecture. Syntenic orthology relationship between *I. scapularis*, *T. vanoorti*, *L. beijing*, and *P. tepidariorum* shows ancient syntenies that are stable and conserved between the tick and its metazoan ancestors, whereas the whip scorpion and spiders share a complex history of fusions and mixing after the WGD event.

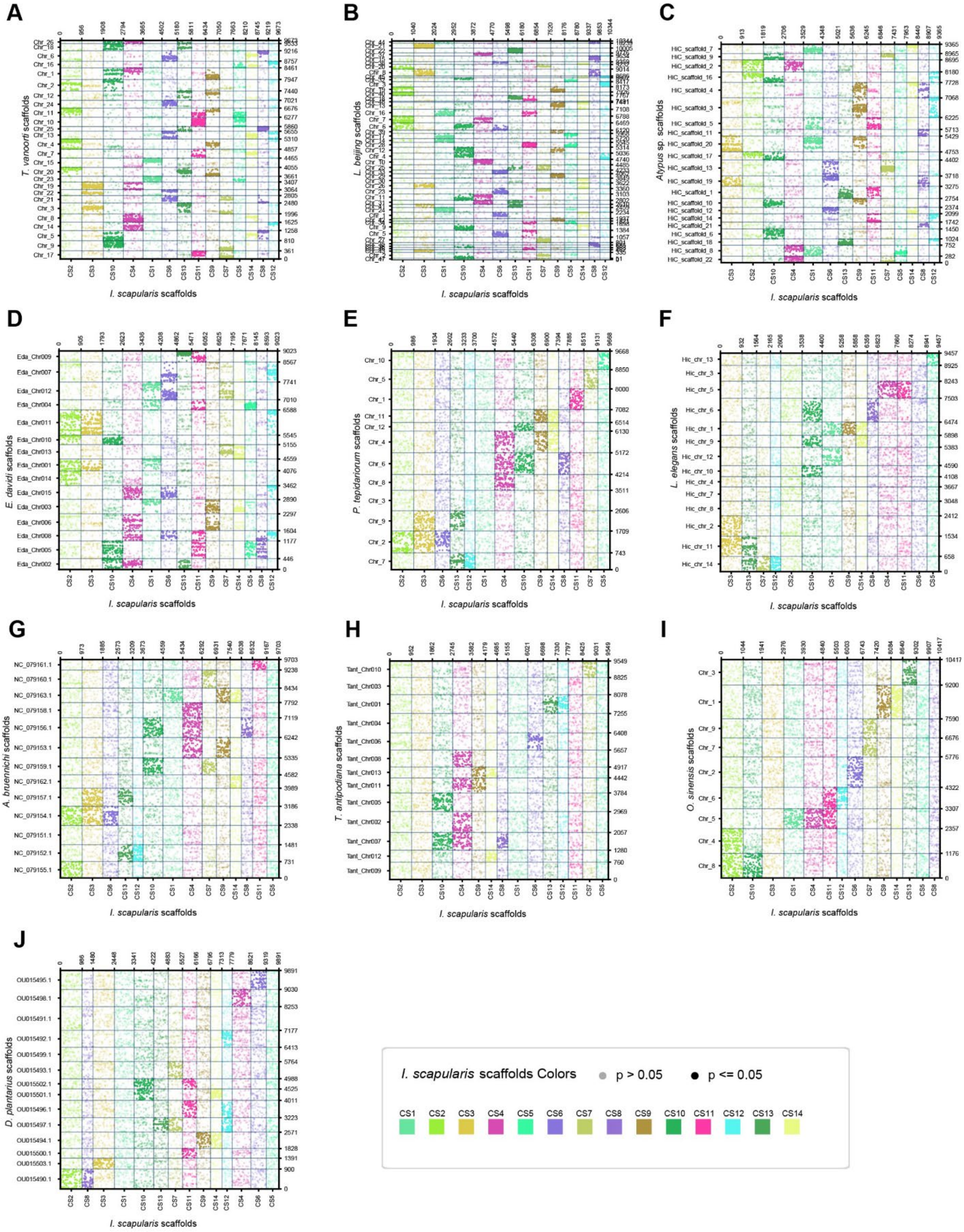

Fig. S6. Oxford dotplots representing orthologous genes shared on the tick scaffolds. Scaffold numbering corresponds to the homologous chelicerate linkage groups, which have

lineage-specific fusion and fission in individual lineages. Each dot in the plot represents an ortholog, specifically a reciprocal best diamond blastp match between two species. The color of the dots represents different ancestral linkage groups. Dots that are a solid color are in cells with an FET p-value less than or equal to 0.05. Dots that are translucent are in cells with an FET p-value greater than 0.05. (**A**) The macrosynteny plot between *T. vanoorti* and *I. scapularis*. (**B**) The macrosynteny plot between *L. beijing* and *I. scapularis*. (**C**) The macrosynteny plot between *Atypus* sp. and *I. scapularis*. (**D**) The macrosynteny plot between *E. davidi* and *I. scapularis*. (**E**)The macrosynteny plot between *P. tepidariorum* and *I. scapularis*. (**F**) The macrosynteny plot between *L. elegans* and *I. scapularis*. (**G**) The macrosynteny plot between *A. bruennichi* and *I. scapularis*. (**H**) The macrosynteny plot between *T. antipodiana* and *I. scapularis*. (**I**) The macrosynteny plot between *O. sinensis* and *I. scapularis*. (**J**) The macrosynteny plot between *D. plantarius* and *I. scapularis*.

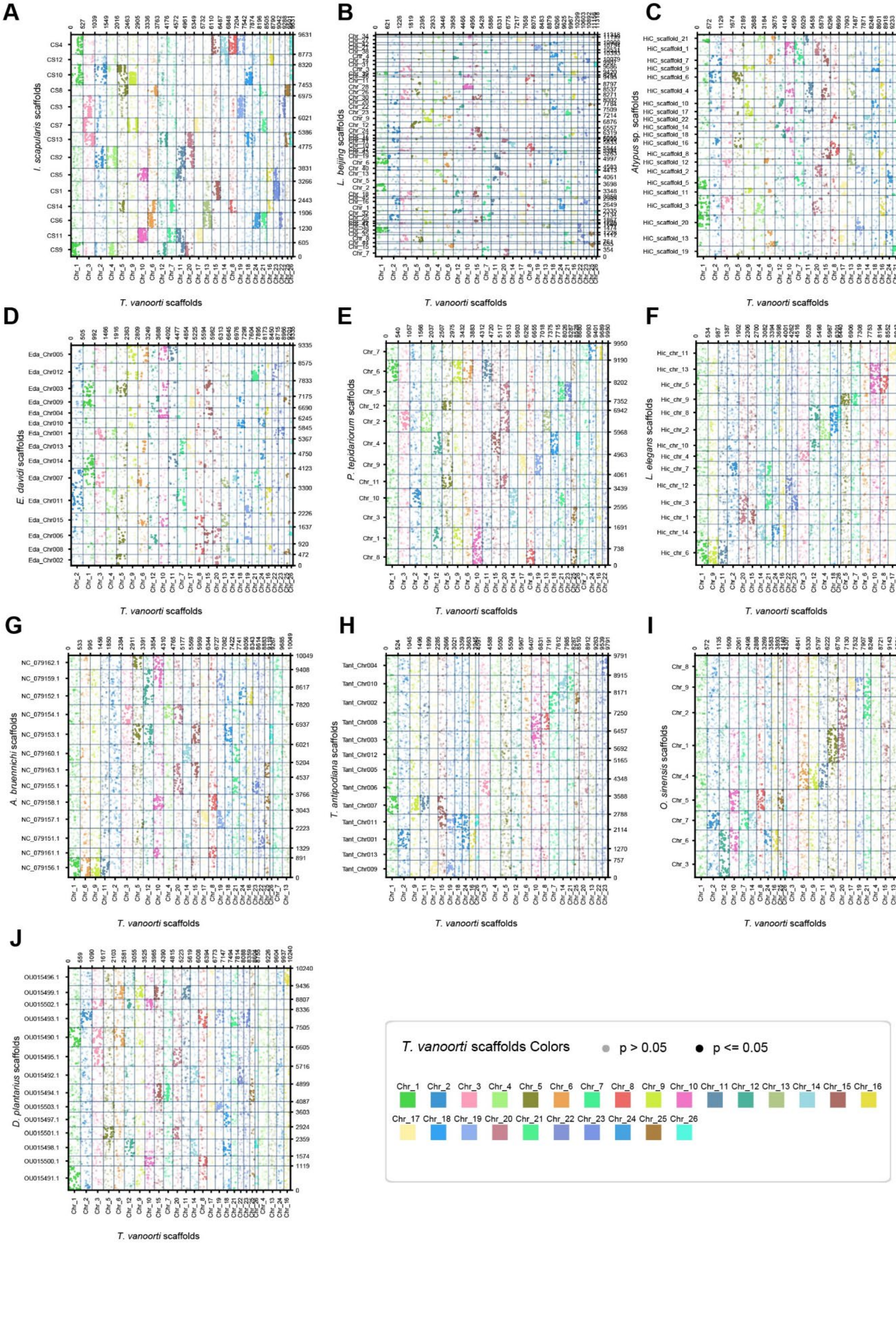
A
I. scapularis scaffolds
T. vanoorti scaffolds
B
L. beijing scaffolds
T. vanoorti scaffolds
C
Atypus sp. scaffolds
T. vanoorti scaffolds
D
E. davidi scaffolds
T. vanoorti scaffolds
E
P. tepidariorum scaffolds
T. vanoorti scaffolds
F
L. elegans scaffolds
T. vanoorti scaffolds
G
A. bruennichi scaffolds
T. vanoorti scaffolds
H
T. antipodiana scaffolds
T. vanoorti scaffolds
I
O. sirensis scaffolds
T. vanoorti scaffolds
J
D. plantarius scaffolds
T. vanoorti scaffolds
T. vanoorti scaffolds Colors
p > 0.05
p <= 0.05
Chr_1
Chr_2
Chr_3
Chr_4
Chr_5
Chr_6
Chr_7
Chr_8
Chr_9
Chr_10
Chr_11
Chr_12
Chr_13
Chr_14
Chr_15
Chr_16
Chr_17
Chr_18
Chr_19
Chr_20
Chr_21
Chr_22
Chr_23
Chr_24
Chr_25
Chr_26

Fig. S7. Oxford dotplots representing orthologous genes shared on the *T. vanoorti* scaffolds. Scaffold numbering corresponds to the homologous chelicerate linkage groups, which have lineage-specific fusion and fission in individual lineages. Each dot in the plot represents an ortholog, specifically a reciprocal best diamond blastp match between two species. The color of the dots represents different ancestral linkage groups. Dots that are a solid color are in cells with an FET p-value less than or equal to 0.05. Dots that are translucent are in cells with an FET p-value greater than 0.05. (**A**) The macrosynteny plot between *I. scapularis* and *T. vanoorti*. (**B**) The macrosynteny plot between *L. beijing* and *T. vanoorti*. (**C**) The macrosynteny plot between *A.* sp. and *T. vanoorti*. (**D**) The macrosynteny plot between *E. davidi* and *T. vanoorti*. (**E**)The macrosynteny plot between *P. tepidariorum* and *T. vanoorti*. (**F**) The macrosynteny plot between *L. elegans* and *T. vanoorti*. (**G**) The macrosynteny plot between *A. bruennichi* and *T. vanoorti*. (**H**) The macrosynteny plot between *T. antipodiana* and *T. vanoorti*. (**I**) The macrosynteny plot between *O. sinensis* and *T. vanoorti*. (**J**) The macrosynteny plot between *D. plantarius* and *T. vanoorti*.

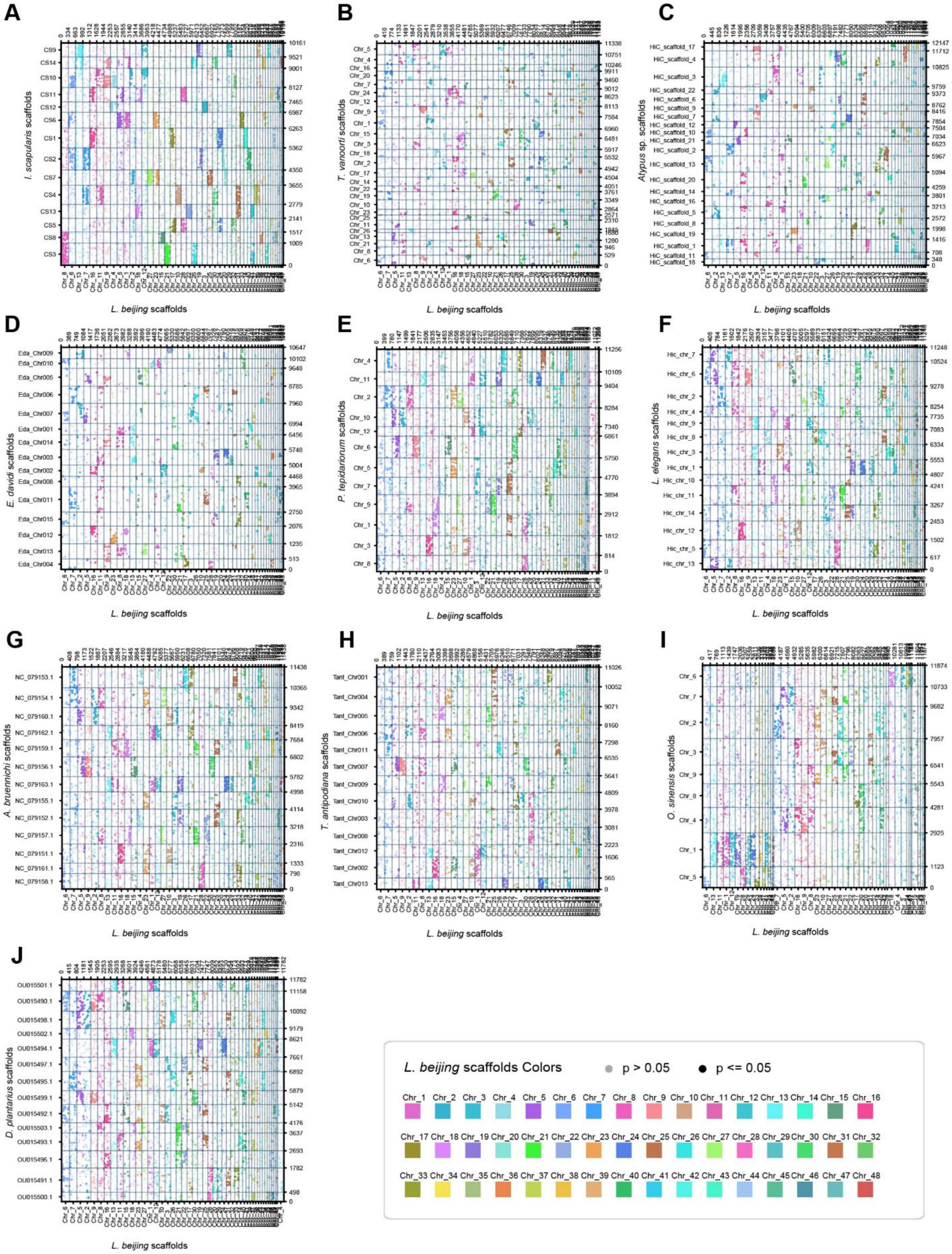
A
I. scapularis scaffolds
L. beijing scaffolds
B
T. vanoorti scaffolds
L. beijing scaffolds
C
Atypus sp. scaffolds
L. beijing scaffolds
D
E. davidi scaffolds
L. beijing scaffolds
E
P. tepidariorum scaffolds
L. beijing scaffolds
F
L. elegans scaffolds
L. beijing scaffolds
G
A. bruennichi scaffolds
L. beijing scaffolds
H
T. antipodiana scaffolds
L. beijing scaffolds
I
O. sinensis scaffolds
L. beijing scaffolds
J
D. plantarius scaffolds
L. beijing scaffolds
L. beijing scaffolds Colors
p > 0.05
p <= 0.05
Chr_1 Chr_2 Chr_3 Chr_4 Chr_5 Chr_6 Chr_7 Chr_8 Chr_9 Chr_10 Chr_11 Chr_12 Chr_13 Chr_14 Chr_15 Chr_16
Chr_17 Chr_18 Chr_19 Chr_20 Chr_21 Chr_22 Chr_23 Chr_24 Chr_25 Chr_26 Chr_27 Chr_28 Chr_29 Chr_30 Chr_31 Chr_32
Chr_33 Chr_34 Chr_35 Chr_36 Chr_37 Chr_38 Chr_39 Chr_40 Chr_41 Chr_42 Chr_43 Chr_44 Chr_45 Chr_46 Chr_47 Chr_48

Fig. S8. Oxford dotplots representing orthologous genes shared on the *L. beijing* scaffolds. Scaffold numbering corresponds to the homologous chelicerate linkage groups, which have lineage-specific fusion and fission in individual lineages. Each dot in the plot represents an ortholog, specifically a reciprocal best diamond blastp match between two species. The color of the dots represents different ancestral linkage groups. Dots that are a solid color are in cells with an FET p-value less than or equal to 0.05. Dots that are translucent are in cells with an FET p-value greater than 0.05. (**A**) The macrosynteny plot between *I. scapularis* and *L. beijing*. (**B**) The macrosynteny plot between *T. vanoorti* and *L. beijing*. (**C**) The macrosynteny plot between *A.* sp. and *L. beijing*. (**D**) The macrosynteny plot between *E. davidi* and *L. beijing*. (**E**) The macrosynteny plot between *P. tepidariorum* and *L. beijing*. (**F**) The macrosynteny plot between *L. elegans* and *L. beijing*. (**G**) The macrosynteny plot between *A. bruennichi* and *L. beijing*. (**H**) The macrosynteny plot between *T. antipodiana* and *L. beijing*. (**I**) The macrosynteny plot between *O. sinensis* and *L. beijing*. (**J**) The macrosynteny plot between *D. plantarius* and *L. beijing*.

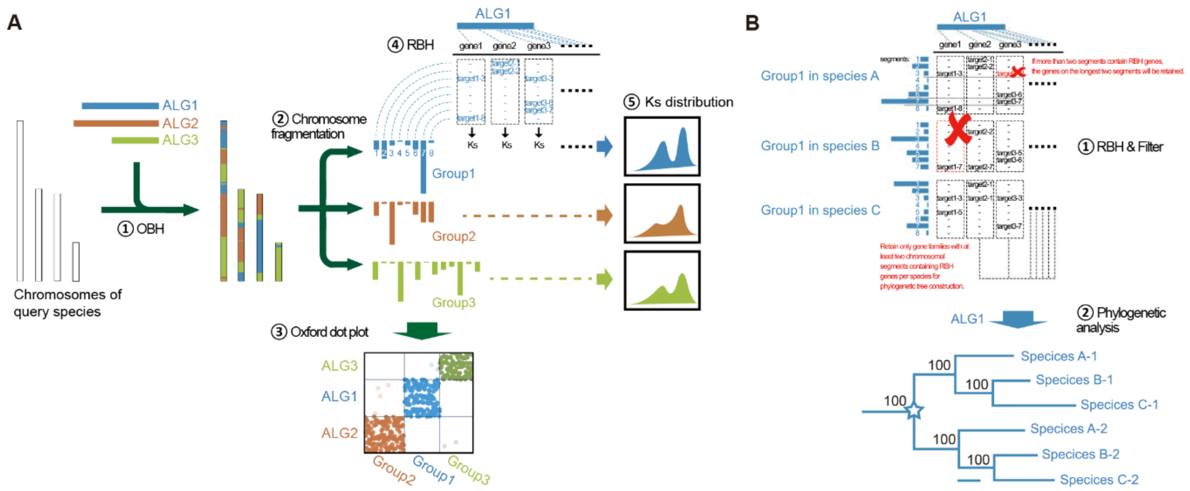

Fig. S9. Schematic Diagram of the chromosome-scale synteny method. (**A**) Chromosomal duplication analysis consisted of five key steps: one-way best hits (OBH), chromosome fragmentation, validation of homologous groups, reciprocal best-hit (RBH) identification, and synonymous substitution rate (Ks) distribution analysis. (**B**) Selection of homologous gene sets for phylogenetic analysis. Based on the sets of homologous gene duplicates identified from each BCnS ancestral linkage group (ALGs) during Ks estimation, genes containing more than two paralogous copies were filtered by comparing the lengths of the chromosomal fragments on which these copies reside. Only the paralogs located on the two longest fragments were retained. After filtering, two genes from two different fragments (*L. beijing*, *A.* sp., and *T. vanoorti*) in each homologous gene set were anchored to one unique *I. scapularis* gene.

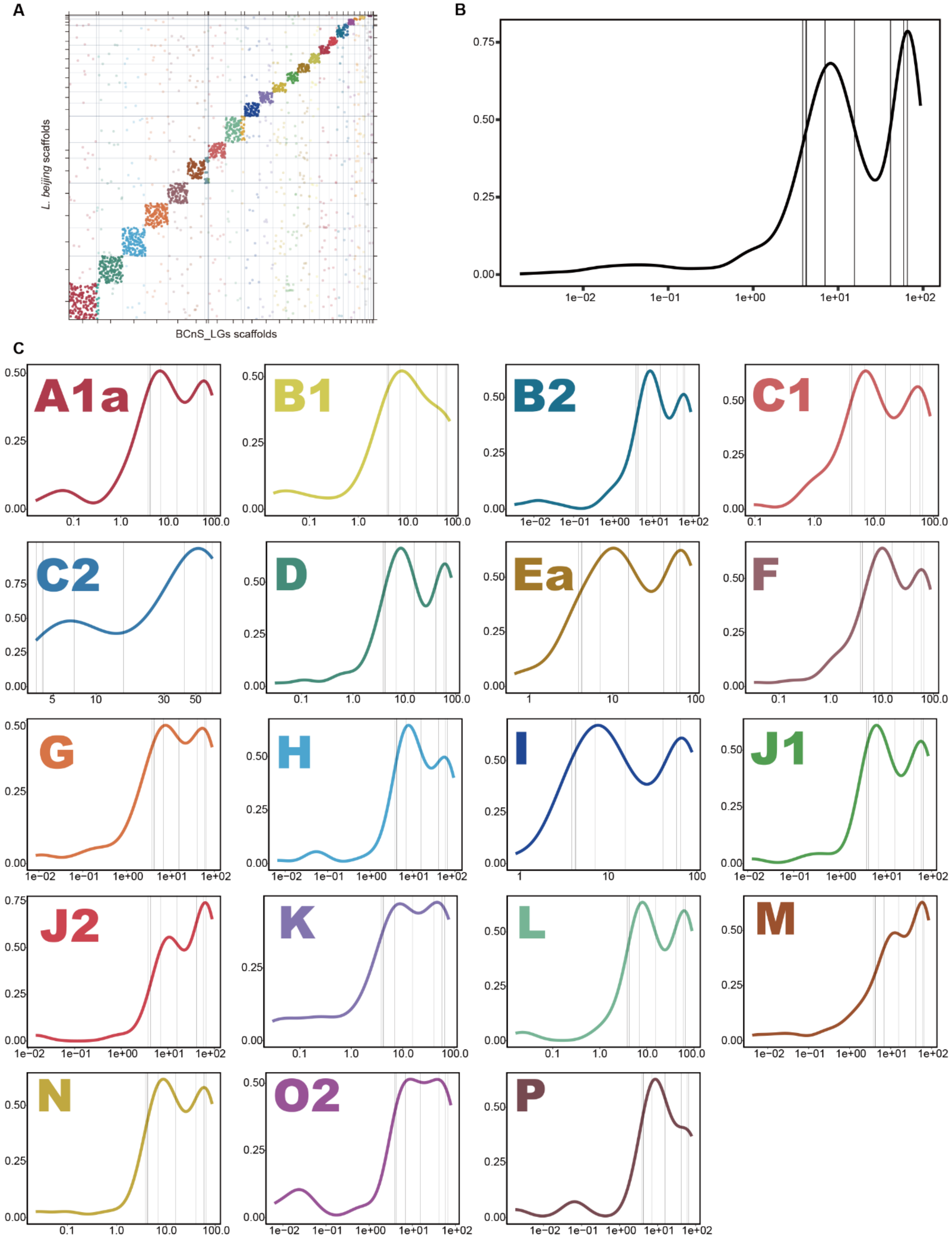

Fig. S10. Fragmented and reorganized chromosomes of the *L. beijing* based on the ALGs and dating based on synonymous substitutions across each chromosome. (**A**) Image depicts

the chromosomes/scaffolds of ALGs (x-axis) plotted against the fragmented and reorganized chromosomes of *L. beijing* (y-axis). 1,454 chromosome fragments of *L. beijing* corresponding to the same ancestral linkage group of ALGs are merged to form a reorganized chromosome. 2,161 orthologs were found between 29 ALGs scaffolds and 24 *L. beijing* reorganized chromosomes. The color of the dots represents different ancestral linkage groups. Dots that are a solid color are in cells with an FET p-value less than or equal to 0.05. Dots that are translucent are in cells with an FET p-value greater than 0.05. (**B**) The Ks distribution of all RBH gene pairs from the same ALG in *L. Beijing*. (**C**) The Ks distribution among homologous genes in each ALG (linkage groups with more than 20 homologous gene pairs). The vertical bar indicates the Ks of Hox gene pairs in this species.

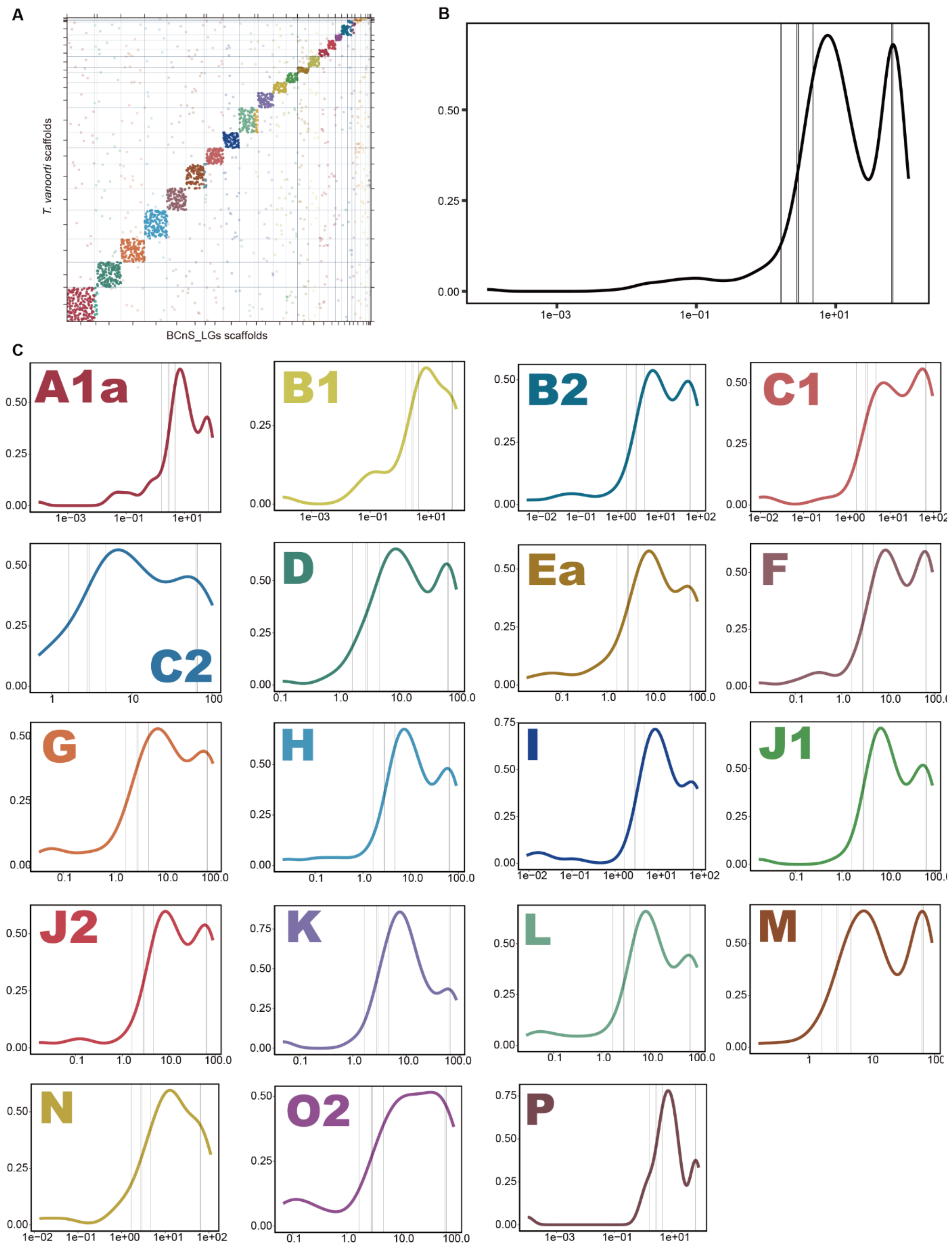

Fig. S11. Fragmented and reorganized chromosomes of the *T. vanoorti* based on the ALGs and dating based on synonymous substitutions across each chromosome. (A) Image depicts

the chromosomes/scaffolds of ALGs (x-axis) plotted against the fragmented and reorganized chromosomes of *T. vanoorti* (y-axis). 1,375 chromosome fragments of *T. vanoorti* corresponding to the same ancestral linkage group of ALGs are merged to form a reorganized chromosome. 2,222 orthologs were found between 29 ALGs scaffolds and 24 *T. vanoorti* reorganized chromosomes. The color of the dots represents different ancestral linkage groups. Dots that are a solid color are in cells with an FET p-value less than or equal to 0.05. Dots that are translucent are in cells with an FET p-value greater than 0.05. (**B**) The Ks distribution of all RBH gene pairs from the same ALG in *T. vanoorti*. (**C**) The Ks distribution among homologous genes in each ALG (linkage groups with more than 20 homologous gene pairs). The vertical bar indicates the Ks of Hox gene pairs in this species.

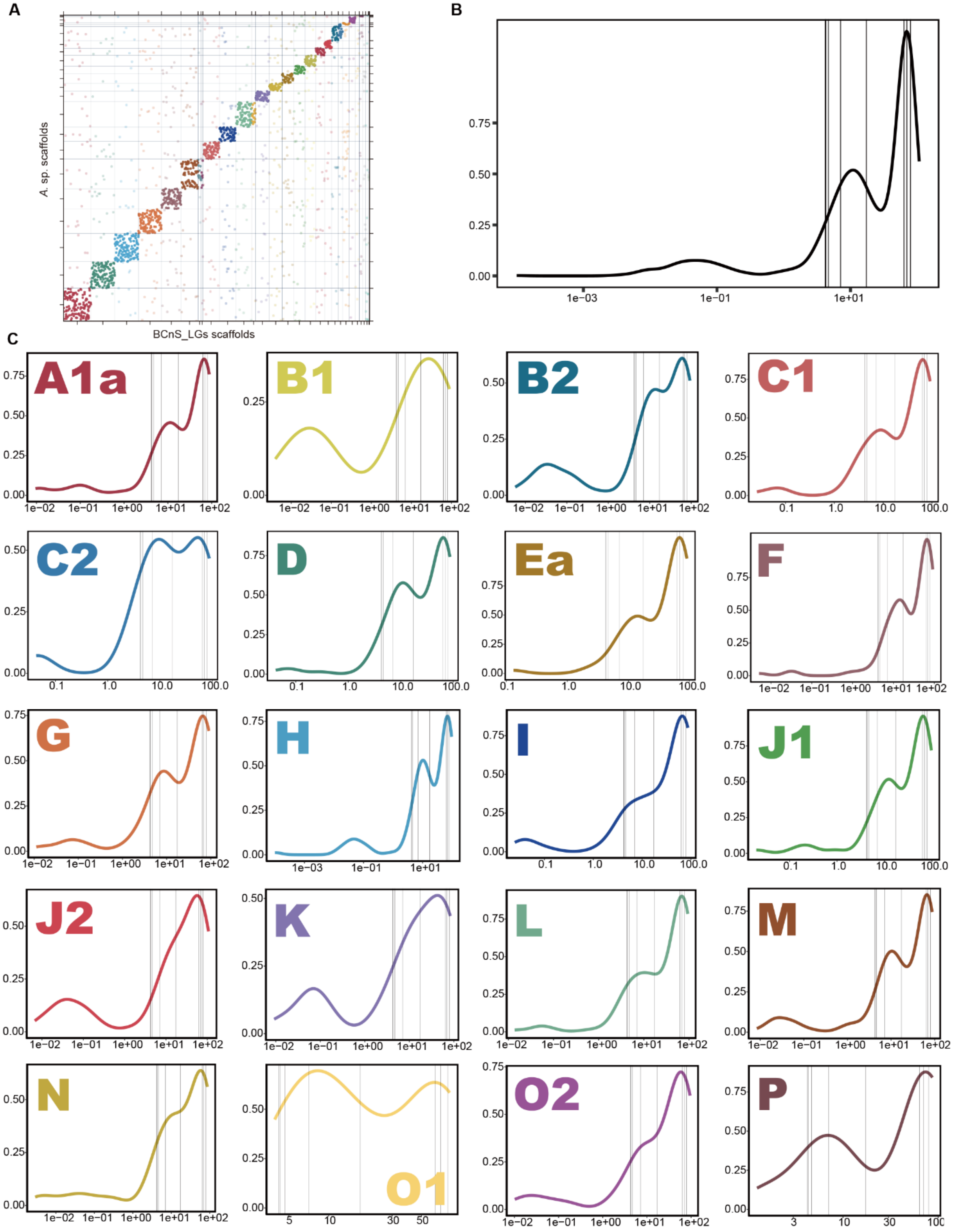

Fig. S12. Fragmented and reorganized chromosomes of the *A.* sp. based on the ALGs and dating based on synonymous substitutions across each chromosome. (**A**) Image depicts the

chromosomes/scaffolds of ALGs (x-axis) plotted against the fragmented and reorganized chromosomes of *A.* sp. (y-axis). 1,395 chromosome fragments of *A.* sp. corresponding to the same ancestral linkage group of ALGs are merged to form a reorganized chromosome. 2,002 orthologs were found between 29 ALGs scaffolds and 24 *A.* sp. reorganized chromosomes. The color of the dots represents different ancestral linkage groups. Dots that are a solid color are in cells with an FET p-value less than or equal to 0.05. Dots that are translucent are in cells with an FET p-value greater than 0.05. (**B**) The Ks distribution of all RBH gene pairs from the same ALG in *A.* sp. (**C**) The Ks distribution among homologous genes in each ALG (linkage groups with more than 20 homologous gene pairs). The vertical bar indicates the Ks of Hox gene pairs in this species.

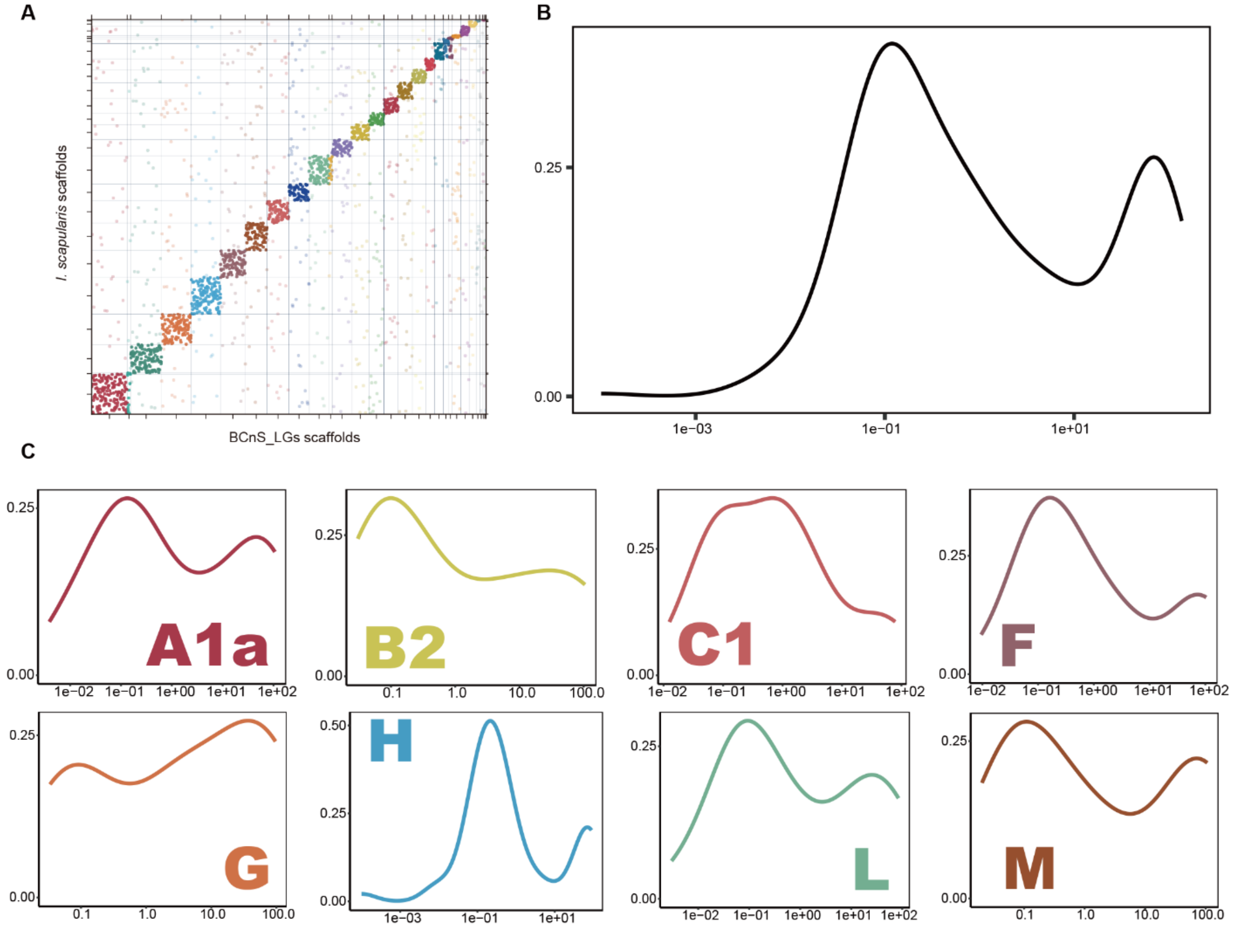

Fig. S13. Fragmented and reorganized chromosomes of the *I. scapularis* based on the ALGs and dating based on synonymous substitutions across each chromosome. (**A**) Image depicts the chromosomes/scaffolds of ALGs (x-axis) plotted against the fragmented and reorganized chromosomes of *I. scapularis*. (y-axis). 174 chromosome fragments of *I. scapularis* corresponding to the same ancestral linkage group of ALGs are merged to form a reorganized chromosome. 2,236 orthologs were found between 29 ALGs scaffolds and 25 *I. scapularis* reorganized chromosomes. The color of the dots represents different ancestral linkage groups. Dots that are a solid color are in cells with an FET p-value less than or equal to 0.05. Dots that are translucent are in cells with an FET p-value greater than 0.05. (**B**) The Ks distribution of all RBH gene pairs from the same ALG in *I. scapularis*. (**C**) The Ks distribution among homologous genes in each ALG (linkage groups with more than 20 homologous gene pairs).

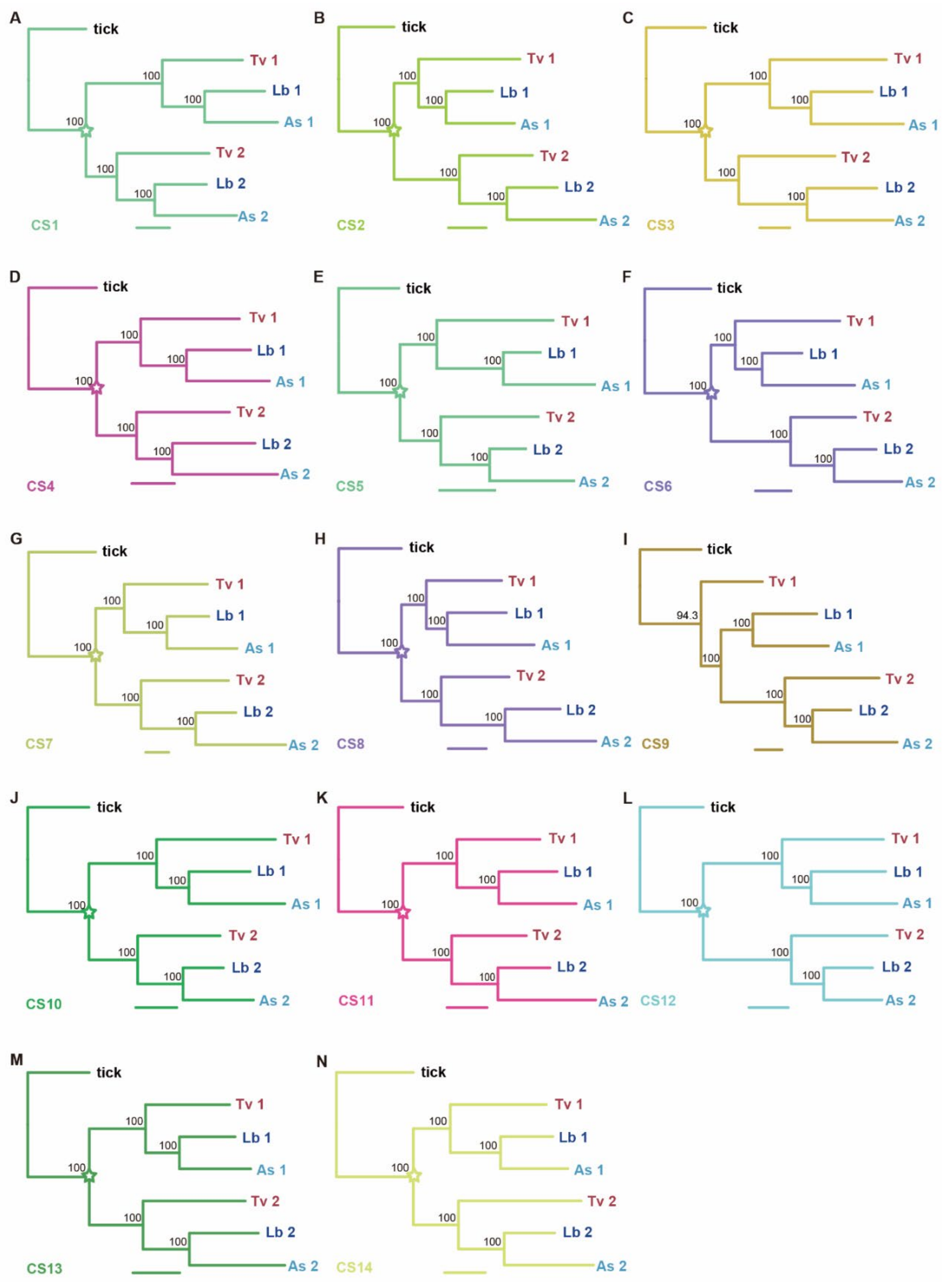

Fig. S14. The Arachnopulmonata-specific WGD is proposed based on chromosomal phylogenetic trees for each tick scaffold. (**A**)–(**N**) Chromosomal-level phylogenetic trees for each tick scaffold demonstrate the occurrence of AR WGD. The WGD is color-coded. Red and blue denote genes encoded by corresponding whip scorpion and spiders, respectively. Bootstrap

support values from IQ-Tree are marked on branches; the bar below each tree represents a genetic distance of 0.2. The stars highlight a shared WGD event.

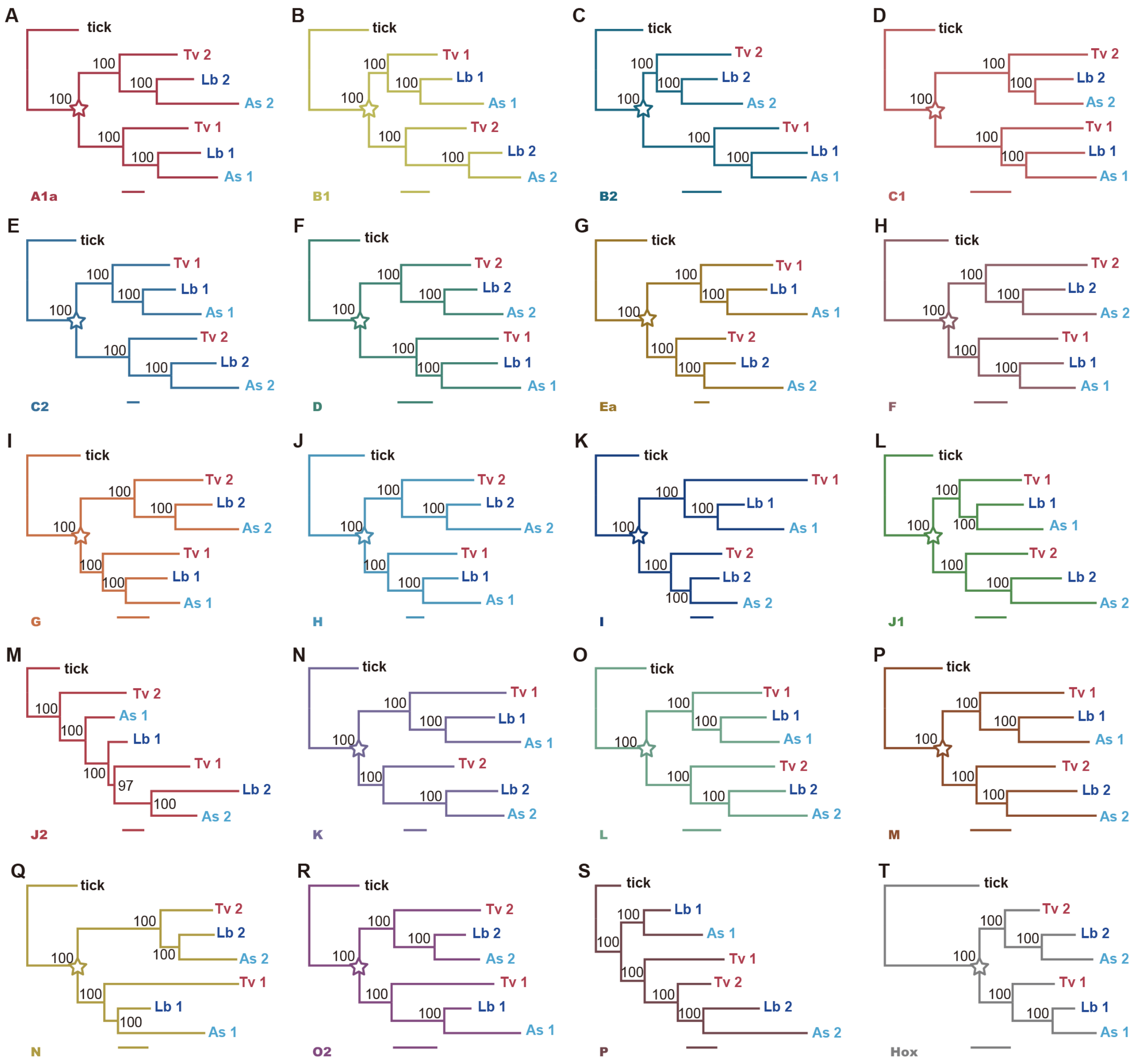

Fig. S15. The Arachnopulmonata-specific WGD is proposed based on chromosomal phylogenetic trees for each ALG. (A)–(T) Chromosomal-level phylogenetic trees for each ALG demonstrate the occurrence of AR WGD. The WGD is color-coded. Red and blue denote genes encoded by corresponding whip scorpion and spiders, respectively. Bootstrap support values from IQ-Tree are marked on branches; the bar below each tree represents a genetic distance of 0.2. The stars highlight a shared WGD event.

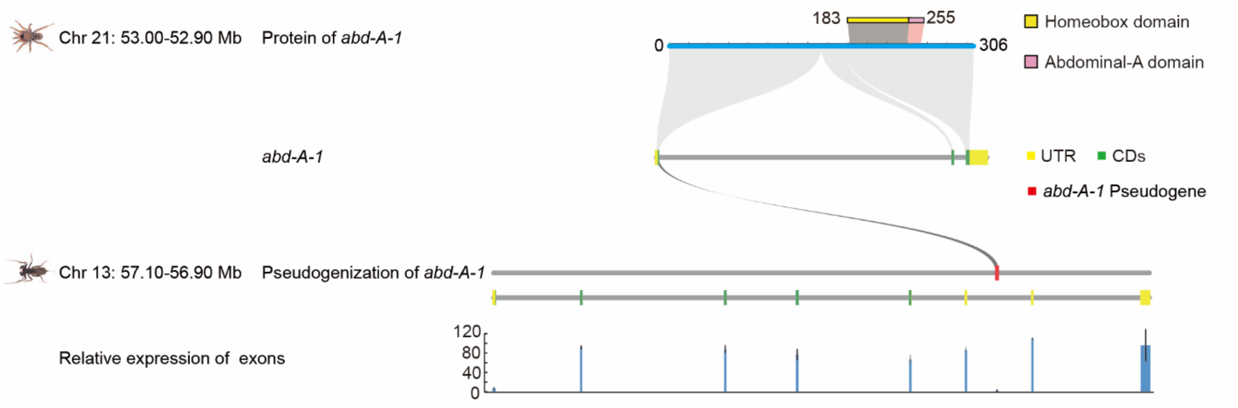

Fig. S16. Pseudogenization of the *abd-A-1* gene in the whip scorpion. The truncated *abd-A-1* gene in the whip scorpion genome is highlighted in red, which is validated in spiders.

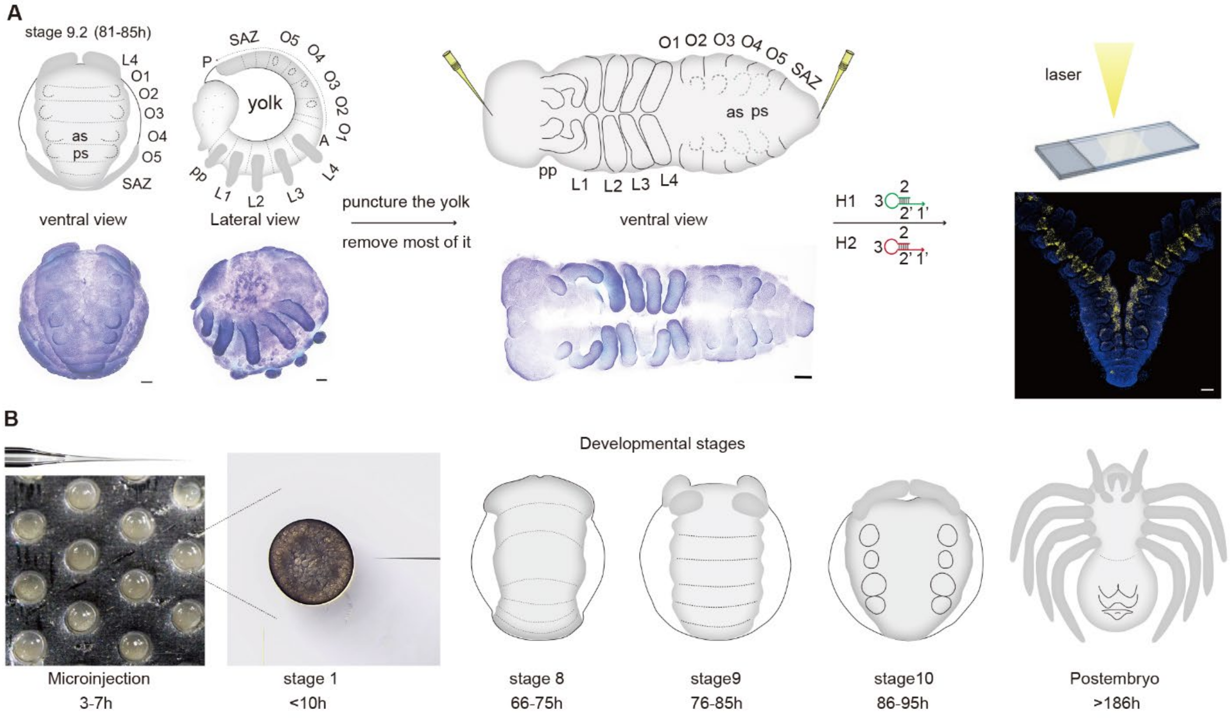

Fig. S17. Schematics of the common house spider *P. tepidariorum* at embryonic developmental stage 9.2 showing the spinneret position for HCR and CRISPR/Cas9 phenotypes. (**A**) Schematic diagram of HCR, including the dissection of spider embryos, application of probes, introduction of hairpins, sample preparation, and imaging using fluorescence microscopy. (**B**) Microinjection system construction and embryonic staging of the common house spider.

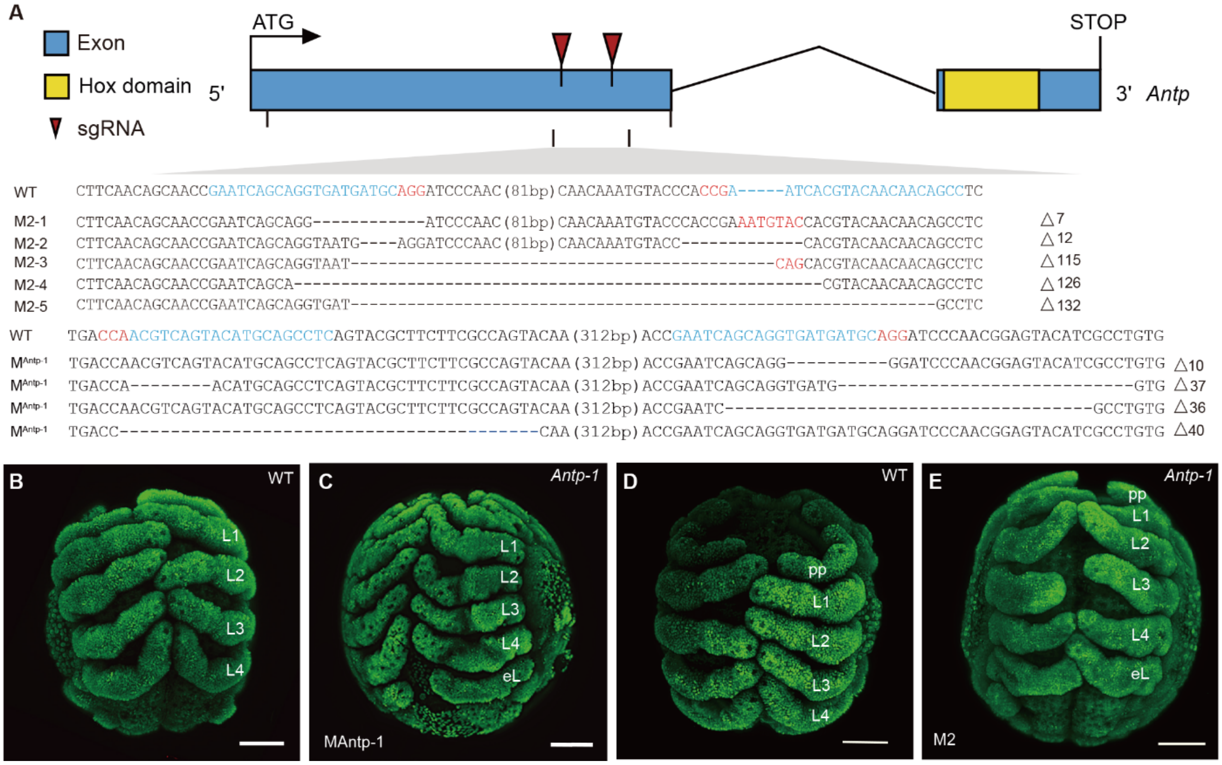

Fig. S18. CRISPR/Cas9 deletions in the gene *Antp-1* result in the development of spiders with an extra pair of legs. (**A**) Location of sgRNAs and the mutated sequences of the *P. tepidariorum Antp-1* locus. Sequences of deletion alleles confirm disruption of *Antp-1*. Blue, sgRNA sequences; Red, PAM targets. (**B**) Prosoma of wild-type embryo. (**C**) and (**D**) *Antp-1* deletion causes ten-legged embryos. pp, pedipalp; L, walking leg; eL, ectopic leg (scale bars: 100 µm).

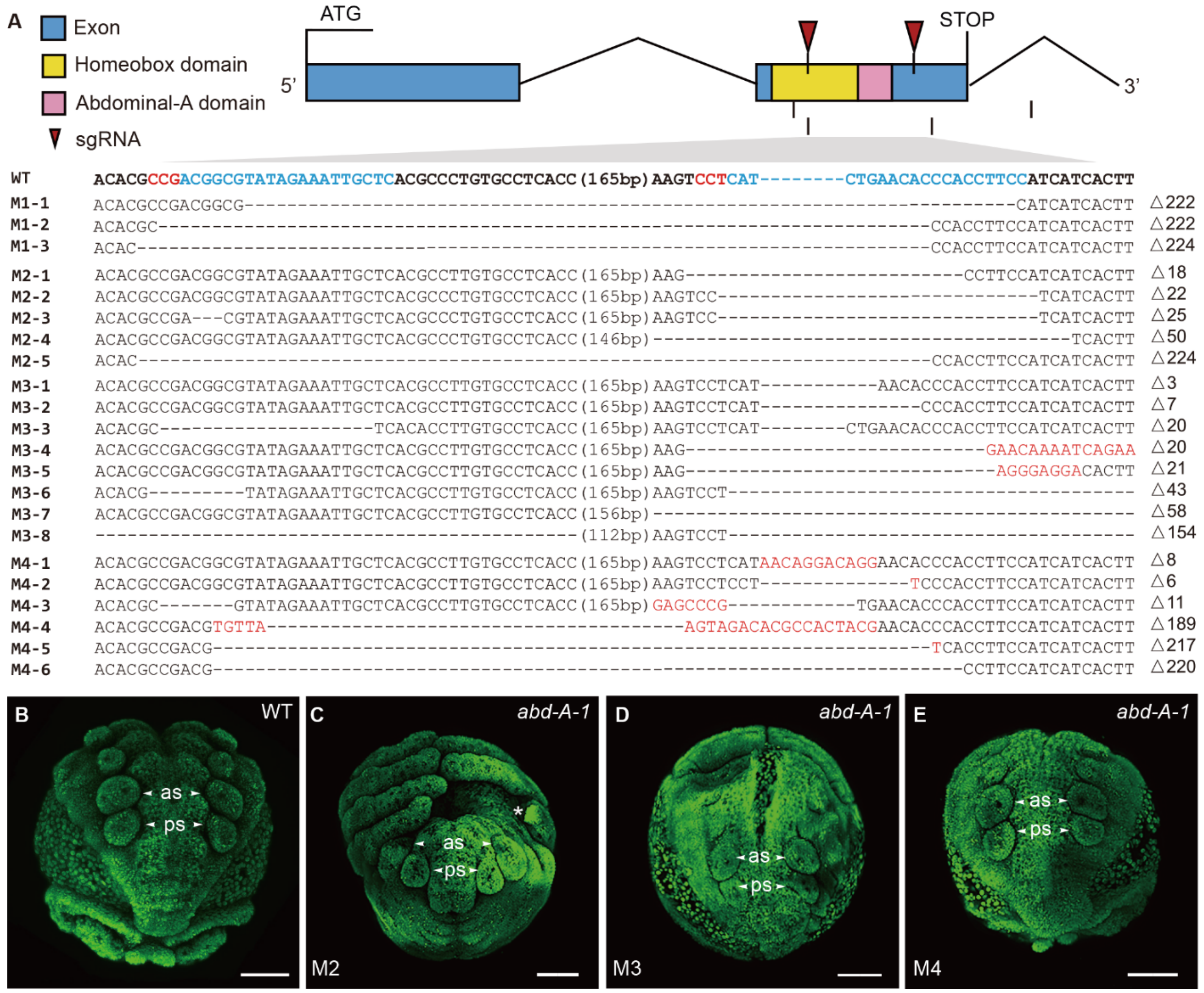

Fig. S19. CRISPR/Cas9 deletions in the gene *abd-A-1*. (**A**) Location of sgRNAs and the mutated sequences of the *P. tepidariorum abd-A-1* locus. Sequences of selected deletion alleles from the embryos (M1 to M4) confirm disruption of *abd-A-1*. Blue, sgRNA sequences; Red, PAM targets. (**B**) Opisthosoma of wild-type embryo. (**C**)–(**E**) No spinneret phenotype after the knockout of *abd-A-1*. as, anterior spinneret bud; ps, posterior spinneret bud; asterisks, the mechanically damaged part (scale bars: 100 μm).

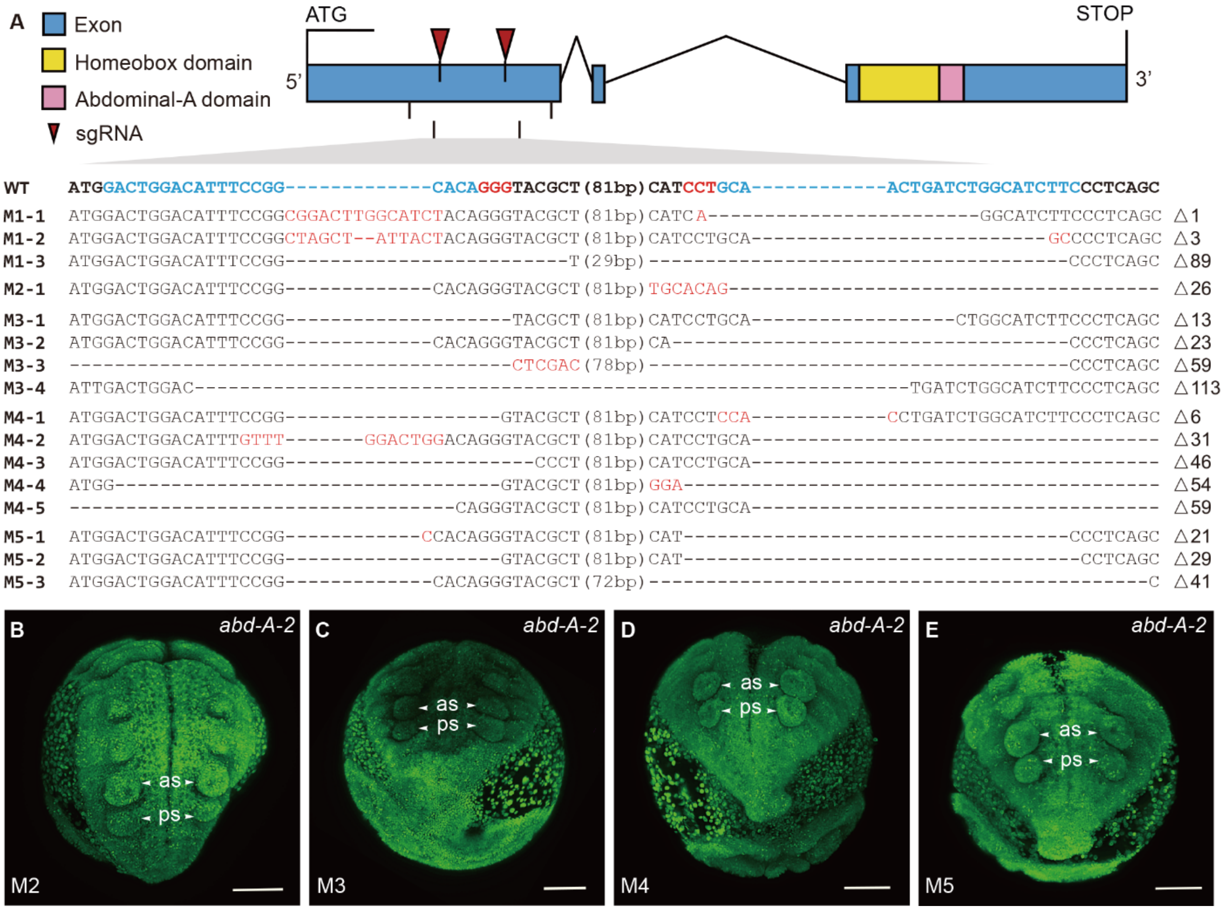

Fig. S20. CRISPR/Cas9 deletions in the gene *abd-A-2*. (**A**) Location of sgRNAs and the mutated sequences of the *P. tepidariorum abd-A-2* locus. Sequences of selected deletion alleles from the embryos (M1 to M5) confirm disruption of *abd-A-2*. Blue, sgRNA sequences; Red, PAM targets. (**B**)–(**E**) No spinneret phenotype after the knockout of *abd-A-2*. as, anterior spinneret bud; ps, posterior spinneret bud (scale bars: 100 μm).

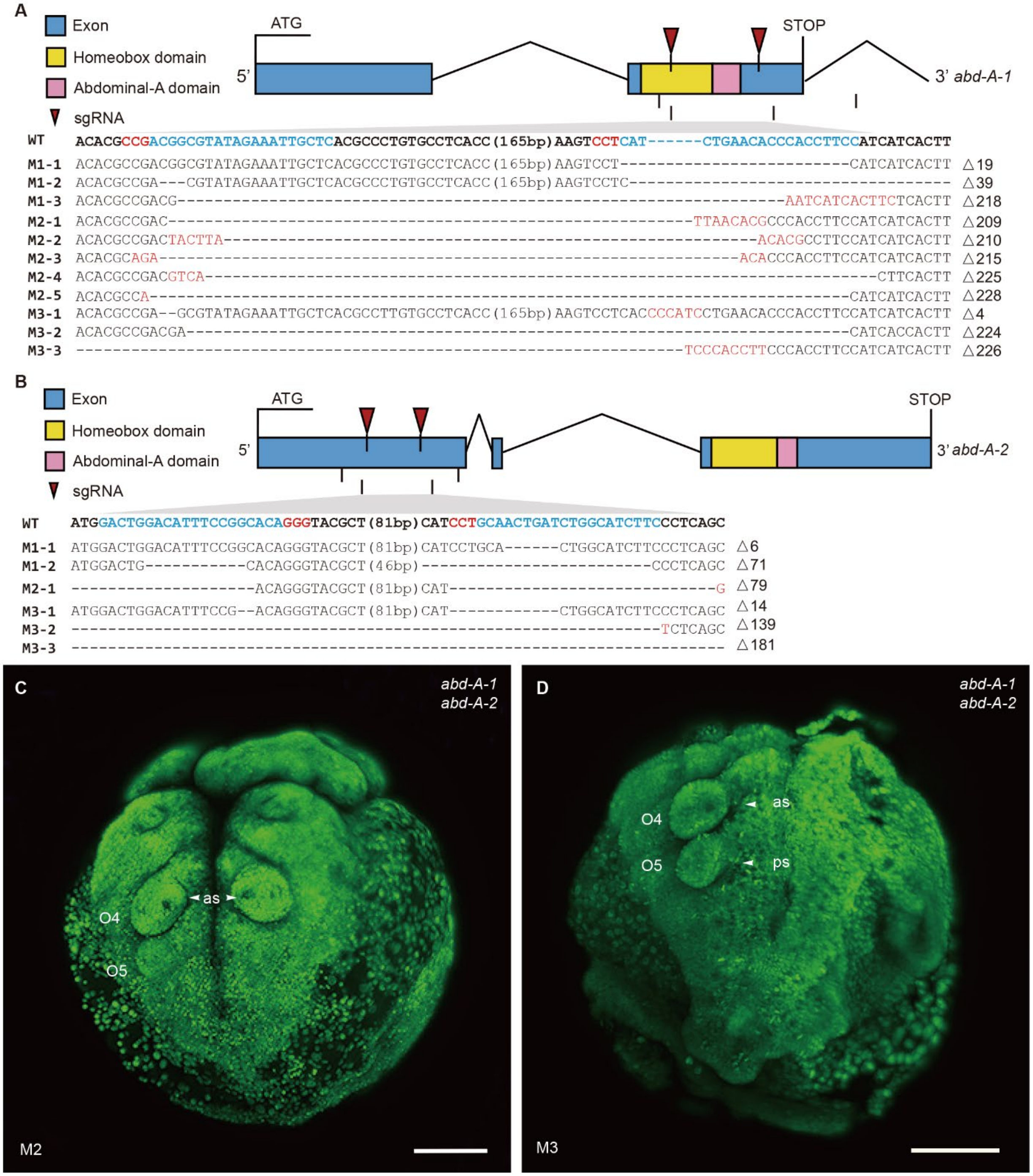

Fig. S21. CRISPR/Cas9 deletions in the genes *abd-A-1* and *abd-A-2* result in spinneret-loss phenotype. (**A**) and (**B**) Location of sgRNAs and the mutated sequences of the *P. tepidariorum abd-A-1* and *abd-A-2* loci. Sequences of selected deletion alleles from the embryos (M1, M2, and M3) confirm disruption of *abd-A-1* and *abd-A-2*. Blue, sgRNA sequences; Red, PAM targets. (**C**) and (**D**) The double knockout of *abd-A-1* and *abd-A-2* caused the loss of spinnerets. The actual fate of the O4 segment is difficult to identify because of the hollow in the abdominal

tissue of this individual. as, anterior spinneret bud; ps, posterior spinneret bud; O, opisthosomal segment (scale bars: 100 μm).

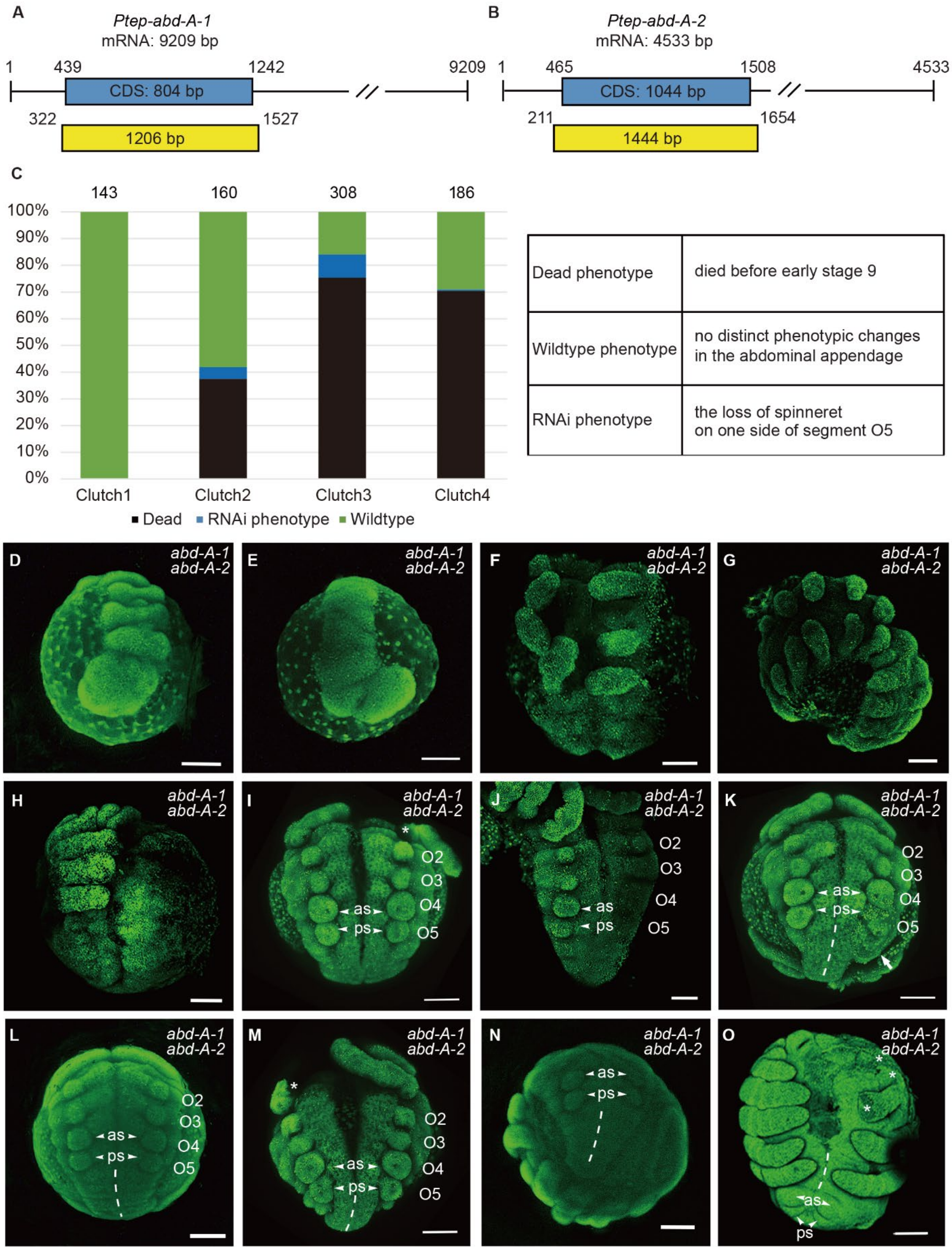

Fig. S22. Experimental design of maternal RNAi with Hox gene combinations *abd-A-1*/*abd-A-2* and phenotypic distribution. (**A**) and (**B**) *abd-A-1* and *abd-A-2* cDNA transcripts

indicating the relative location of fragments targeted for dsRNA synthesis. (**C**) Phenotypic distribution of embryos from first to fourth clutches. Numbers above bars indicate sample sizes. Colors correspond to the legend at the bottom. (**D**)–(**H**) Dead phenotype in *abd-A-1*/*abd-A-2* RNAi result. (**I**) Opisthosoma of the wild-type phenotype of RNAi. (**J**)–(**O**) *abd-A-1*/*abd-A-2* RNAi causes abnormal development of the dorsal tissue (arrow) and body axis (dashed line) and the loss of spinnerets on the right side (in **J** and **K**). as, anterior spinneret bud; ps, posterior spinneret bud; O, opisthosomal segment; asterisks, the mechanically damaged part (scale bars: 100 μm).

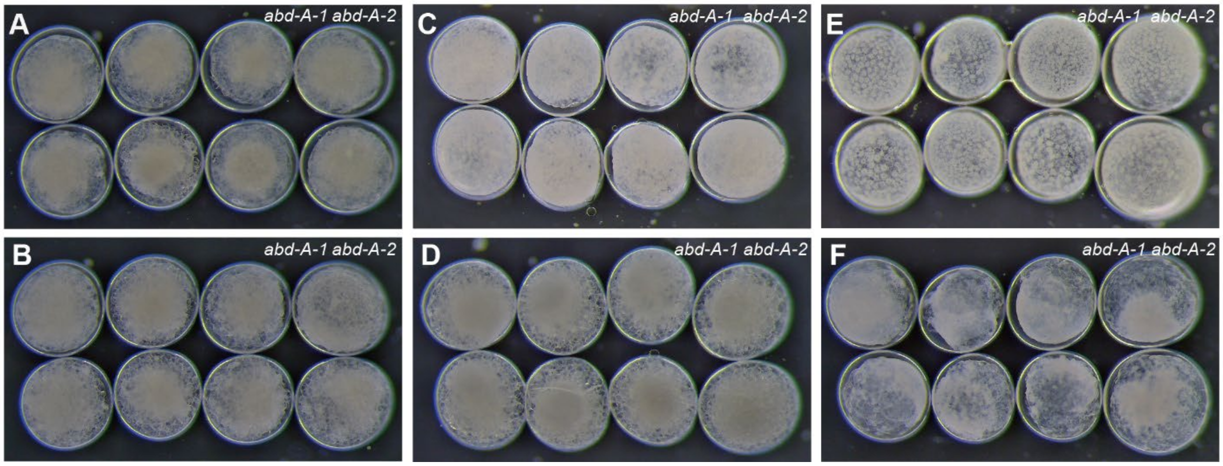

Fig. S23. The early developmental phase of the *abd-A-1/abd-A-2* RNAi embryos. (**A**)–(**B**) Embryos were collected from cocoons 2. (**C**)–(**D**) from cocoons 3. (**E**)–(**F**) from cocoons 4. RNAi targeting the Hox genes *abd-A-1* and *abd-A-2* frequently resulted in embryonic lethality prior to early stage 9.

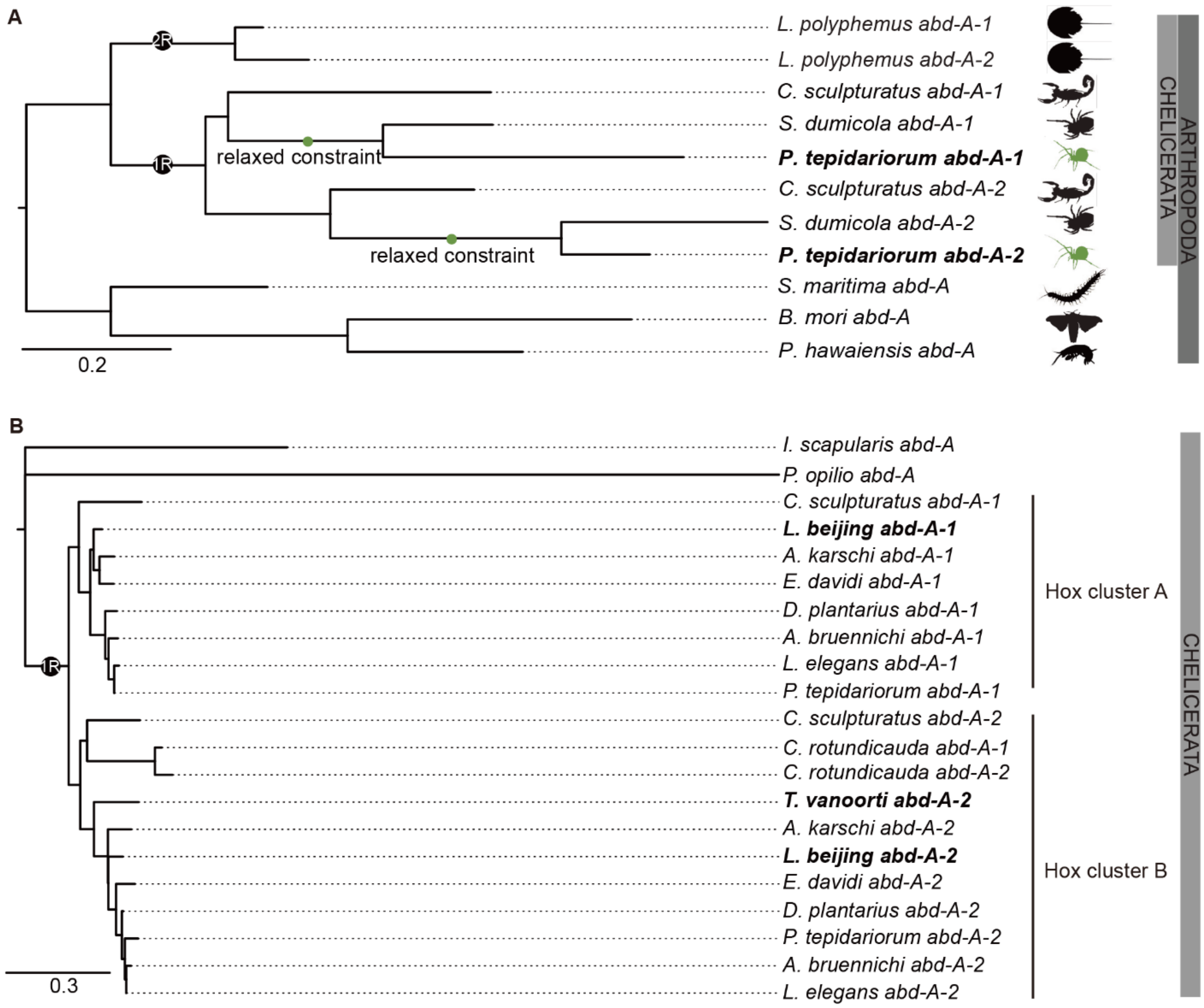

Fig. S24. Evolutionary relationships of *abd-A-1* and *abd-A-2*. (**A**) Phylogenetic tree of *abd-A* based on the alignment of nucleotide sequences of 12 species. The *abd-A* duplicates experienced relaxed constraint in the spider lineage (green dots). Phylogenetic relationships validate the WGD event in the Arachnopulmonata, the common ancestor of spiders and scorpions. (**B**) The Phylogenetic tree was constructed from 12 species based on the alignment of amino acid sequences.

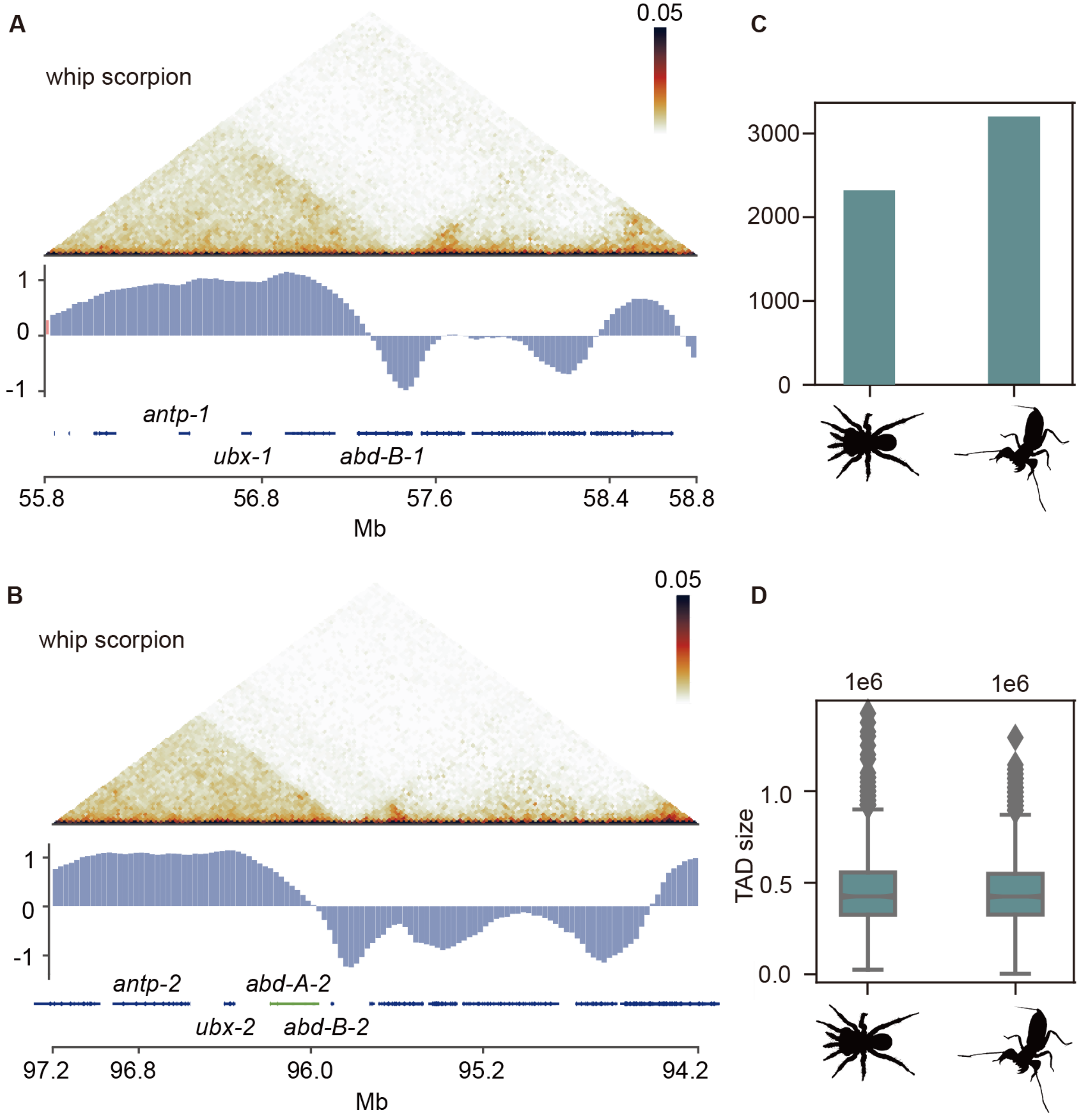

Fig. S25. FAN-C comparison for opisthosomal Hox cluster differentiation. (**A**) and (**B**) Hi-C maps from the *abd-A* locus of the whip scorpion at 25 kb resolution. (**C**) The number of TADs detected in the spider and whip scorpion. (**D**) TADs sizes observed from (C). No obvious changes in TADs were observed between spiders and the whip scorpion.

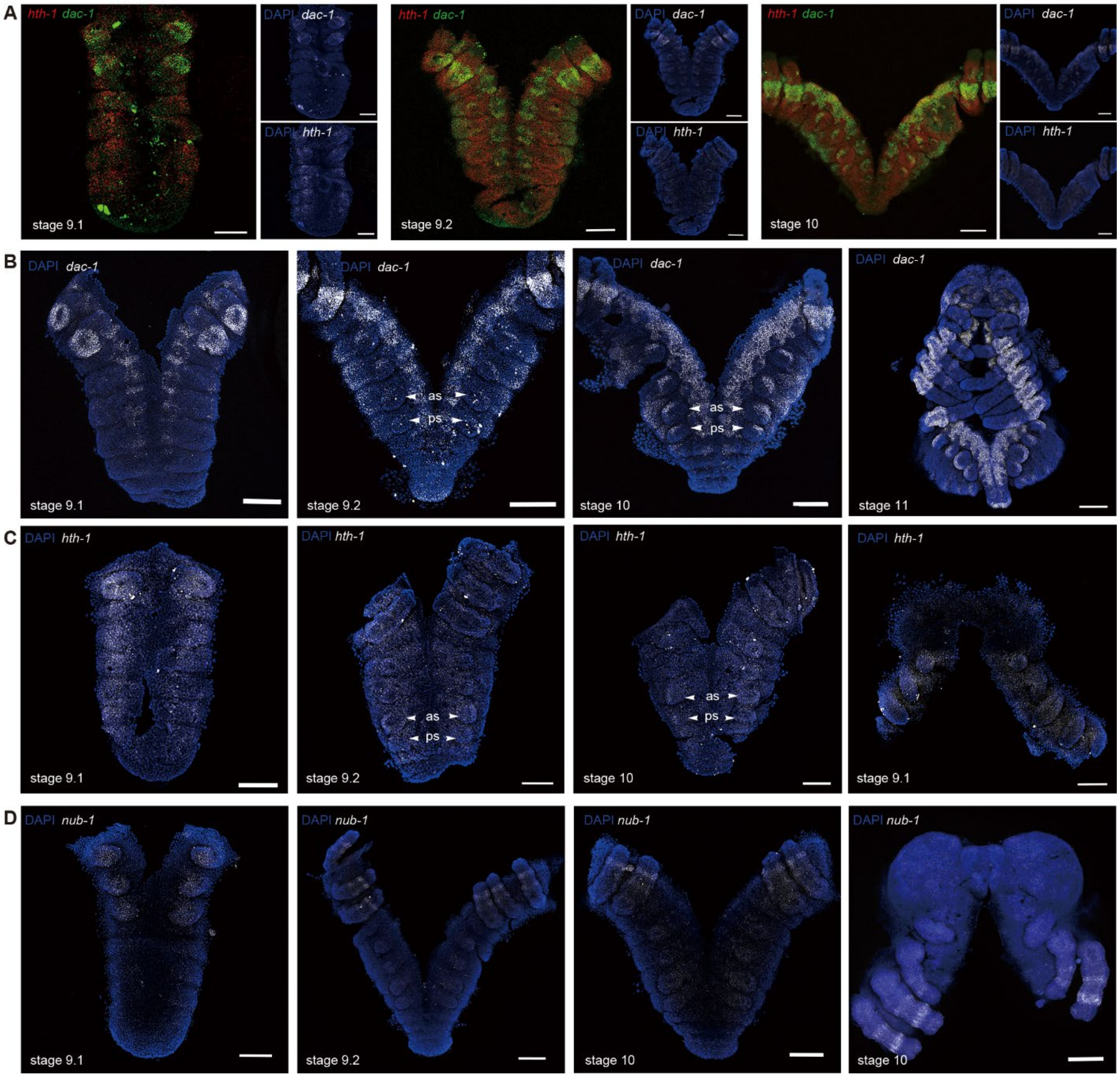

Fig. S26. HCR analysis of *dac-1*, *hth-1*, and *nub-1* in wild-type spider embryos. (**A**) HCRs showing that *hth-1* (red) and *dac-1* (green) are expressed at embryonic stages 9.1 to 10, imaged from the ventral view. (**B**) In the opisthosoma, the leg patterning gene *dac-1* (white) is primarily expressed in the developing limb buds of the O2–O5 segment. (**C**) In the opisthosoma, the leg patterning gene *hth-1* (white) is expressed uniformly, except in the posterior region of the segment addition zone (SAZ). (**D**) The gill patterning gene *nubbin* (*nub-1*) showing rings of expression in the legs and pedipalps at embryonic stages 9.1 to 10 (white). Specimens are counterstained with DAPI (blue) (Scale bars: 100 μm).

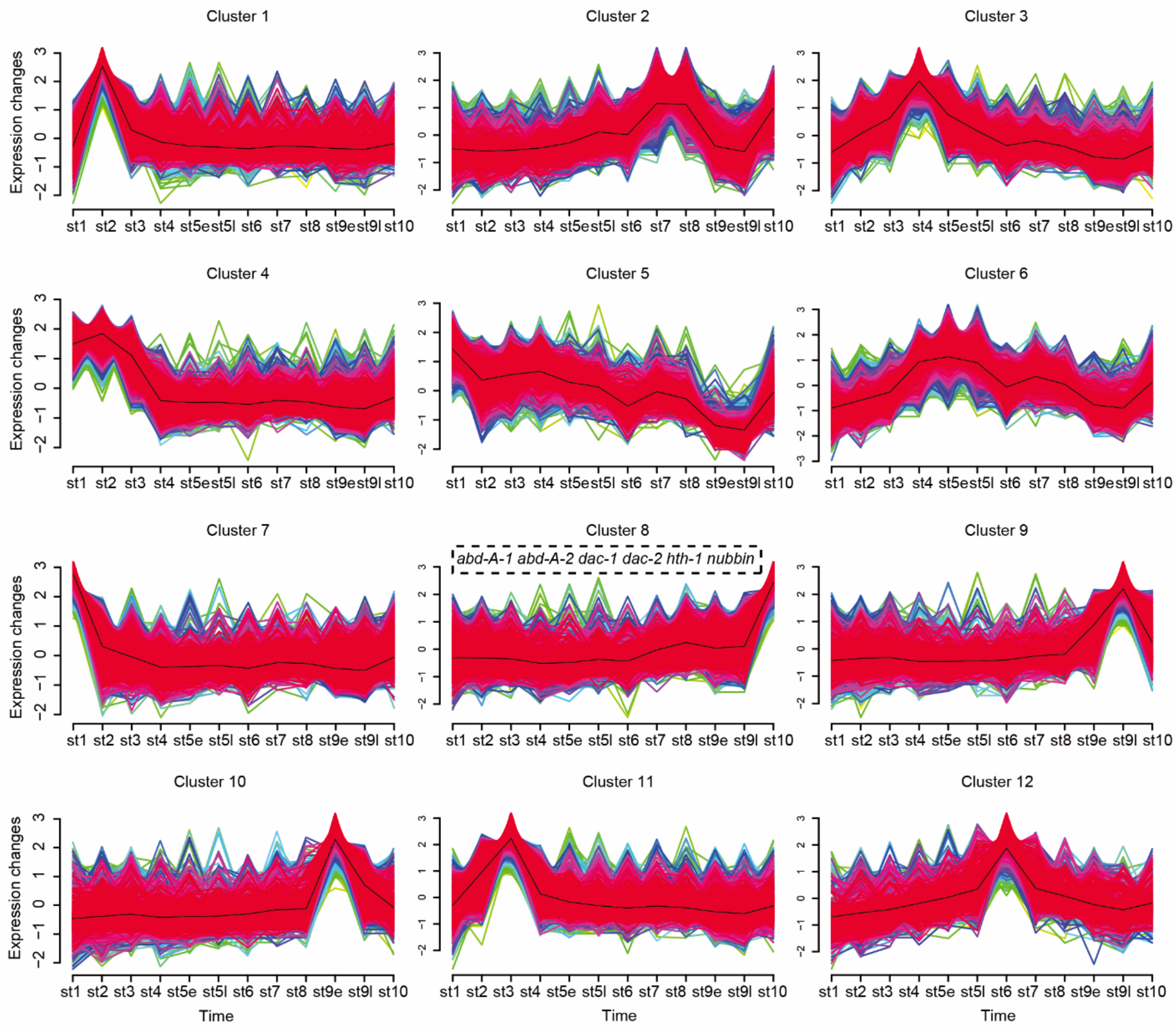

Fig. S27. Clustering result of *P. tepidariorum* RNA-seq data from stages 1 to 10. Cluster 8 contains the genes *abd-A-1*, *abd-A-2*, *dac-1*, *dac-2*, *hth-1*, and *nubbin*, suggesting that these genes have similar expression patterns.

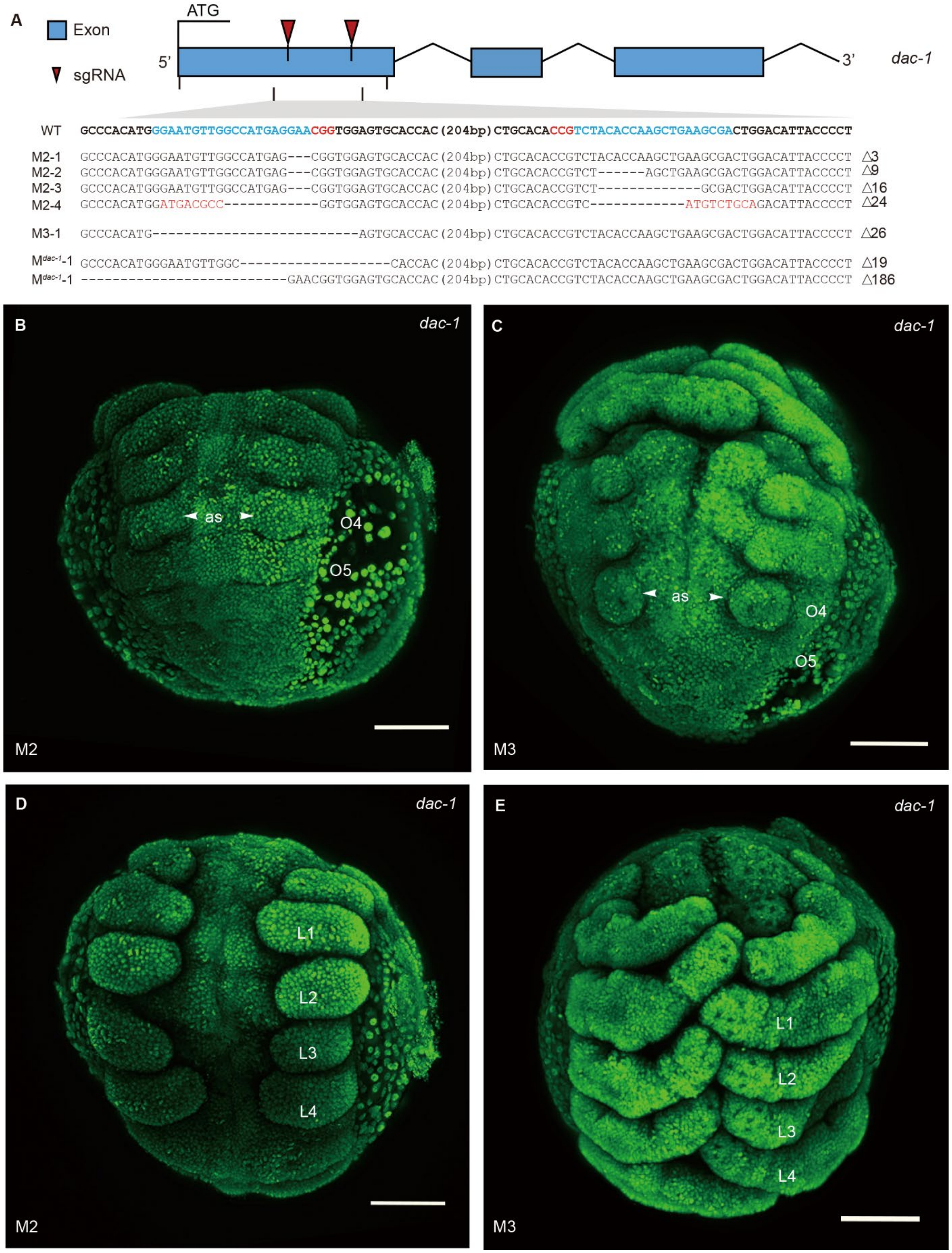

Fig. S28. CRISPR/Cas9 deletions in the *dac-1* gene. (**A**) Location of sgRNAs and the mutated sequences of the *P. tepidariorum dac-1* locus. Sequences of selected deletion alleles from the

embryos (M^{dac-1} to M3) confirm disruption of *dac-1*. (**B**) and (**C**) The knockout of *dac-1* causes the loss of spinnerets. (**D**) and (**E**) The prosoma phenotype of (B) and (C), respectively. M2 individual of *dac-1* deletions results in simplified leg patterning, whereas the M3 individual shows the mosaic phenotype with wild-type prosoma. as, anterior spinneret bud; O, opisthosomal segment; L, walking legs (Scale bars: 100 μm).

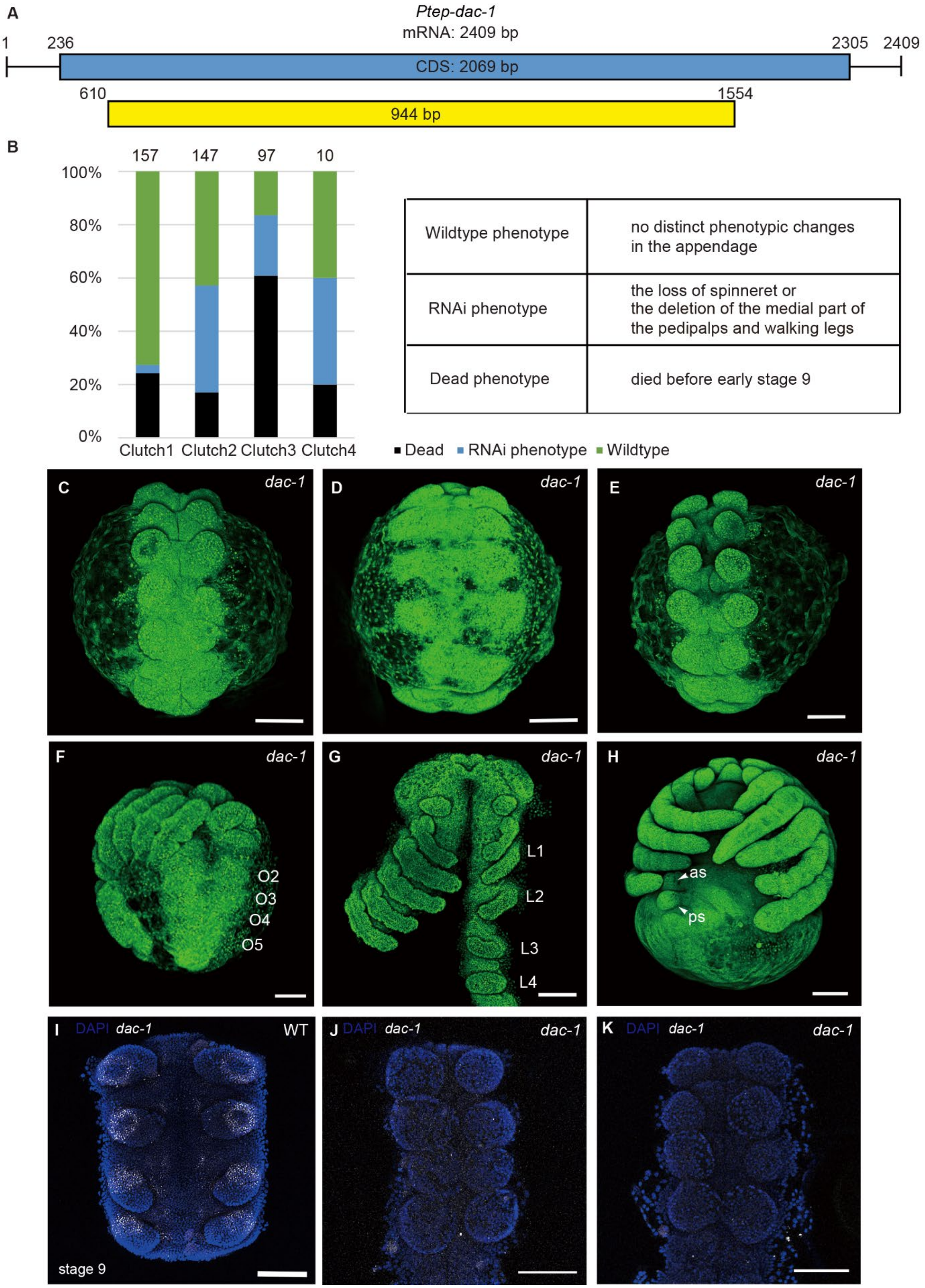
A
Ptep-dac-1
mRNA: 2409 bp
1
236
2305
2409
CDS: 2069 bp
610
1554
944 bp
B
157
147
97
10
100%
80%
60%
40%
20%
0%
Clutch1
Clutch2
Clutch3
Clutch4
Dead
RNAi phenotype
Wildtype
Wildtype phenotype
no distinct phenotypic changes in the appendage
RNAi phenotype
the loss of spinneret or the deletion of the medial part of the pedipalps and walking legs
Dead phenotype
died before early stage 9
C
dac-1
D
dac-1
E
dac-1
F
dac-1
O2
O3
O4
O5
G
dac-1
L1
L2
L3
L4
H
dac-1
as
ps
I
DAPI
dac-1
WT
stage 9
J
DAPI
dac-1
dac-1
K
DAPI
dac-1
dac-1

Fig. S29. Experimental design of maternal RNAi with *dac-1* gene and phenotypic distribution. (**A**) *dac-1* cDNA transcript indicating the relative location of fragments targeted for dsRNA synthesis. (**B**) Phenotypic distribution of embryos from first to fourth clutches. Numbers above bars indicate sample sizes. Colors correspond to the legend at the bottom. (**C**)–(**E**) Dead phenotype in *dac-1* RNAi result. (**F**) *dac-1* RNAi causes the loss of spinnerets at the O4–O5 and limb buds at the O2–O3. (**G**) *dac-1* RNAi causes the deletion of the medial part of the pedipalps and walking legs. (**H**) RNAi causes the loss of spinnerets on the right side of O4–O5. as, anterior spinneret bud; ps, posterior spinneret bud; O, opisthosomal segment; L, walking legs. (**I**) HCRs showing wild-type expression of *dac-1* (white) at embryonic stage 9 in the developing legs. (**J**) and (**K**) *Ptep-dac-1* RNAi embryos showing disrupted *dac-1* expression (white) in the developing legs, indistinguishable from background levels. Specimens of HCR are counterstained with DAPI (blue) (scale bars: 100 μm).

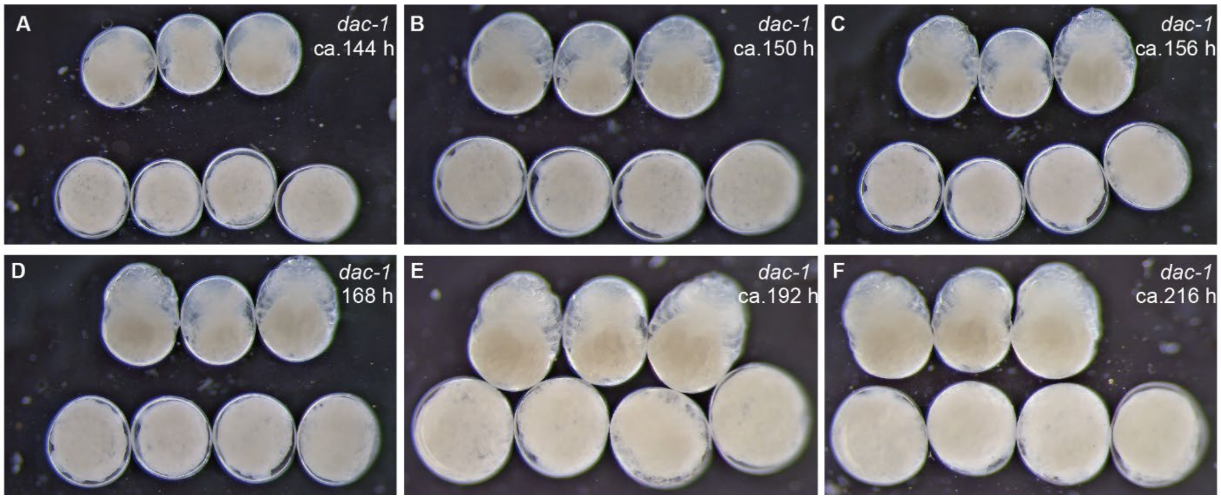

Fig. S30. Development of the *Ptep-dac-1* RNAi embryos of *P. tepidariorum*. (**A**)–(**B**) Embryos were collected from cocoons 4. Stage 14.1 (141–155 h). (**C**)–(**D**) Stage 14.2 (156–185 h). (**E**)–(**F**) Eclosion and postembryo (>186 h). The high lethality of RNAi embryos is associated with strong phenotypic defects.

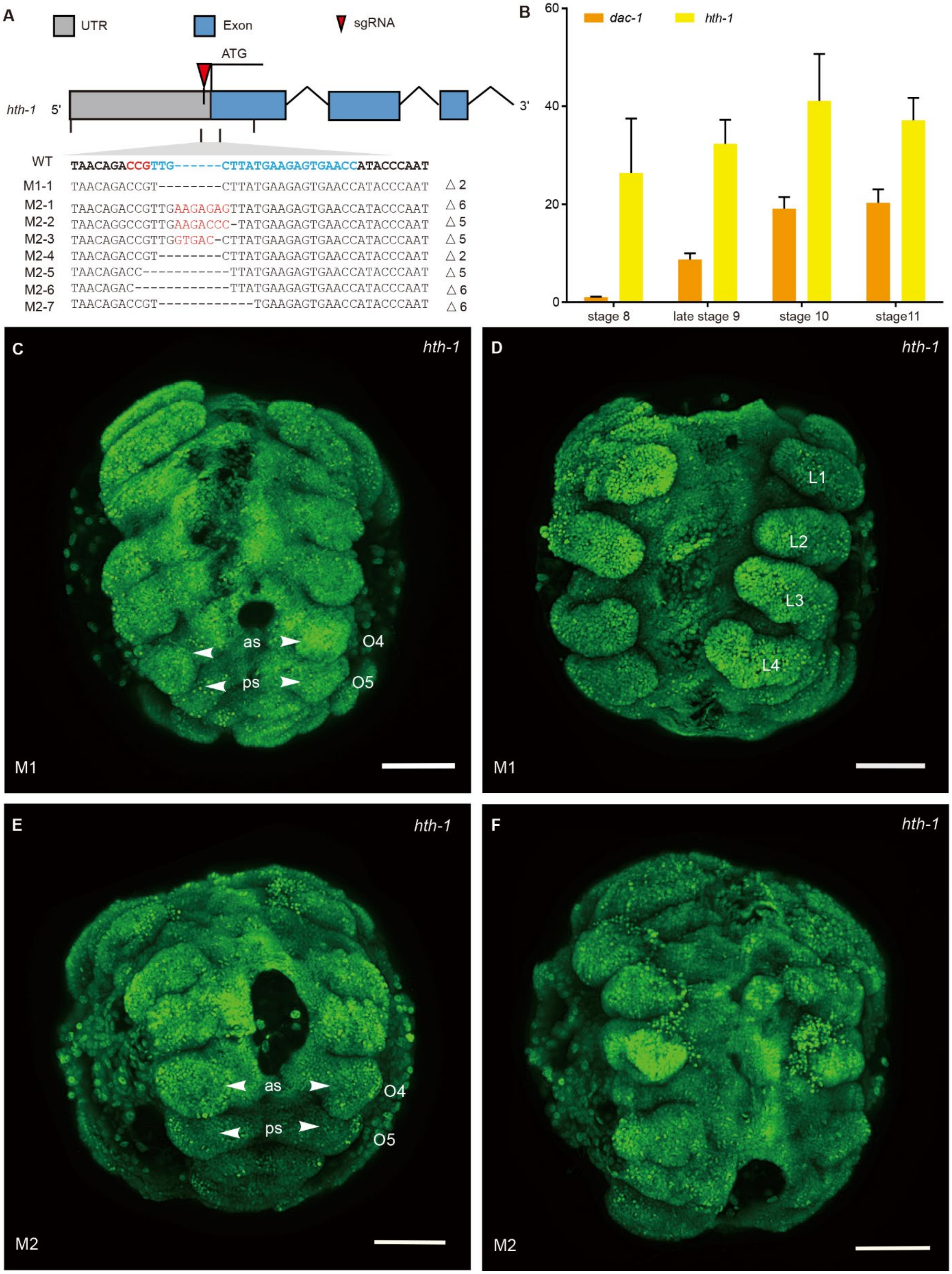

Fig. S31. CRISPR/Cas9 deletions in the gene *hth-1*. (**A**) Location of sgRNAs and the mutated

sequences of the *P. tepidariorum hth-1* locus. Sequences of selected deletion alleles from the embryos (M1 and M2) confirm disruption of *hth-1*. (**B**) Expression of *dac-1* and *hth-1* at different stages from quantitative reverse transcription PCR. Error bars represent the SDs from eight independent measurements. (**C**) and (**D**) No spinneret phenotype after the knockout of *hth-1*. (**E**) and (**F**) *hth-1* deletions result in truncated prosomal appendages. as, anterior spinneret bud; ps, posterior spinneret bud; O, opisthosomal segment; L, walking legs (Scale bars: 100 μm).

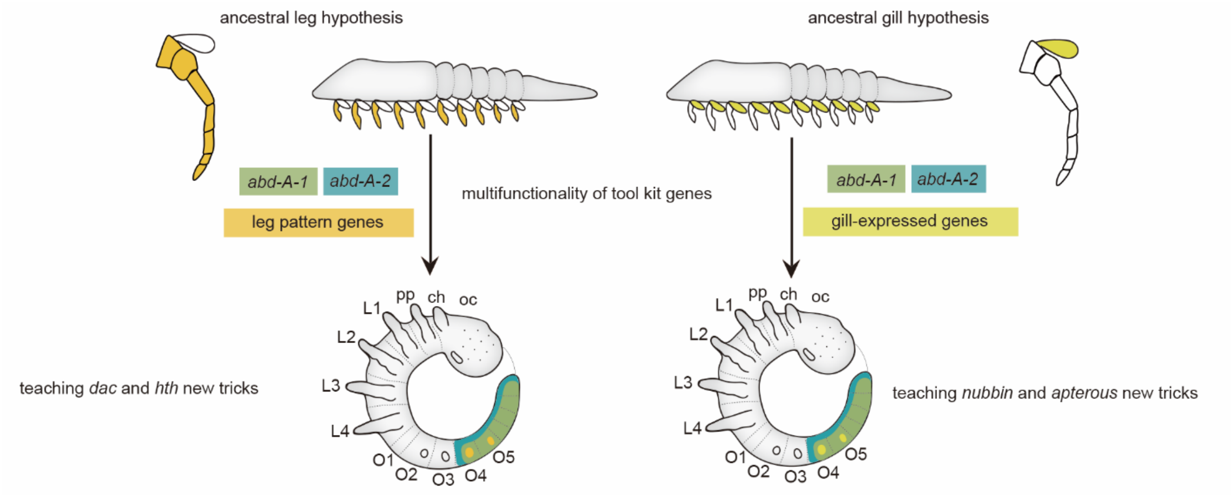

Fig. S32. Two competing scenarios along the P-D axis—the gill hypothesis and the leg hypothesis—reflect how Hox genes modify complex gene networks to transform one tissue into another.

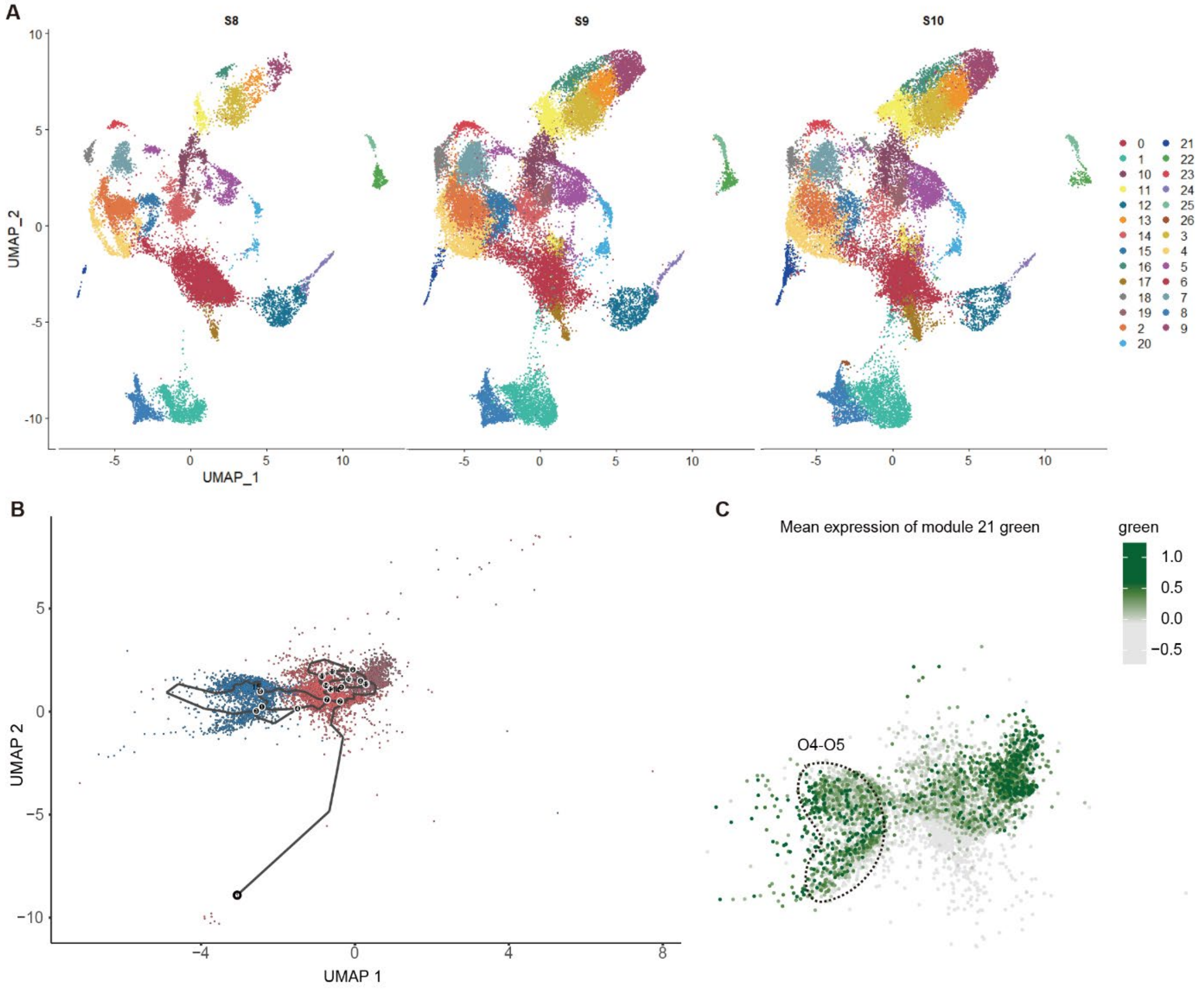

Fig. S33. The development of the embryo at single-cell resolution. (**A**) Uniform manifold approximation and projection (UMAP) of Stages 8, 9, and 10 shows a general increase in data structure through developmental time. (**B**) Pseudotime trajectory of opisthosomal cells. Each dot indicates a single cell, color-coded by the cluster as in Figure 3C. The circles with numbers in them denote special points within the pseudotime trajectory. (**C**) The coexpression module 21 significantly correlated with spinneret development.

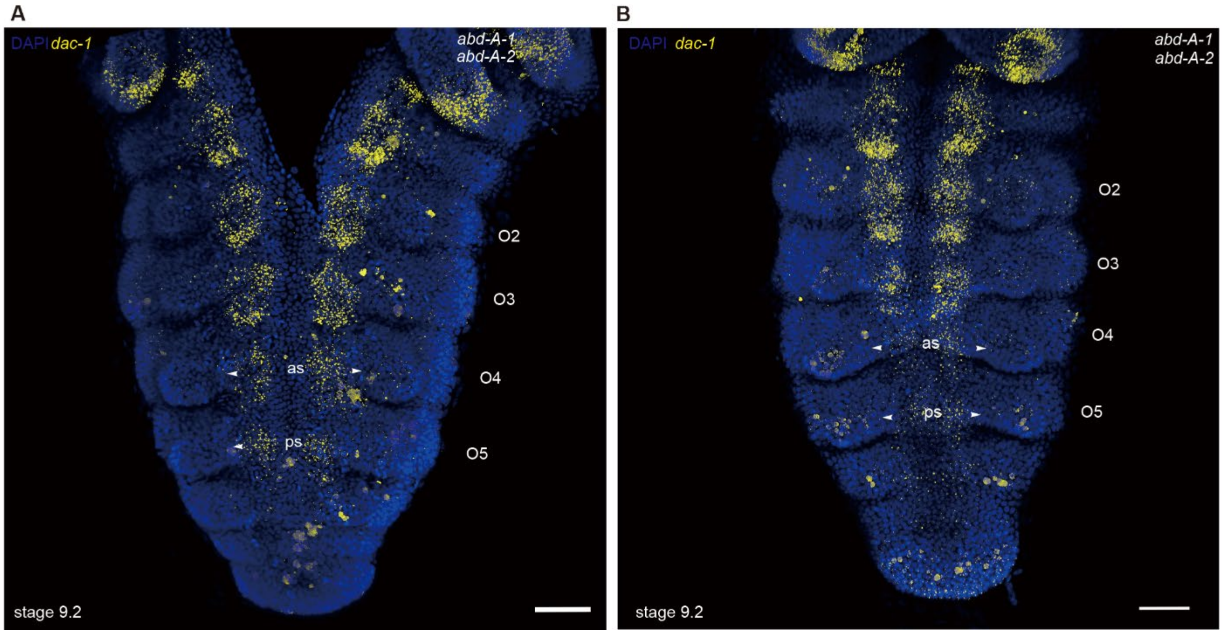

Fig. S34. HCR analysis of *dac-1* in spiders. (**A**) and (**B**) Weak phenocopies from *abd-A-1* and *abd-A-2* RNAi showing diminished *dac-1* expression at embryonic stage 9.2 in the developing limb buds from O2 to O5. Specimens are counterstained with DAPI (scale bars: 50 µm).

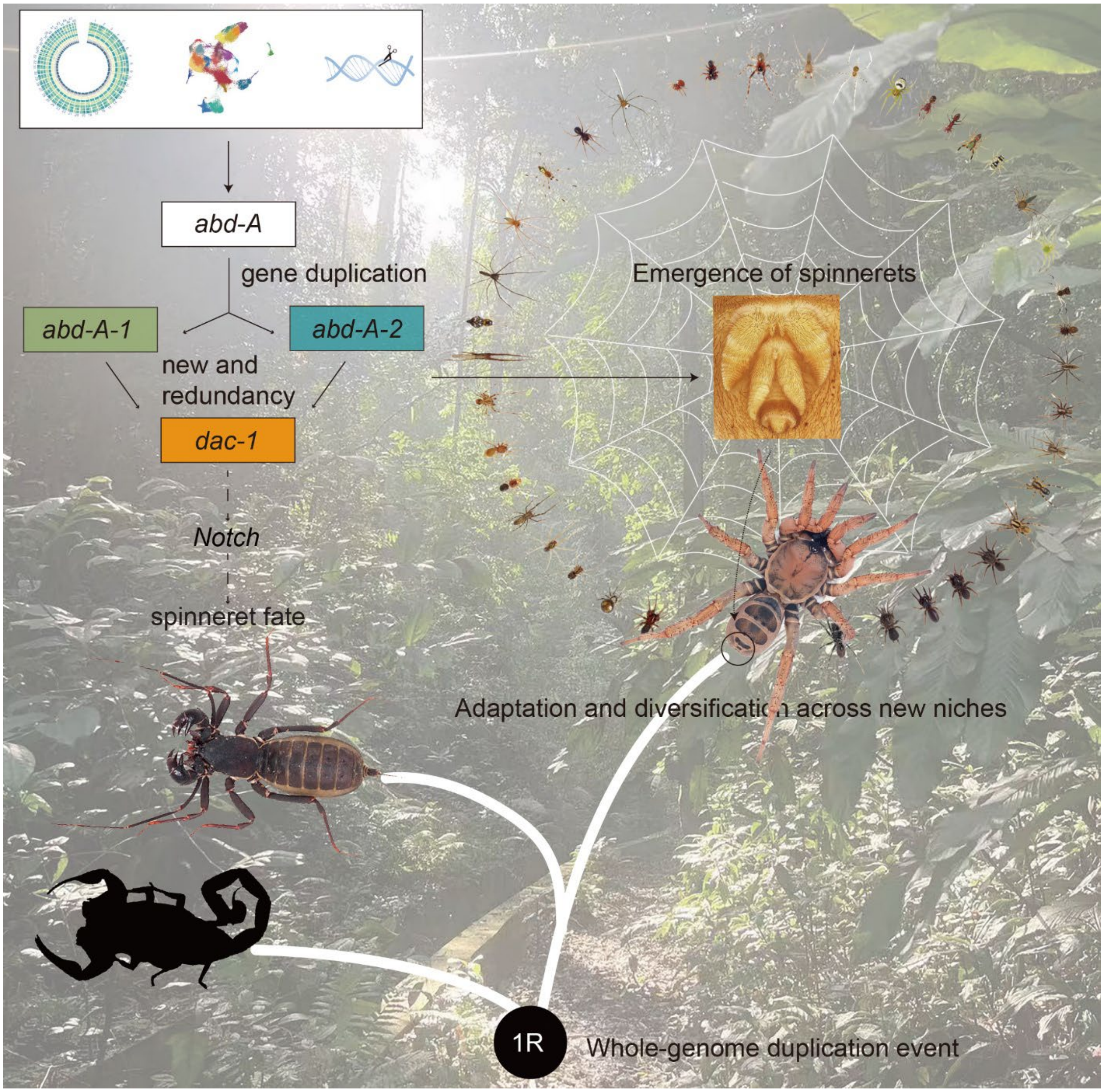

Fig. S35. Arachnopulmonata genome evolution timescale. The ancient WGD event provided arachnopulmonata with a transient survival advantage to cope with the dramatic environmental changes during the late Silurian and offered long-term evolutionary potential, enabling spiders to occupy ecological niches further.

Supplementary Tables

Table S1. The BUSCO assessment results of *Typopeltis vanoorti*.

Table S2. The BUSCO assessment results of *Luthela beijing*.

Table S3. The BUSCO assessment results of *Atypus* sp.

Table S4. The BUSCO assessment results of *Luthela beijing*, *Typopeltis vanoorti*, and *Atypus* sp.

Table S5. List of species used for phylogenetic analysis.

Table S6. The calibration points are from fossil specimens.

Table S7. Homologous gene sets used to test the WGD hypotheses on the chelicerate phylogeny.

Table S8. Summary Statistics of Whale.

Table S9. Probe Pairs for fluorescent in situ hybridization.

Table S10. Injection information of CRISPR experiments for this study.

Table S11. Prime sequences used in research

Table S12. Measurement of bud size in wild and mutated embryos.

Table S13. Injection information of *Ptep-abd-A-1* and *Ptep-abd-A-2* RNAi experiments for this study.

Table S14. Positive selection and relaxation of constraint in *abd-A*. No positive selection signatures were detected in the selected five nodes of the phylogenetic tree using the branch-site model. We found that both paralogs of spider *abd-A* convergently experienced relaxation of constraint after the divergence of spiders and scorpions.

Table S15. Maker genes and annotation results of each cluster in single-cell data.

Table S16. Injection information of *Ptep-dac-1* RNAi experiments for this study.

Table S17. Results of single-cell analyses, related to Figure 4.

Table S18. Differentially expressed genes between O4–O5 and O2–O3, related to Figure 4.

Table S19. Differentially expressed genes between O4–O5 and SAZ, related to Figure 4.

Table S20.Target genes for each TF.

REFERENCES

1. J. L. Yarger, B. Cherry, A. van der Vaart, Uncovering the structure–function relationship in spider silk. *Nat. Rev. Mater.* **3**, 18008 (2018).

2. S. Wu, Z. Liu, C. Gong, W. Li, S. Xu, R. Wen, W. Feng, Z. Qiu, Y. Yan, Spider-silk-inspired strong and tough hydrogel fibers with anti-freezing and water retention properties. *Nat. Commun.* **15**, 4441 (2024).

3. M. Pechmann, N. M. Prpic, Appendage patterning in the South American bird spider *Acanthoscurria geniculata* (Araneae: Mygalomorphae). *Dev. Genes Evol.* **219**, 189–198 (2009).

4. R. Janssen, M. Pechmann, Expression of posterior Hox genes and opisthosomal appendage development in a mygalomorph spider. *Dev. Genes Evol.* **233**, 107–121 (2023).

5. W. G. Damen, T. Saridaki, M. Averof, Diverse adaptations of an ancestral gill: A common evolutionary origin for wings, breathing organs, and spinnerets. *Curr. Biol.* **12**, 1711–1716 (2002).

6. E. B. Lewis, A gene complex controlling segmentation in *Drosophila*. *Nature* **276**, 565–570 (1978).

7. S. Khadjeh, N. Turetzek, M. Pechmann, E. E. Schwager, E. A. Wimmer, W. G. Damen, N. M. Prpic, Divergent role of the Hox gene *Antennapedia* in spiders is responsible for the convergent evolution of abdominal limb repression. *Proc. Natl. Acad. Sci. U.S.A.* **109**, 4921–4926 (2012).

8. E. V. W. Setton, P. P. Sharma, Cooption of an appendage-patterning gene cassette in the head segmentation of arachnids. *Proc. Natl. Acad. Sci. U.S.A.* **115**, E3491–E3500 (2018).

9. M. Stauber, A. Prell, U. Schmidt-Ott, A single *Hox3* gene with composite *bicoid* and *zerknullt* expression characteristics in non-Cyclorrhaphan flies. *Proc. Natl. Acad. Sci. U.S.A.* **99**, 274–279 (2002).

10. J. Copeland, A. Nasiadka, B. Dietrich, H. Krause, Patterning of the *Drosophila* embryo by a homeodomain-deleted Ftz polypeptide. *Nature* **379**, 162–165 (1996).

11. P. W. Holland, F. Marletaz, I. Maeso, T. L. Dunwell, J. Paps, New genes from old: Asymmetric divergence of gene duplicates and the evolution of development. *Philos. Trans. R. Soc. Lond. B Biol. Sci.* **372**, 20150480 (2017).

12. E. E. Schwager, P. P. Sharma, T. Clarke, D. J. Leite, T. Wierschin, M. Pechmann, Y. Akiyama-Oda, L. Esposito, J. Bechsgaard, T. Bilde, A. D. Buffry, H. Chao, H. Dinh, H. Doddapaneni, S. Dugan, C. Eibner, C. G. Extavour, P. Funch, J. Garb, L. B. Gonzalez, V. L. Gonzalez, S. Griffiths-Jones, Y. Han, C. Hayashi, M. Hilbrant, D. S. T. Hughes, R. Janssen, S. L. Lee, I. Maeso, S. C. Murali, D. M. Muzny, R. Nunes da Fonseca, C. L. B. Paese, J. Qu, M. Ronshaugen, C. Schomburg, A. Schönauer, A. Stollewerk, M. Torres-Oliva, N. Turetzek, B. Vanthournout, J. H. Werren, C. Wolff, K. C. Worley, G. Bucher, R. A. Gibbs, J. Coddington, H. Oda, M. Stanke, N. A. Ayoub, N. M. Prpic, J. F. Flot, N. Posnien, S. Richards, A. P. McGregor, The house spider genome reveals an ancient whole-genome duplication during arachnid evolution. *BMC Biol.* **15**, 62 (2017).

13. M. E. Aase-Remedios, R. Janssen, D. J. Leite, L. Sumner-Rooney, A. P. McGregor, Evolution of the spider homeobox gene repertoire by tandem and whole genome duplication. *Mol. Biol. Evol.* **40**, msad239 (2023).

14. D. J. Leite, L. Baudouin-Gonzalez, S. Iwasaki-Yokozawa, J. Lozano-Fernandez, N. Turetzek, Y. Akiyama-Oda, N. M. Prpic, D. Pisani, H. Oda, P. P. Sharma, A. P. McGregor, Homeobox gene duplication and divergence in arachnids. *Mol. Biol. Evol.* **35**, 2240–2253 (2018).

15. C. L. Bonatto Paese, D. J. Leite, A. Schönauer, A. P. McGregor, S. Russell, Duplication and expression of Sox genes in spiders. *BMC Evol. Biol.* **18**, 205 (2018).

16. R. Janssen, M. Pechmann, N. Turetzek, A chelicerate Wnt gene expression atlas: Novel insights into the complexity of arthropod Wnt-patterning. *EvoDevo* **12**, 12 (2021).

17. D. J. Leite, M. Ninova, M. Hilbrant, S. Arif, S. Griffiths-Jones, M. Ronshaugen, A. P. McGregor, Pervasive microRNA duplication in chelicerates: Insights from the embryonic

microRNA repertoire of the spider *Parasteatoda tepidariorum*. *Genome Biol. Evol.* **8**, 2133–2144 (2016).

18. S. S. Kulkarni, B. C. Klementz, P. P. Sharma, A chromosome-level genome of the giant vinegaroon *Mastigoproctus giganteus* exhibits the signature of pre-Silurian whole genome duplication. *J. Hered.* **116**, 279–292 (2025).

19. G. W. C. Thomas, M. T. W. McKibben, M. W. Hahn, M. S. Barker, A comprehensive examination of Chelicerate genomes reveals no evidence for a whole genome duplication among spiders and scorpions. bioRxiv 578966 [Preprint] (2024); www.biorxiv.org/content/10.1101/2024.02.05.578966v1.

20. E. H. Davidson, D. H. Erwin, Gene regulatory networks and the evolution of animal body plans. *Science* **311**, 796–800 (2006).

21. M. Schoppmeier, W. G. Damen, Double-stranded RNA interference in the spider *Cupiennius salei*: The role of *Distal-less* is evolutionarily conserved in arthropod appendage formation. *Dev. Genes Evol.* **211**, 76–82 (2001).

22. Y. Van de Peer, E. Mizrachi, K. Marchal, The evolutionary significance of polyploidy. *Nat. Rev. Genet.* **18**, 411–424 (2017).

23. J. A. Ballesteros, C. E. Santibáñez-López, C. M. Baker, L. R. Benavides, T. J. Cunha, G. Gainett, A. Z. Ontano, E. V. W. Setton, C. P. Arango, E. Gavish-Regev, M. S. Harvey, W. C. Wheeler, G. Hormiga, G. Giribet, P. P. Sharma, Comprehensive species sampling and sophisticated algorithmic approaches refute the monophyly of Arachnida. *Mol. Biol. Evol.* **39**, msac021 (2022).

24. W. A. Shear, J. M. Palmer, J. A. Coddington, P. M. Bonamo, A devonian spinneret: Early evidence of spiders and silk use. *Science* **246**, 479–481 (1989).

25. O. Simakov, J. Bredeson, K. Berkoff, F. Marletaz, T. Mitros, D. T. Schultz, B. L. O'Connell, P. Dear, D. E. Martinez, R. E. Steele, R. E. Green, C. N. David, D. S. Rokhsar, Deeply

conserved synteny and the evolution of metazoan chromosomes. *Sci. Adv.* **8**, eabi5884 (2022).

26. D. T. Schultz, S. H. D. Haddock, J. V. Bredeson, R. E. Green, O. Simakov, D. S. Rokhsar, Ancient gene linkages support ctenophores as sister to other animals. *Nature* **618**, 110–117 (2023).

27. A. Zwaenepoel, Y. Van de Peer, Inference of ancient whole-genome duplications and the evolution of gene duplication and loss rates. *Mol. Biol. Evol.* **36**, 1384–1404 (2019).

28. A. Harper, L. Baudouin Gonzalez, A. Schönauer, R. Janssen, M. Seiter, M. Holzem, S. Arif, A. P. McGregor, L. Sumner-Rooney, Widespread retention of ohnologs in key developmental gene families following whole-genome duplication in arachnopulmonates. *G3 (Bethesda)* **11**, jkab299 (2021).

29. Z. Yang, PAML 4: Phylogenetic analysis by maximum likelihood. *Mol. Biol. Evol.* **24**, 1586–1591 (2007).

30. X. Gu, Y. Zou, Z. Su, W. Huang, Z. Zhou, Z. Arendsee, Y. Zeng, An update of DIVERGE software for functional divergence analysis of protein family. *Mol. Biol. Evol.* **30**, 1713–1719 (2013).

31. B. Mittmann, C. Wolff, Embryonic development and staging of the cobweb spider *Parasteatoda tepidariorum* C. L. Koch, 1841 (syn.: *Achaearanea tepidariorum*; Araneomorphae; Theridiidae). *Dev. Genes Evol.* **222**, 189–216 (2012).

32. Y. Hu, S. G. Tattikota, Y. Liu, A. Comjean, Y. Gao, C. Forman, G. Kim, J. Rodiger, I. Papatheodorou, G. Dos Santos, S. E. Mohr, N. Perrimon, DRscDB: A single-cell RNA-seq resource for data mining and data comparison across species. *Comput. Struct. Biotechnol. J.* **19**, 2018–2026 (2021).

33. D. J. Leite, A. Schönauer, G. Blakeley, A. Harper, H. Garcia-Castro, L. Baudouin-Gonzalez, R. Wang, N. Sarkis, A. G. Nikola, V. S. P. Koka, N. J. Kenny, N. Turetzek, M. Pechmann, J.

Solana, A. P. McGregor, An atlas of spider development at single-cell resolution provides new insights into arthropod embryogenesis. *EvoDevo* **15**, 5 (2024).

34. N. Turetzek, M. Pechmann, C. Schomburg, J. Schneider, N. M. Prpic, Neofunctionalization of a duplicate *dachshund* gene underlies the evolution of a novel leg segment in arachnids. *Mol. Biol. Evol.* **33**, 109–121 (2016).

35. B. C. Klementz, G. Brenneis, I. A. Hinne, E. M. Laumer, S. M. Neu, G. M. Hareid, G. Gainett, E. V. W. Setton, C. Simian, D. E. Vrech, I. Joyce, A. A. Barnett, N. H. Patel, M. S. Harvey, A. V. Peretti, M. Gulia-Nuss, P. P. Sharma, A novel expression domain of *extradenticle* underlies the evolutionary developmental origin of the chelicerate patella. *Mol. Biol. Evol.* **41**, msae188 (2024).

36. N. Turetzek, S. Khadjeh, C. Schomburg, N.-M. Prpic, Rapid diversification of *homothorax* expression patterns after gene duplication in spiders. *BMC Evol. Biol.* **17**, 168 (2017).

37. C. Feregrino, P. Tschopp, Assessing evolutionary and developmental transcriptome dynamics in homologous cell types. *Dev. Dyn.* **251**, 1472–1489 (2022).

38. S. Morabito, E. Miyoshi, N. Michael, S. Shahin, A. C. Martini, E. Head, J. Silva, K. Leavy, M. Perez-Rosendahl, V. Swarup, Single-nucleus chromatin accessibility and transcriptomic characterization of Alzheimer's disease. *Nat. Genet.* **53**, 1143–1155 (2021).

39. V. A. Huynh-Thu, A. Irrthum, L. Wehenkel, P. Geurts, Inferring regulatory networks from expression data using tree-based methods. *PLOS ONE* **5**, e12776 (2010).

40. P. Shannon, A. Markiel, O. Ozier, N. S. Baliga, J. T. Wang, D. Ramage, N. Amin, B. Schwikowski, T. Ideker, Cytoscape: A software environment for integrated models of biomolecular interaction networks. *Genome Res.* **13**, 2498–2504 (2003).

41. F. Marletaz, N. Timoshevskaya, V. A. Timoshevskiy, E. Parey, O. Simakov, D. Gavriouchkina, M. Suzuki, K. Kubokawa, S. Brenner, J. J. Smith, D. S. Rokhsar, The hagfish genome and the evolution of vertebrates. *Nature* **627**, 811–820 (2024).

42. D. Yu, Y. Ren, M. Uesaka, A. J. S. Beavan, M. Muffato, J. Shen, Y. Li, I. Sato, W. Wan, J. W. Clark, J. N. Keating, E. M. Carlisle, R. P. Dearden, S. Giles, E. Randle, R. S. Sansom, R. Feuda, J. F. Fleming, F. Sugahara, C. Cummins, M. Patricio, W. Akanni, S. D'Aniello, C. Bertolucci, N. Irie, C. Alev, G. Sheng, A. de Mendoza, I. Maeso, M. Irimia, B. Fromm, K. J. Peterson, S. Das, M. Hirano, J. P. Rast, M. D. Cooper, J. Paps, D. Pisani, S. Kuratani, F. J. Martin, W. Wang, P. C. J. Donoghue, Y. E. Zhang, J. Pascual-Anaya, Hagfish genome elucidates vertebrate whole-genome duplication events and their evolutionary consequences. *Nat. Ecol. Evol.* **8**, 519–535 (2024).

43. T. R. Gregory, *The Evolution of the Genome* (Elsevier, 2011).

44. D. Huang, G. Hormiga, C. Cai, Y. Su, Z. Yin, F. Xia, G. Giribet, Origin of spiders and their spinning organs illuminated by mid-Cretaceous amber fossils. *Nat. Ecol. Evol.* **2**, 623–627 (2018).

45. M. Pechmann, S. Khadjeh, F. Sprenger, N. M. Prpic, Patterning mechanisms and morphological diversity of spider appendages and their importance for spider evolution. *Arthropod Struct. Dev.* **39**, 453–467 (2010).

46. N. M. Prpic, W. G. Damen, *Notch*-mediated segmentation of the appendages is a molecular phylotypic trait of the arthropods. *Dev. Biol.* **326**, 262–271 (2009).

47. S. Sonavane, S. Hassan, U. Chatterjee, L. Soler, L. Holm, A. Mollbrink, G. Greco, N. Fereydouni, O. Vinnere Pettersson, I. Bunikis, A. Churcher, H. Lantz, J. Johansson, J. Reimegård, A. Rising, Origin, structure, and composition of the spider major ampullate silk fiber revealed by genomics, proteomics, and single-cell and spatial transcriptomics. *Sci. Adv.* **10**, eadn0597 (2024).

48. F. Vollrath, D. P. Knight, Liquid crystalline spinning of spider silk. *Nature* **410**, 541–548 (2001).

49. S. S. Rao, M. H. Huntley, N. C. Durand, E. K. Stamenova, I. D. Bochkov, J. T. Robinson, A. L. Sanborn, I. Machol, A. D. Omer, E. S. Lander, E. L. Aiden, A 3D map of the human

genome at kilobase resolution reveals principles of chromatin looping. *Cell* **159**, 1665–1680 (2014).

50. P. Jin, B. Zhu, Y. Jia, Y. Zhang, W. Wang, Y. Shen, Y. Zhong, Y. Zheng, Y. Wang, Y. Tong, W. Zhang, S. Li, Single-cell transcriptomics reveals the brain evolution of web-building spiders. *Nat. Ecol. Evol.* **7**, 2125–2142 (2023).

51. L. Lu, X. Liu, W. K. Huang, P. Giusti-Rodriguez, J. Cui, S. Zhang, W. Xu, Z. Wen, S. Ma, J. D. Rosen, Z. Xu, C. F. Bartels, R. Kawaguchi, M. Hu, P. C. Scacheri, Z. Rong, Y. Li, P. F. Sullivan, H. Song, G. L. Ming, Y. Li, F. Jin, Robust Hi-C maps of enhancer-promoter interactions reveal the function of non-coding genome in neural development and diseases. *Mol. Cell* **79**, 521–534.e15 (2020).

52. M. Meyer, M. Kircher, Illumina sequencing library preparation for highly multiplexed target capture and sequencing. *Cold Spring Harbor Protoc.* **2010**, pdb.prot5448 (2010).

53. N. C. Durand, M. S. Shamim, I. Machol, S. S. Rao, M. H. Huntley, E. S. Lander, E. L. Aiden, Juicer provides a one-click system for analyzing loop-resolution Hi-C experiments. *Cell* **3**, 95–98 (2016).

54. O. Dudchenko, S. S. Batra, A. D. Omer, S. K. Nyquist, M. Hoeger, N. C. Durand, M. S. Shamim, I. Machol, E. S. Lander, A. P. Aiden, E. L. Aiden, De novo assembly of the *Aedes aegypti* genome using Hi-C yields chromosome-length scaffolds. *Science* **356**, 92–95 (2017).

55. O. Dudchenko, M. S. Shamim, S. S. Batra, N. C. Durand, N. T. Musial, R. Mostofa, M. Pham, B. G. S. Hilaire, W. Yao, E. Stamenova, M. Hoeger, S. K. Nyquist, V. Korchina, K. Pletch, J. P. Flanagan, A. Tomaszewicz, D. McAloose, C. P. Estrada, B. J. Novak, A. D. Omer, E. L. Aiden, The Juicebox Assembly Tools module facilitates de novo assembly of mammalian genomes with chromosome-length scaffolds for under $1000. bioRxiv 254797 [Preprint] (2018); www.biorxiv.org/content/10.1101/254797v1.

56. H. Cheng, G. T. Concepcion, X. Feng, H. Zhang, H. Li, Haplotype-resolved de novo assembly using phased assembly graphs with hifiasm. *Nat. Methods* **18**, 170–175 (2021).

57. J. Ruan, H. Li, Fast and accurate long-read assembly with wtdbg2. *Nat. Methods* **17**, 155–158 (2020).

58. J. Hu, J. Fan, Z. Sun, S. Liu, NextPolish: A fast and efficient genome polishing tool for long-read assembly. *Bioinformatics* **36**, 2253–2255 (2020).

59. M. Seppey, M. Manni, E. M. Zdobnov, BUSCO: Assessing genome assembly and annotation completeness. *Methods Mol. Biol.* **1962**, 227–245 (2019).

60. J. M. Flynn, R. Hubley, C. Goubert, J. Rosen, A. G. Clark, C. Feschotte, A. F. Smit, RepeatModeler2 for automated genomic discovery of transposable element families. *Proc. Natl. Acad. Sci. U.S.A.* **117**, 9451–9457 (2020).

61. M. Tarailo-Graovac, N. Chen, Using RepeatMasker to identify repetitive elements in genomic sequences. *Curr. Protoc. Bioinformatics* **25**, 4.10.1–14.10.14 (2009).

62. D. M. Emms, S. Kelly, OrthoFinder: Phylogenetic orthology inference for comparative genomics. *Genome Biol.* **20**, 238 (2019).

63. K. Katoh, D. M. Standley, MAFFT multiple sequence alignment software version 7: Improvements in performance and usability. *Mol. Biol. Evol.* **30**, 772–780 (2013).

64. S. F. Altschul, T. L. Madden, A. A. Schäffer, J. Zhang, Z. Zhang, W. Miller, D. J. Lipman, Gapped BLAST and PSI-BLAST: A new generation of protein database search programs. *Nucleic Acids Res.* **25**, 3389–3402 (1997).

65. M. N. Price, P. S. Dehal, A. P. Arkin, FastTree 2—Approximately maximum-likelihood trees for large alignments. *PLOS ONE* **5**, e9490 (2010).

66. I. L. F. Magalhaes, G. H. F. Azevedo, P. Michalik, M. J. Ramírez, The fossil record of spiders revisited: Implications for calibrating trees and evidence for a major faunal turnover since the Mesozoic. *Biol. Rev. Camb. Philos. Soc.* **95**, 184–217 (2020).

67. P. A. Selden, W. A. Shear, M. D. Sutton, Fossil evidence for the origin of spider spinnerets, and a proposed arachnid order. *Proc. Natl. Acad. Sci. U.S.A.* **105**, 20781–20785 (2008).

68. M. V. Han, G. W. Thomas, J. Lugo-Martinez, M. W. Hahn, Estimating gene gain and loss rates in the presence of error in genome assembly and annotation using CAFE 3. *Mol. Biol. Evol.* **30**, 1987–1997 (2013).

69. A. Zwaenepoel, Y. Van de Peer, wgd—Simple command line tools for the analysis of ancient whole-genome duplications. *Bioinf. (Oxf.)* **35**, 2153–2155 (2019).

70. P. Kück, G. C. Longo, FASconCAT-G: Extensive functions for multiple sequence alignment preparations concerning phylogenetic studies. *Front. Zool.* **11**, 81 (2014).

71. B. Q. Minh, H. A. Schmidt, O. Chernomor, D. Schrempf, M. D. Woodhams, A. von Haeseler, R. Lanfear, IQ-TREE 2: New models and efficient methods for phylogenetic inference in the genomic era. *Mol. Biol. Evol.* **37**, 1530–1534 (2020).

72. K. Kruse, C. B. Hug, J. M. Vaquerizas, FAN-C: A feature-rich framework for the analysis and visualisation of chromosome conformation capture data. *Genome Biol.* **21**, 303 (2020).

73. J. P. Lloyd, Z. T. Tsai, R. P. Sowers, N. L. Panchy, S. H. Shiu, A model-based approach for identifying functional intergenic transcribed regions and noncoding RNAs. *Mol. Biol. Evol.* **35**, 1422–1436 (2018).

74. S. Xie, B. Shen, C. Zhang, X. Huang, Y. Zhang, sgRNAcas9: A software package for designing CRISPR sgRNA and evaluating potential off-target cleavage sites. *PLOS ONE* **9**, e100448 (2014).

75. L. Zhang, R. D. Reed, in *Diversity and Evolution of Butterfly Wing Patterns: An Integrative Approach*, T. Sekimura, H. F. Nijhout, Eds. (Springer, 2017), pp. 155–172.

76. B. Zhu, P. Jin, Y. Zhang, Y. Shen, W. Wang, S. Li, Genomic and transcriptomic analyses support a silk gland origin of spider venom glands. *BMC Biol.* **21**, 82 (2023).

77. Y. Hao, S. Hao, E. Andersen-Nissen, W. M. Mauck III, S. Zheng, A. Butler, M. J. Lee, A. J. Wilk, C. Darby, M. Zager, P. Hoffman, M. Stoeckius, E. Papalexi, E. P. Mimitou, J. Jain, A. Srivastava, T. Stuart, L. M. Fleming, B. Yeung, A. J. Rogers, J. M. McElrath, C. A. Blish, R.

Gottardo, P. Smibert, R. Satija, Integrated analysis of multimodal single-cell data. *Cell* **184**, 3573–3587.e29 (2021).

78. C. H. Chin, S. H. Chen, H. H. Wu, C. W. Ho, M. T. Ko, C. Y. Lin, cytoHubba: Identifying hub objects and sub-networks from complex interactome. *BMC Syst. Biol.* **8**, S11 (2014).

79. L. Kumar, M. E. Futschik, Mfuzz: A software package for soft clustering of microarray data. *Bioinformation* **2**, 5–7 (2007).

80. A. Löytynoja, in *Multiple sequence alignment methods*. (Springer, 2014), pp. 155–170.

81. O. Penn, E. Privman, H. Ashkenazy, G. Landan, D. Graur, T. Pupko, GUIDANCE: A web server for assessing alignment confidence scores. *Nucleic Acids Res.* **38**, W23–W28 (2010).

82. S. Capella-Gutiérrez, J. M. Silla-Martínez, T. Gabaldón, trimAl: A tool for automated alignment trimming in large-scale phylogenetic analyses. *Bioinformatics* **25**, 1972–1973 (2009).

83. A. Stamatakis, RAxML version 8: A tool for phylogenetic analysis and post-analysis of large phylogenies. *Bioinformatics* **30**, 1312–1313 (2014).

84. E. Kuehn, D. S. Clausen, R. W. Null, B. M. Metzger, A. D. Willis, B. D. Özpolat, Segment number threshold determines juvenile onset of germline cluster expansion in *Platynereis dumerilii*. *J. Exp. Zool. B Mol. Dev. Evol.* **338**, 225–240 (2022).

85. H. S. Bruce, G. Jerz, S. R. Kelly, J. McCarthy, A. Pomerantz, G. Senevirathne, A. Sherrard, D. A. Sun, C. Wolff, N. H. Patel, Hybridization chain reaction (HCR) in situ protocol (protocols. io., 2021).

86. H. Oda, Y. Akiyama-Oda, The common house spider *Parasteatoda tepidariorum*. *EvoDevo* **11**, 6 (2020).

87. N. M. Prpic, M. Schoppmeier, W. G. Damen, Collection and fixation of spider embryos. *CSH Protoc.* **2008**, pdb.prot5067 (2008).
